# Efficient Detection of the Alternative Spliced Human Proteome Using Translatome Sequencing

**DOI:** 10.3389/fmolb.2022.895746

**Published:** 2022-06-02

**Authors:** Chun Wu, Xiaolong Lu, Shaohua Lu, Hongwei Wang, Dehua Li, Jing Zhao, Jingjie Jin, Zhenghua Sun, Qing-Yu He, Yang Chen, Gong Zhang

**Affiliations:** ^1^ Key Laboratory of Functional Protein Research of Guangdong Higher Education Institutes and MOE Key Laboratory of Tumor Molecular Biology, Institute of Life and Health Engineering, Jinan University, Guangzhou, China; ^2^ State Key Laboratory of Respiratory Disease, School of Basic Medical Sciences, Sino-French Hoffmann Institute, Guangzhou Medical University, Guangzhou, China

**Keywords:** alternative splicing, translatome sequencing, mass spectrometry, proteome, isoform, human proteome project

## Abstract

Alternative splicing (AS) isoforms create numerous proteoforms, expanding the complexity of the genome. Highly similar sequences, incomplete reference databases and the insufficient sequence coverage of mass spectrometry limit the identification of AS proteoforms. Here, we demonstrated full-length translating mRNAs (ribosome nascent-chain complex-bound mRNAs, RNC-mRNAs) sequencing (RNC-seq) strategy to sequence the entire translating mRNA using next-generation sequencing, including short-read and long-read technologies, to construct a protein database containing all translating AS isoforms. Taking the advantage of read length, short-read RNC-seq identified up to 15,289 genes and 15,906 AS isoforms in a single human cell line, much more than the Ribo-seq. The single-molecule long-read RNC-seq supplemented 4,429 annotated AS isoforms that were not identified by short-read datasets, and 4,525 novel AS isoforms that were not included in the public databases. Using such RNC-seq-guided database, we identified 6,766 annotated protein isoforms and 50 novel protein isoforms in mass spectrometry datasets. These results demonstrated the potential of full-length RNC-seq in investigating the proteome of AS isoforms.

## Introduction

A single human gene can produce a variety of alternative splicing (AS) isoforms, which may be translated into protein isoforms with different localizations, structures and functions, which dramatically diversify the transcriptome and proteome. Proteoforms from AS can have different functional domains, such as enzymatic active sites or protein-binding sites, and participate in various important physiological and pathological processes. A lot of evidence shows that AS disorders lead to various diseases (Wang et al., 2008; Baralle and Giudice, 2017). Therefore, a major add-on challenge of Human Proteome Project (HPP) over Human Genome Project is to discover protein AS isoforms (Paik et al., 2012).

With the development of next-generation sequencing technology, more than 100,000 AS isoforms have been found in human genome (Pan et al., 2008). However, it has been shown that different AS transcripts are translated in different efficiency, some of which are not translated (Wang et al., 2013). Identifying protein products of the AS isoforms and characterizing their functions remain a huge challenge because of two main reasons: 1) Protein isoforms from AS are usually highly similar in sequences and thus may be difficult to distinguish using mass spectrometry (MS) techniques. High sequence homologies and similarity physical-chemical properties of protein isoforms make it difficult for them to be effectively separated by pre-fractionation steps, and unique peptides generated by the digestion of low-expression proteins is hard to be identified by MS. The average sequence coverage of a recent near-complete yeast proteome is only 29% when using trypsin digestion (Gao et al., 2021). The sequence coverage of human proteome is 14–25%, which hinders the discovery of unique peptides of AS isoforms (Wang et al., 2019). 2) Many public proteome reference databases tend to include only the canonical isoforms. The AS isoforms are largely missing or incomplete (Sulakhe et al., 2019). Moreover, protein isoforms sequences are largely inconsistent across all the commonly used databases. In comparison with the canonical isoforms, the sequence features in the human alternatively splicing isoforms always be lost or modified (Frankish et al., 2015).

We previously exhibited that the translatome sequencing, i.e. next-generation sequencing on the translating mRNAs, provides a powerful tool to investigate the proteins which are being synthesized (Zhong et al., 2014). Due to the high throughput of translatome sequencing techniques, it is relatively easy to achieve near-complete sequence coverage of translating mRNAs, thus provides a solid basis of the analysis on protein isoforms and single amino-acid polymorphisms. There are two major translatome sequencing techniques, RNC-seq and Ribo-seq (reviewed in (Zhao et al., 2019)). In brief, Ribo-seq use ribonuclease to digest mRNA excluded by ribosomes into ribosome protected fragments (RPFs), also known as ribosome footprints (RFPs), ∼28 nt in average in eukaryotic cells. It is proposed to identify the non-canonical translation initiation or termination, the truncation or extension of reading frame and uORF, etc. However, Ribo-seq seems to show high false positives in ORF detection in practice (Guttman et al., 2013; Lu et al., 2019). In contrast, RNC-seq sequences the entire mRNA in the ribosome nascent-chain complex. Since the mRNA is intact, the sequencing library can be of any size. Therefore, RNC-seq has some following advantages in detecting translating AS isoforms. 1) The long reads can easily exclude the small ribosome-engaged RNA fragment contaminants by the longer library length and poly(A)-enrichment strategy, minimizing the false positives. 2) The long reads have a greater chance of span across the splice junction and thus enable alignments to process reads across junctions more accurately, which means RNC-seq can theoretically detect more AS isoforms. 3) The unlimited length of insert fragment can reveal novel isoforms and guide the discovery of novel proteins isoforms by long-read sequencing. 4) The experimental process of RNC-seq without enzymic digestion is simpler. Therefore, the result of RNC-seq has the advantages of better stability and reproducibility. RNC-seq has found a “hidden proteome”, i.e. a large number of proteins encoded by “non-coding” RNAs (ncRNAs), demonstrating that RNC-seq is an effective method to guide new protein identification (Lu et al., 2019).

In this work, we systematically compared RNC-seq and Ribo-seq in the context of proteome identification, especially when identifying protein isoforms from AS. We also demonstrated that the single-molecule long read sequencing technique identified thousands of new splice variants and guided the MS identifications of new protein isoforms.

## Results

### Translating AS Detection Efficiency of RNC-Seq and Ribo-Seq

**FIGURE 1:**
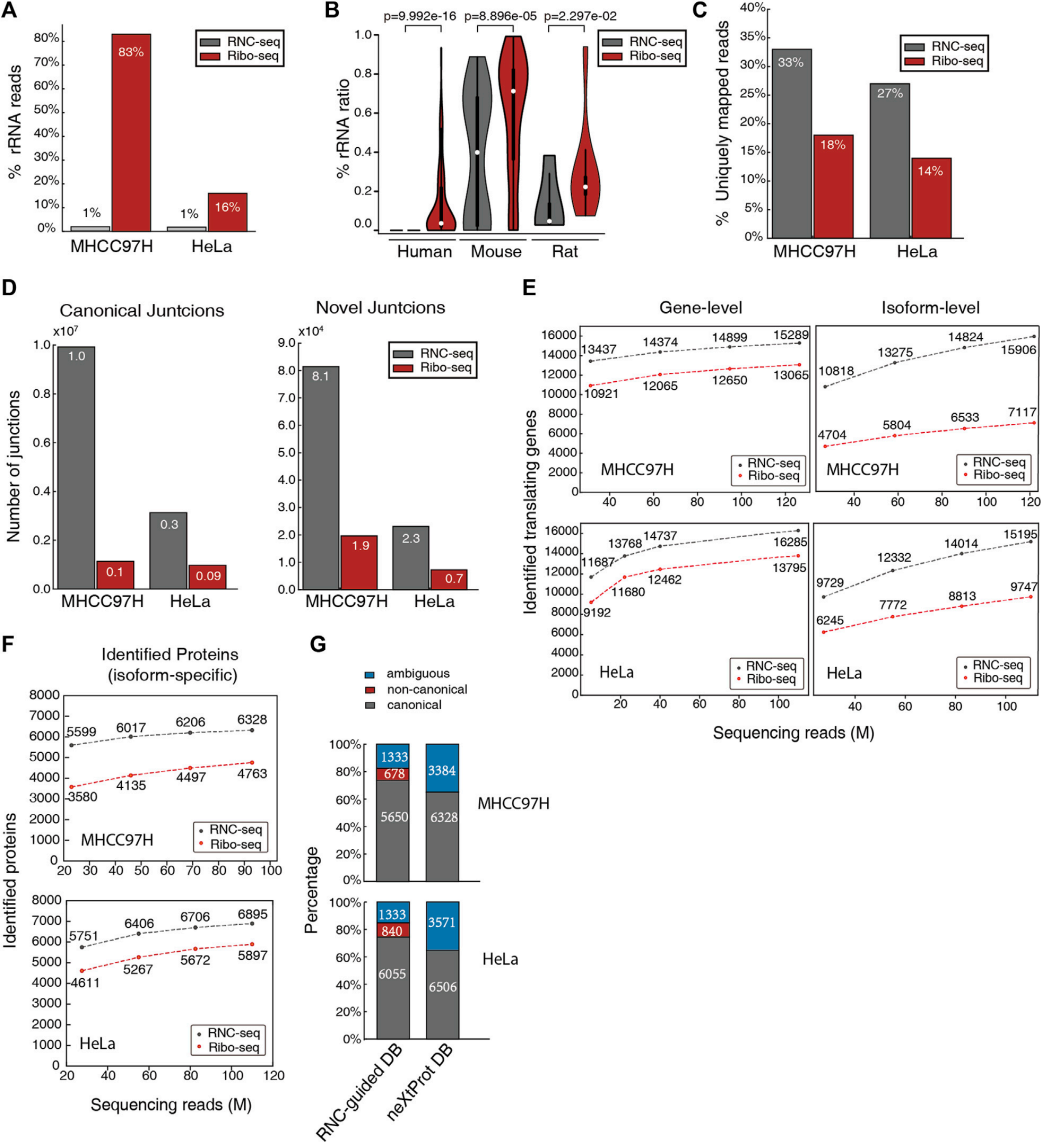
RNC-seq’s advantages over Ribo-seq in detecting splicing isoforms with higher efficiency. **(A)** Percentage of rRNA reads in RNC-seq and Ribo-seq datasets of MHCC97H and HeLa cells. **(B)** Percentage of ribosomal RNA reads in RNC-seq and Ribo-seq datasets in human, mouse, and rat. The *p*-values were obtained from Kolmogorov-Smirnov test. **(C)** Percentage of uniquely mapping reads (uni-mapped reads/non-rRNA reads) in RNC-seq and Ribo-seq. **(D)** Number of identified canonical junctions (left) and novel junctions (right) using same number of non-rRNA reads (25M). **(E)** Number of identified genes and isoforms supported by uniquely mapped reads under the same throughput of raw reads. **(F)** Number of protein isoforms identified by using RNC-seq and Ribo-seq guided protein database. **(G)** Proportion of identified distinct proteins by RNC-seq guided database and neXtProt. Ambiguous: these proteins share all their peptides with other proteins, and thus cannot be unambiguously identified.

Both RNC-seq and Ribo-seq need to extract ribosome fraction. In human cells, rRNAs account for 80–85% of the total RNA, while coding mRNAs account for only 1–5% (Zhao et al., 2018). The RNC-seq selects mRNA using poly-dT oligos, which effectively avoid sequencing rRNA. In contrast, the ribosomal footprints in Ribo-seq lacks polyA tail. The rRNA can only be removed by hybridization, which is trickier and often inadequate. For example, in MHCC97H and HeLa cells, our RNC-seq datasets contained only ∼1% rRNA reads, while Ribo-seq datasets contained 16–83% rRNA reads (Figure 1A). We analyzed 775 RNC-seq and Ribo-seq datasets in the TranslatomeDB (Liu et al., 2018). The Ribo-seq datasets contained much higher fraction of rRNA reads than RNC-seq (Figure 1B). This remarkably decreased the effective mRNA reads, which undermined the AS detection efficiency of Ribo-seq.

When detecting known splice variants, it is efficient to map short sequencing reads to RNA reference sequences. The uniquely mapped reads represent the specific splice variants. When normalized against the non-rRNA read number, the RNC-seq datasets (100 nt read length) yielded approximately doubled uniquely mapped reads than the Ribo-seq datasets (∼28 nt reads) (Figure 1C). This suggests that RNC-seq can improve the efficiency of AS isoforms identification not only by the better enrichment strategies, but also by the longer read lengths, since longer reads are more likely to span across the specific splice junctions and specific exons.

In addition, longer reads also provides more information for the algorithms to identify the reads across junctions more accurately, thereby facilitates the discovery of splice junctions including novel splice events (the splice events which are not annotated in the database). As a validation, we used STAR algorithm to map the rRNA-filtered clean reads to human genome GRCh38. The RNC-seq identified 3.2–8.7 × more canonical junctions and 3.5 × more novel junctions than Ribo-seq (Figure 1D).

We then evaluated the number of expressed genes and isoforms identified by RNC-seq and Ribo-seq. With the increasing number of raw reads, both RNC-seq and Ribo-seq can identify more expressed genes and isoforms (Figure 1E). However, using the same throughput of raw reads, RNC-seq identified 17–27% more translating genes and 1.6–2.3 × more known splice isoforms than Ribo-seq. For example, RNC-seq identified 15,906 isoforms by isoform-specific and uniquely mapped reads in MHCC97H cell line, while Ribo-seq identified only 7,117 isoforms. This trend is also valid when considering the same number of non-rRNA reads (Supplementary Figure S1).

Using the identified translating isoforms (with isoform-specific reads) to build protein databases to identify proteins in mass spectrometry datasets, the RNC-seq database identifies 17–56% more protein than Ribo-seq, following the criteria of HPP Guideline 3.0 (Figure 1F). Compared to the standard neXtProt (with isoforms) database, more than 75% of the identified isoforms were canonical ones, and detected 678–840 non-canonical proteins which are not included in neXtProt database (Figure 1G). Besides these uniquely identified proteins, only 1,333 proteins were ambiguously identified because all their identified peptides were shared with other proteins. In contrast, 3,384–3,571 proteins were ambiguously identified using neXtProt database (Figure 1G). This indicated that the RNC-seq-guided database provided more concise identifications.

We then demonstrated the total number of identified proteins by using the identified translating genes (with gene-specific reads) and translating isoforms (with isoform-specific reads) to guide protein identification. For example, by using RNC-guided database (constructed by 132M reads of MHCC97H and 110M reads of HeLa, respectively) we identified 10,887 and 11,308 proteins (Supplementary Figure S2), while only 6,328 and 6,506 proteins identified by using the neXtProt database. This result demonstrated that the RNC-seq-guided database strategy significantly optimized the protein identification efficiency.

In sum, RNC-seq has a distinct advantage in detecting translating AS isoforms and novel protein isoforms.

### The Translation Potential of Identified Non-coding RNAs Between RNC-Seq and Ribo-Seq

**FIGURE 2:**
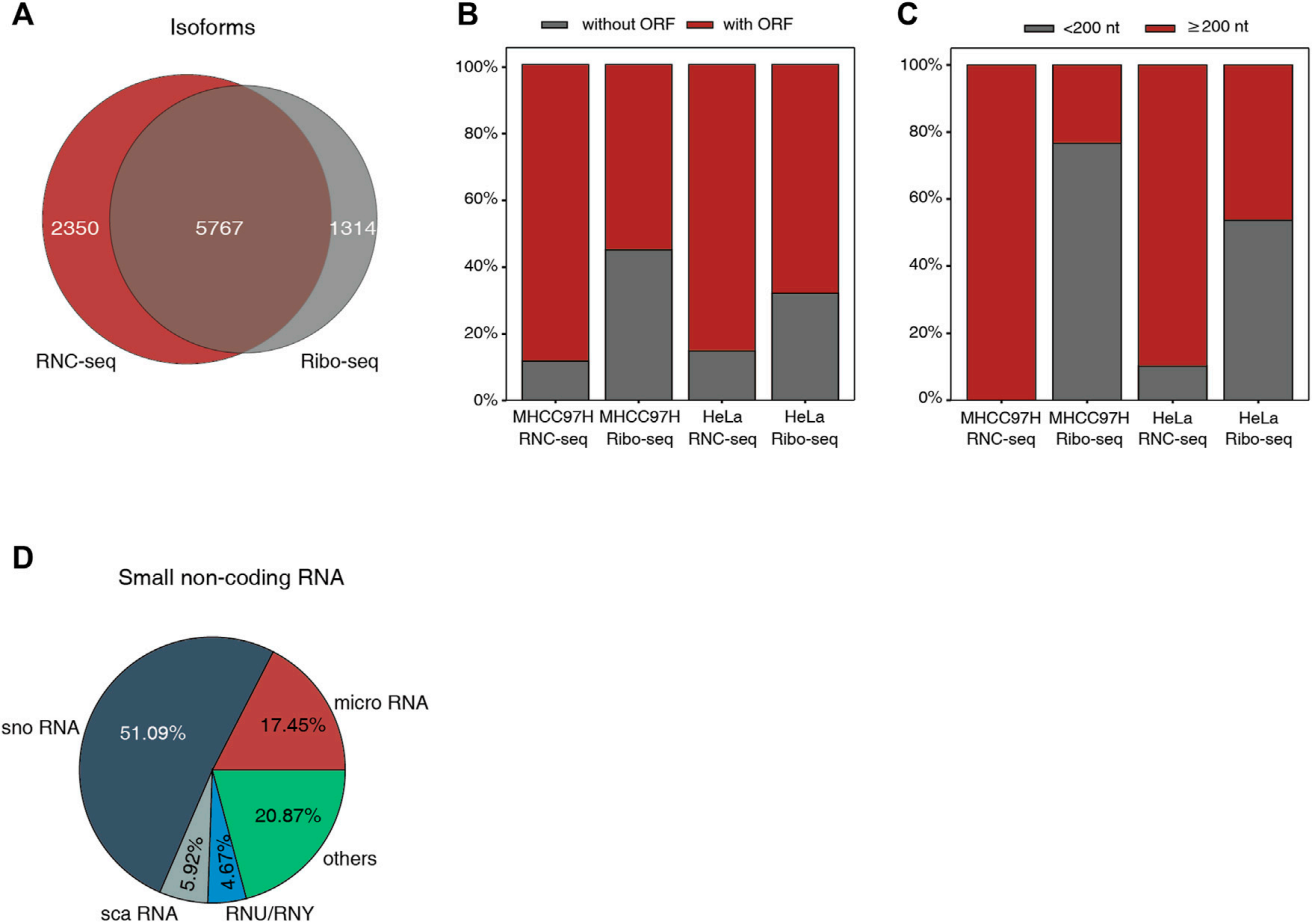
The difference of identified isoforms between RNC-seq and Ribo-seq. **(A)** Venn diagram of isoforms identified by RNC-seq and Ribo-seq using 8 million uniquely mapped reads, respectively. **(B)** Distribution of identified ncRNA with and without ORFs. **(C)** Length distribution of the ncRNA without canonical ORF. **(D)** Types of small ncRNA identified by Ribo-seq of MHCC97H.

It is known that many “ncRNAs” can be translated into proteins in canonical way. We evaluated the translating potential of these ncRNAs identified by RNC-seq and Ribo-seq under the same number of uniquely mapping reads (Figure 2A). In independently identification of RNC-seq, about 86% of the translating “ncRNAs” contain canonical AUG-started open reading frames (ORFs) of at least 50 aa in length, while in Ribo-seq, 55–68% of the translating “ncRNAs” contains canonical ORFs (Figure 2B). This raised a doubt whether such non-canonical “new proteins” identified by Ribo-seq were real. Indeed, 54–77% of the noncoding isoforms without canonical ORF identified by Ribo-seq were classical small noncoding RNAs (Figure 2C), mainly snoRNAs, which are unable to encode proteins ≥ 50 aa (Figure 2D). Previous studies have shown that ribosomes interact extensively with snoRNAs, such as ribosome biogenesis (Reichow et al., 2007), and the interaction of 80S ribosomes and pre-mRNA with snoRNAs induces degradation to generate mature mRNA and functional snoRNAs. Therefore, snoRNAs can cross the sucrose cushion with ribosomes during ultracentrifuging, which would lead to the detection of snoRNAs in final sequencing data. Due to the digestion of RNase, it is difficult for Ribo-seq to exclude the non-RFPs of small ncRNAs and degraded fragments (Smith and Steitz, 1998; Guttman et al., 2013).

### Direct Full-Length RNC Sequencing Reveals Isoform Complexity

It is difficult to determine exon arrangement solely by short reads. Single-molecule, long-read sequencing techniques (Iso-seq), such as PacBio or Nanopore, can solve this problem by sequencing the entire mRNA molecule (Rhoads and Au, 2015; Byrne et al., 2017). This provides a more detailed picture of the transcriptome and a powerful tool to detect novel AS isoforms. Using these techniques on RNC-seq, we can sequence the entire ribosome-bound mRNA to accurately determine the translating AS isoforms, as different AS isoforms are translated in different efficiency (Wang et al., 2013).

**FIGURE 3:**
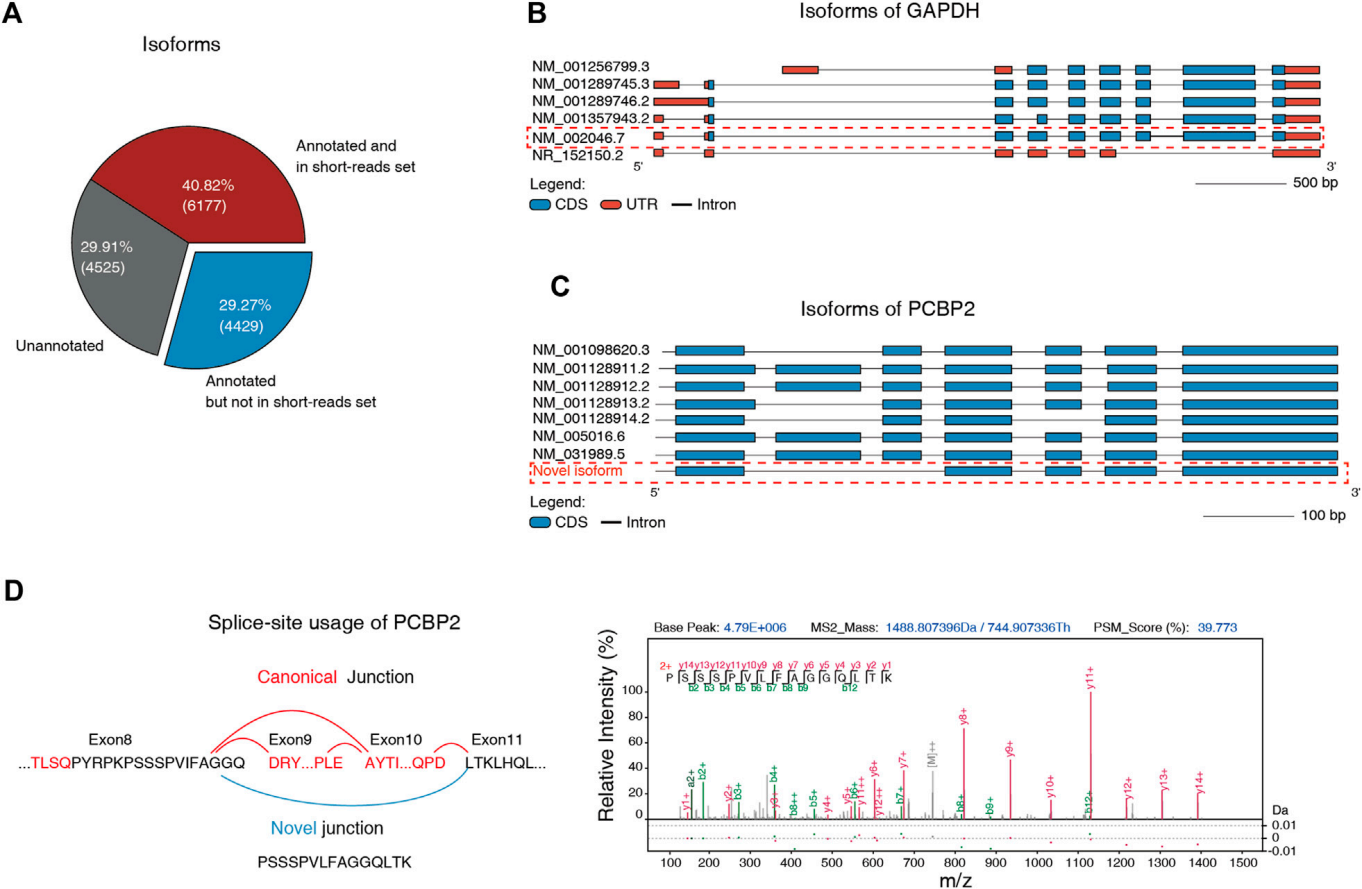
Single-molecule full-length RNC-seq improved the efficiency of isoforms identification. **(A)** Three types of identified isoforms by Nanopore RNC-seq. “Annotated” means that the isoform is included in NCBI human Refseq database. “in short-read set” means that the isoform can be identified by short-read RNC-seq. **(B)** All isoforms of GAPDH. The marked isoform was identified by full-length RNC-seq but not by short-read RNC-seq. **(C)** All isoforms of PCBP2. Nanopore full-length RNC-seq identified a novel isoform that was absent in Refseq and neXtProt databases. **(D)** The MS-detected specific junction peptide (PSSSPVLFAGGQLTK) that supported the novel junction of PCBP2.

We performed the direct full-length RNC-seq on MHCC97H using Nanopore MinION sequencer. Due to the high error rate (∼8.47%) of the single-molecule Nanopore sequencer, we corrected the sequences by more accurate short read RNC-seq data (error rate ∼0.61%) corresponding to ensure the correction of novel isoforms identification. After filtering out novel singletons (Sessegolo et al., 2019), the full-length RNC-seq identified in total 15,131 unique AS isoforms. Among these isoforms, 4,525 (29.91%) were absent in NCBI Refseq mRNA reference database, and 4,429 (29.27%) were annotated in RefSeq database but cannot be identified by the short-read RNC-seq (Figure 3A). For example, we identified an isoform NM_002046 of gene GAPDH with the unique combination of exons by long reads of Nanopore sequencing, but would be missing in short-read sequencing because it has no unique splice junction compared to other splice isoforms (Figure 3B).

Next, we detected novel isoforms at protein level. We built a protein database by 3-frame-translation of all detected isoforms. This database included 18,200 protein sequences was used for mass spectrometry-based proteome identification. This database is much smaller than the neXtProt database with isoforms (41,653 entries). Therefore, the sensitivity was expected to be better. We detected 6,766 isoforms with at least 1 isoform-unique peptide ≥ 9 aa.

After filtering out the proteins whose unique peptides shared sequences with canonical proteins in neXtProt (with isoforms), we finally identified 50 novel protein isoforms which were not included in RefSeq and neXtProt reference with FDR < 0.01. When we controlled the FDR of protein-level and peptide-level to 0.001, there are still 43 novel isoforms could be identified with stricter quality control (Supplementary Table S1, S2, Supplementary Figure S3).

For example, we detected a novel isoform of PCBP2 gene with a unique peptide (Figures 3C,D). Compared with other annotated isoforms, this isoform alternative spans across the exons 8 and 11, and skips exons 9 and 10. These results indicates that the long-read RNC sequencing is efficient to reveal novel human protein isoforms.

## Discussion

Protein AS isoform is of great importance in proteome studies and has not been specifically and thoroughly investigated in the context of HPP because of the difficulties of detecting isoform-specific peptides in large-scale MS data (see the Introduction section). Previously, the cell line-specific protein sequence database based on transcriptome. However, it seems that the efficiency of such approaches was not satisfying. The Human Proteoform Atlas database collected 3,055 protein isoforms in human proteome, which was identified in 2011–2014 from all studies they could collect (Hollas et al., 2022). A recent study on 19 cell types from human blood reconstructed 95,979 transcripts from transcriptome sequencing, but identified only in total 4,344 proteoforms using such database in 19 cell types (Melani et al., 2022). In contrast, we identified 6,766 isoforms with unique peptides from one HeLa cell line using our RNC-seq-guided database strategy, much more efficient than the transcriptome-guided database strategy. One possible reason is that the transcriptome-based protein reference database contained too many false entries (i.e., the protein sequences that were actually not translated into proteins): the 3-frame-translation of RNA generates many false entries, and many mRNAs were not translated, especially some AS transcripts (Wang et al., 2013). Excessive number of false entries largely expands the database and decrease the sensitivity and confidence of database search under the widely-used Target-Decoy scheme (Khatun et al., 2013). RNC-seq detects only the translating RNAs and thus creates a minimal protein database, which would solve the problem. At steady state, the translating RNA corresponds to proteins (Zhong et al., 2014). At non-steady state, there might be proteins which were not being synthesized but not fully degraded. Using RNC-seq-guided database may lead to false negatives, but it can still provide confident identifications of those proteins which are being synthesized.

Unique peptides are required to evidence the existence of a proteoform. However, 11,757 proteins in neXtProt database were predicted to have no unique peptides that allows isoform identification (Jeong et al., 2018). The major reason is that most of these isoforms do not have specific junction or exons that can be distinguished from other isoforms. They are unique just due to the unique combination of exons. We can distinguish such isoforms by single-molecule full-length RNC-seq, but the short peptides prevent unique identification at protein level. In such cases, RNC-seq provides indirect evidence of these isoforms. Protein evidence need advances in other experimental strategies, for example, top-down proteome methods.

Long-read RNC-seq also found 4,525 isoforms that were not included in RefSeq databases, and we identified 50 proteins out of these isoforms at protein level. This suggests a hidden proteome from these newly discovered isoforms. It should be noted that our Nanopore RNC-seq yielded only 1.43M reads. When elevating the throughput, considerably more “new” isoforms are expected to be discovered.

In sum, RNC-seq is an efficient and economical way (compared to Ribo-seq) to investigate the proteome of AS variants, and facilitates the functional studies of these isoforms.

## Materials and Methods

### Cell Lines and Reagents

The human hepatocellular carcinoma MHCC97H cell line was kindly provided by Professor Yinkun Liu, Fudan University. MHCC97H cells were cultured in the DMEM (Life Technologies, Carlsbad, CA, United States) medium supplemented with 10% fetal bovine serum (Life Technologies, Carlsbad, CA, United States), 1% penicillin/streptomycin (Life Technologies, Carlsbad, CA, United States) and 10 μg/ml ciprofloxacin, and both of cells were detected free of *mycoplasma* during maintenance and upon experiments.

### Ribosome-Nascent Chain Complex Isolation

The method of ribosome-nascent chain complex (RNC) isolation was generated as described before (Wang et al., 2013). In brief, MHCC97H cells were pre-treated with 100 μg/ml cycloheximide (Acmec, Shanghai, China) for 10 min at 37°C, followed by 5 ml pre-colded PBS (Beyotime, Shanghai, China) washes twice and lysis for 30 min on ice by 2 ml pre-cooled human cell lysis buffer (20 mM Tris-HCl, 5 mM MgCl_2_, 150 mM KCl, 1 mM DTT, 100 μg/ml cycloheximide, 25 units/mL Turbo DNase I, 1% Triton X-100). Cell lysates were clarified by centrifuge at 17000 × g at 4°C for 15 min, supernatants were transferred on the surface of 14.5 ml sucrose cushion (30% sucrose, 20 mM Tris-HCl, 5 mM MgCl_2_, 150 mM KCl, 1 mM DTT, 100 μg/ml cycloheximide). RNCs were purified by ultra-centrifugation in a Type 70Ti rotor (Beckman Coulter, Brea, CA, United States) at 185,000 × g for 5 h at 4°C.

### RNA Extraction and mRNA Sequencing Library Construction

Total MHCC97H RNC-RNA were isolated using TRIzol reagent (Invitrogen, Carlsbad, CA, United States). 1 μg of total RNC-RNA were subjected for library construction. Briefly, PolyA + mRNAs were isolated using VAHTS mRNA Capture Beads (Vazyme, Jiangsu, Nanjing, China). The sequencing library was constructed by MGIEasy RNA library Preparation kit (MGItech, Guangdong, Shenzhen, China) following the manufacturer’s instructions. Libraries were sequenced in a BGI-Seq 500 (MGItech, Guangdong, Shenzhen, China) sequencer at SE100 mode. The raw data of RNA sequencing can be found below: NCBI SRA Bio Project, accession no: GSE198624.

### Ribosome Profiling

The method of ribosome profiling was generated as described before (Ingolia et al., 2012) with some modification. In brief, MHCC97H cells were pre-treated with 100 μg/ml cycloheximide for 10 min at 37°C, and then washed twice using 5 ml pre-cooled PBS. The cells were lyzed for 30 min on ice in 2 ml pre-colded human cell lysis buffer. Cell lysates were clarified by centrifuge at 17000 × g at 4°C for 15 min. Purified lysates were treated with 7 units RNase I (Thermo Fisher, Waltham, MA, United States) per OD260. The RNase digestion was performed at 4°C with gentle mixing for 60 min, and then stopped by adding 10 μL of SUPERase-in RNase inhibitor (Thermo Fisher, Waltham, MA, United States). Monosomes were pelleted using ultra-centrifugation in a Type 70Ti rotor at 185,000 × g for 3 h at 4°C.

RNA was isolated using TRIzol reagent. RNA less than 200 nt was isolated using Zymo RNA clean and concentrator kit (Zymo Research, Orange, CA, United States). RNAs with the length of 17–200 nt were loaded on a 15% (w/v) Urea PAGE and resolved by gel electrophoresis. Ribosome footprints were purified by gel recovery according to the Zymo small-RNA PAGE Recovery kit (Zymo Research, Orange, CA, United States). The 3′ dephosphorylation reaction was performed using T4 PNK (New England Biolab, Hitchin, Hertfordshire, United Kingdom) without ATP. The 5’ phosphorylation reaction was performed using T4 PNK with 1 mM ATP. RNA was precipitated by adding 39 µL of nuclease free water, 1.0 µL of GlycoBlue coprecipitant (Invitrogen, Carlsbad, CA, United States) and 10 µL of 3 M sodium acetate (pH5.5), 150 µL isopropanol, at −80°C overnight. Ribosome footprints sequencing library was constructed by using MGIEasy Small RNA library preparation kit. Libraries were sequenced in a BGI-Seq 500 sequencer at SE50 mode. The raw data of RNA sequencing can be found below: NCBI SRA Bio Project, accession no: GSE198624.

### Protein Extraction and Protein Digestion

MHCC97H Cell line was cultured to 80–90% coverage and treated with 1% SDS lysis buffer (Beyotime, Shanghai, China) and the protein concentration was measured using a BCA kit (Thermo Fisher, Waltham, MA, United States).

The protein digestion was performed by filter-aided sample preparation (FASP) (Wisniewski et al., 2009) as we previously described (Lu et al., 2019). In brief, firstly, 1 mg protein samples were reduced and alkylated using dithiothreitol solution (DTT) (Solarbio, Beijing, China) and iodoacetamide solution (IAA) (I6125, Merck, Kenilworth, NJ, United States) at a final concentration of 4 M urea (8 M urea in 0.1 M Tris-HCl, pH 8.5). Secondly, all the solution was transferred to a 10KD ultrafiltration tube (Merck, Kenilworth, NJ, United States) and centrifuged at 12000 g, and then washed 3 times with 50 mM TEAB (Thermo Fisher, Waltham, MA, United States). Thirdly, trypsin (V5280, Promega, Madison, WI, United States) was added in a ratio of 1:40, and incubated overnight at 37°C. The peptides were collected into low-binding collection tube (Thermo Scientific™, Waltham, MA, United States) and then measured the concentration using Pierce Quantitative Fluorometric Peptide Assay (Thermo Scientific™, Waltham, MA, United States). Finally, the peptides were freeze-dried and stored at −80°C.

### Data-Dependent Acquisition Mass Spectrometry

Firstly, the total peptides were fractionated using high-pH reverse-phase liquid chromatography (RPLC). Specifically, 600 µg peptide was re-dissolved in 100 µL buffer A (2% (v/v) ACN, pH 10), and loaded onto the C18 column (4.6 × 250 mm, C18, 3 μm, 186003581, Waters, Milford, MA, United States). The elution gradient was buffer B (98% ACN, pH 10; flow rate, 800 μL/min) for 65 min, the elution gradient was as follows: 5% B, 0 min; 5% B, 6 min; 37% B, 28 min; 46% B, 45 min; 90% B, 46 min; 90% B, 54 min; 95% B, 55 min; 95% B, 65 min. The eluted peptides were collected every minute from the 6th minute until the 54th minute, and then the front, middle and rear fractions were combined into 16 fractions with equal peak area, finally, the fractionated peptides were freeze-dried.

Secondly, the 16 fractionated peptides were redissolved in 0.5% (V/V) trifluoroacetic acid (TFA) (Macklin, Shanghai, China) and were desalted using a Mono tip C18 columns (Shimadzu, Kyoto, Japan) following the manufacturer’s instructions and freeze-dried.

Finally, the 16 fractions of the desalted peptide were re-dissolved in 0.1% (V/V) formic acid (FA) (Thermo Scientific™, Waltham, MA, United States) and then preformed DDA analysis by using Orbitrap Fusion Lumos mass spectrometer equipped with EASY-nLC 1200 system (Thermo Scientific™, Waltham, MA, United States). 2 µg of each fractions peptides were loaded on a nano trap column (C18, 150 μm × 20 mm, 1.9 μm, homemade), and then separated onto an analytical column (C18, 150 μm × 300 mm, 1.9 μm, homemade) using a 120 min linear gradient (solvent A: 98% H2O, 2% ACN, 0.1% FA; solvent B: 98% ACN, 2% H2O, 0.1% FA) at a flow rate of 600 nL/min. The detailed solvent gradient was as follows: 5–12% B, 28 min; 12–24% B, 58 min; 24–38% B, 25 min; 38–95% B, 1 min; 95% B, 8 min. The MS1 scan was acquired from 350 to 1500 m/z with a resolution of 120 k, the MS2 scans were performed at a resolution of 15 k with an isolation window of 1.6 m/z, the cycle time was set to 3s with a dynamic exclusion of 30s. All the MS raw data for DDA are publicly available in ProteomeXchange with identifier PXD032201.

### AS Event Calling

The HeLa Ribo-seq datasets (accession SRR3306589) (Park et al., 2016) and HeLa RNC-seq datasets (accession SRR6929904) (Li et al., 2018) were obtained from NCBI. We extract the ribosomal RNA sequences in the NCBI RefSeq-RNA database (downloaded on 6 December 2019) according to refFlat annotation (downloaded on 17 January 2020) as a human rRNA reference dataset. For full-length translating (RNC) mRNA-seq datasets, reads were mapped to rRNA reference sequences using FANSe3 (Zhang et al., 2021) (Release version 3.13) with the parameters–S12 –E4 in HeLa (50bp read lengths), and–S14 -E4 in MHCC97H (100bp read lengths). For Ribo-seq datasets, reads were mapped to rRNA reference sequences using FANSe3 with the parameters–S10 -E2 -U1. Reads that can be aligned to rRNA reference sequences will be considered rRNA reads and discarded. The filtered reads from each sample were mapped to the NCBI RefSeq-RNA database using FANSe3 with unique mode based on the same parameters as above. The UCSC refFlat annotation was used for isoform calling. Isoforms with uniquely-mapped read count≥10 were considered as detected.

### Splice Junction Analysis

RNC-seq and Ribo-seq were mapped to the GRCh38 no-alt analysis set (accession GCA_00001405.15) using STAR (2.5.0a) (Dobin et al., 2013) with the gtf option (GRCh38 full analysis set, accession GCA_000001405.15). The total number of splice junctions detected for each sample was taken from the log.final.out file printed by the aligner STAR (Dobin et al., 2013). We defined “Annotated (SJDB) Juntcion” as the identified canonical splice junctions, and “non-canonical junction” as potential novel junctions.

### Protein Isoforms Identification

The MHCC97H and HeLa custom protein isoforms databases were built by translating identified AS isoforms into protein sequences. neXtProt (release 2020-07-17) database was used as a negative control. pFind (version3.1.4) (Wang et al., 2007) was utilized to search protein isoforms in Hela (accession PXD004452) (Bekker-Jensen et al., 2017) and MHCC97H mass spectrometric datasets. The FDR threshold was set to 0.01 at both peptide level and protein level. The carbamidomethyl [C] was set as fixed modification, and oxidation [M] as variable modification during the search. The product ion tolerance was set as default parameters and precursor mass tolerance was set to 10 ppm. The missed cleavage was set to 2 for each peptide.

### Full-Length Single-Molecule RNC-Seq

Direct RNA Sequencing Kit (Oxford Nanopore Technologies plc, Oxford, United Kingdom) was used for full-length RNC-seq library preparation. The prepared library was load into a MinION flow cell (Oxford Nanopore Technologies plc, Oxford, United Kingdom) and sequenced on MinION device (Oxford Nanopore Technologies plc, Oxford, United Kingdom). Base-calling was performed with MinKNOW (V 3.6.5). Reads were aligned to GRCh38 no-alt analysis set (accession GCA_00001405.15) using minimap2 v2.7-r654 (Li, 2018) in spliced alignment mode with the command: minimap2 -ax splice -uf -k14 -secondary = no. FLAIR *correct* (v1.5) (Tang et al., 2020) was used to correct the splice-site boundaries of reads. All splice sites were assessed for validity by checking for support in genome annotation file (GRCh38 full analysis set, accession GCA_000001405.15). Splice junctions were extracted from matched RNC-seq data, and only the junctions supported by > 5 uniquely mapped short reads were considered valid. Incorrect splice sites were replaced with the nearest valid splice site within a 10-nt window. Isoforms were assembled and identified using FLAIR *collapse* (v1.5) (Tang et al., 2020) with the default settings. We filtered out the single exon transcripts to increase the confidence of the identification results. The raw data of RNA sequencing can be found below: NCBI SRA Bio Project, accession no: GSE198624.

## Data Availability

The datasets presented in this study can be found in online repositories. The names of the repository/repositories and accession number(s) can be found in the *Materials and Methods*.
