# Supplementary material for: Efficient Detection of the Alternative Spliced Human Proteome Using Translatome Sequencing
Source: Front Mol Biosci. 2022 Jun 2;9:895746. doi: 10.3389/fmolb.2022.895746 (PMC9201276; doi:10.3389/fmolb.2022.895746)

Supplementary Figure S3

Protein Name: MYRF_ORF_1
Peptide UUID: 0378fc19-fe38-4335-8d11-a4c5634d2ecc_MYRF_ORF_1

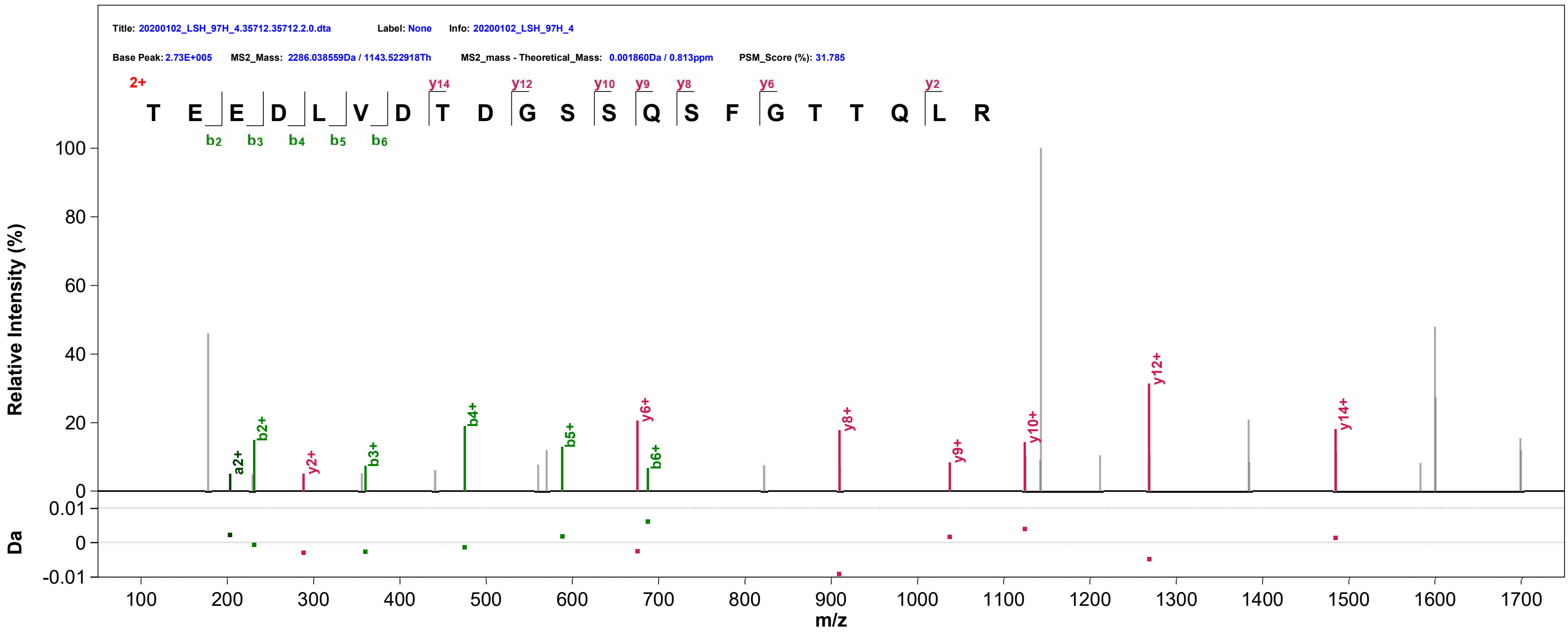

Protein Name: EIF3H_ORF_1
Peptide UUID: 0a63bd4a-082f-4e6a-a17e-b13f265019b1_EIF3H_ORF_1

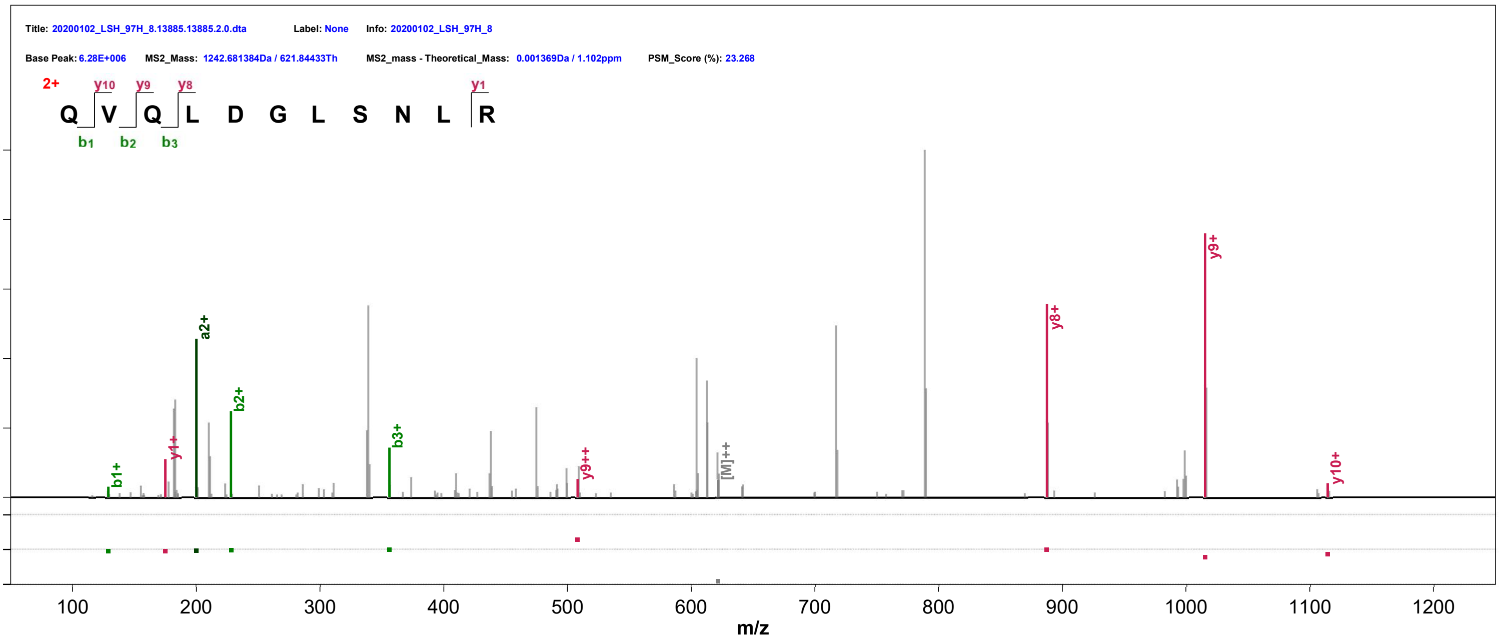

Protein Name: KIF21A_ORF_0
Peptide UUID: 142d5823-6bc6-449d-be03-20e2a76f3003_KIF21A_ORF_0_9

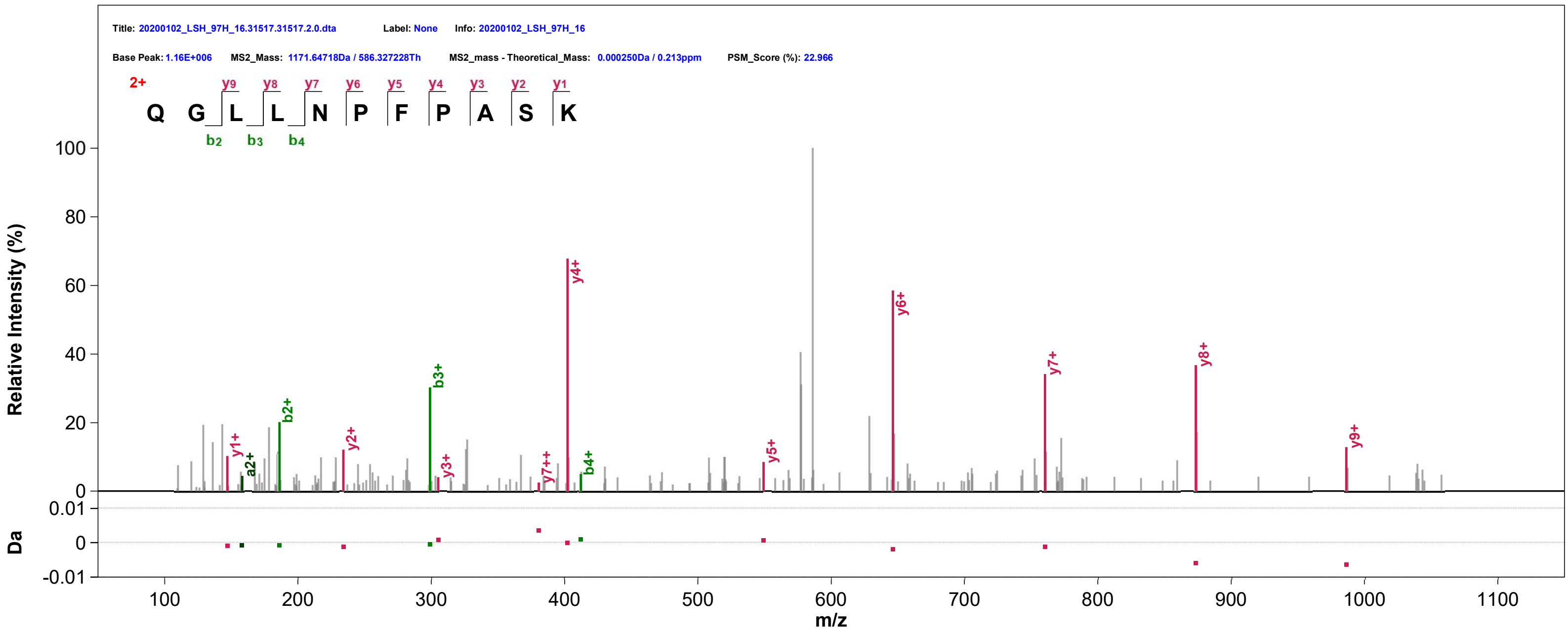

Protein Name: TJP1_ORF_2
Peptide UUID: 14de2e47-45c3-4c8b-b977-1083d5aee360_TJP1_ORF_2

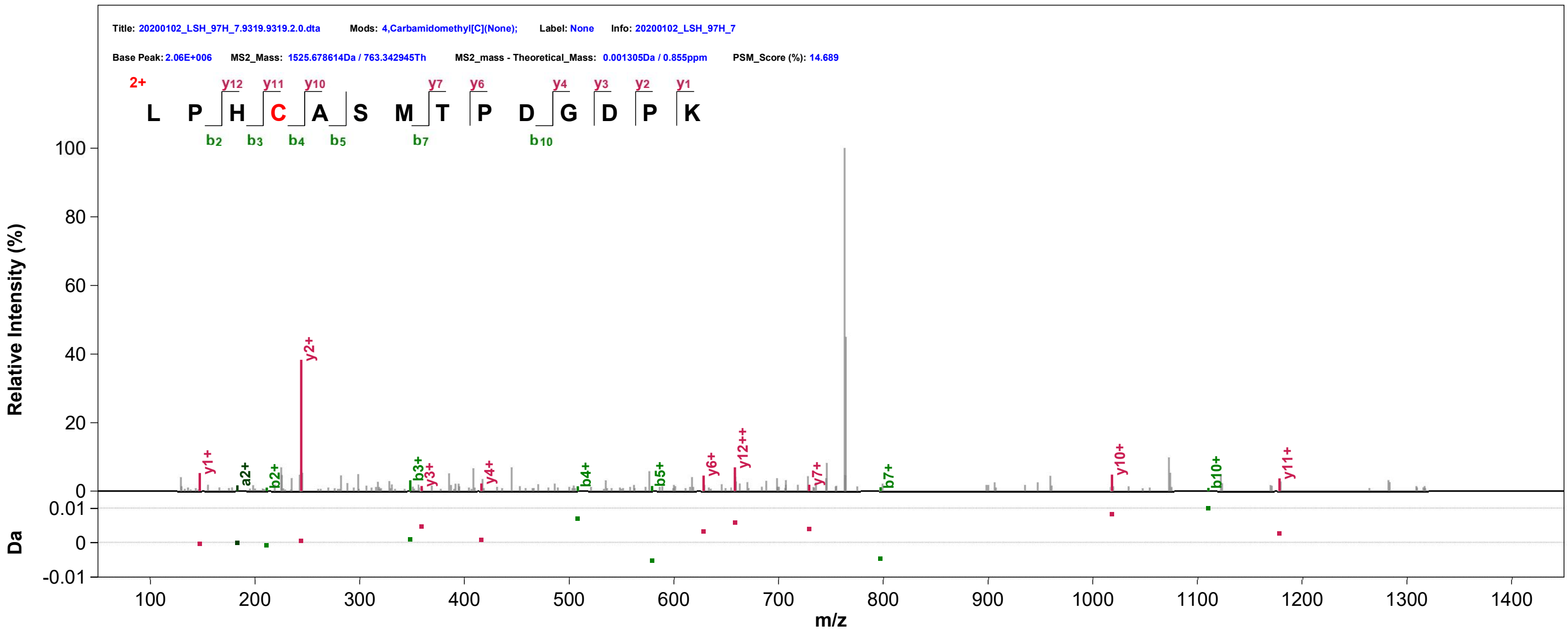

Protein Name: ARHGEF4_ORF_2
Peptide UUID: 230dce45-03d5-4d62-9040-b7c4224797f9_ARHGEF4_ORF_2_1

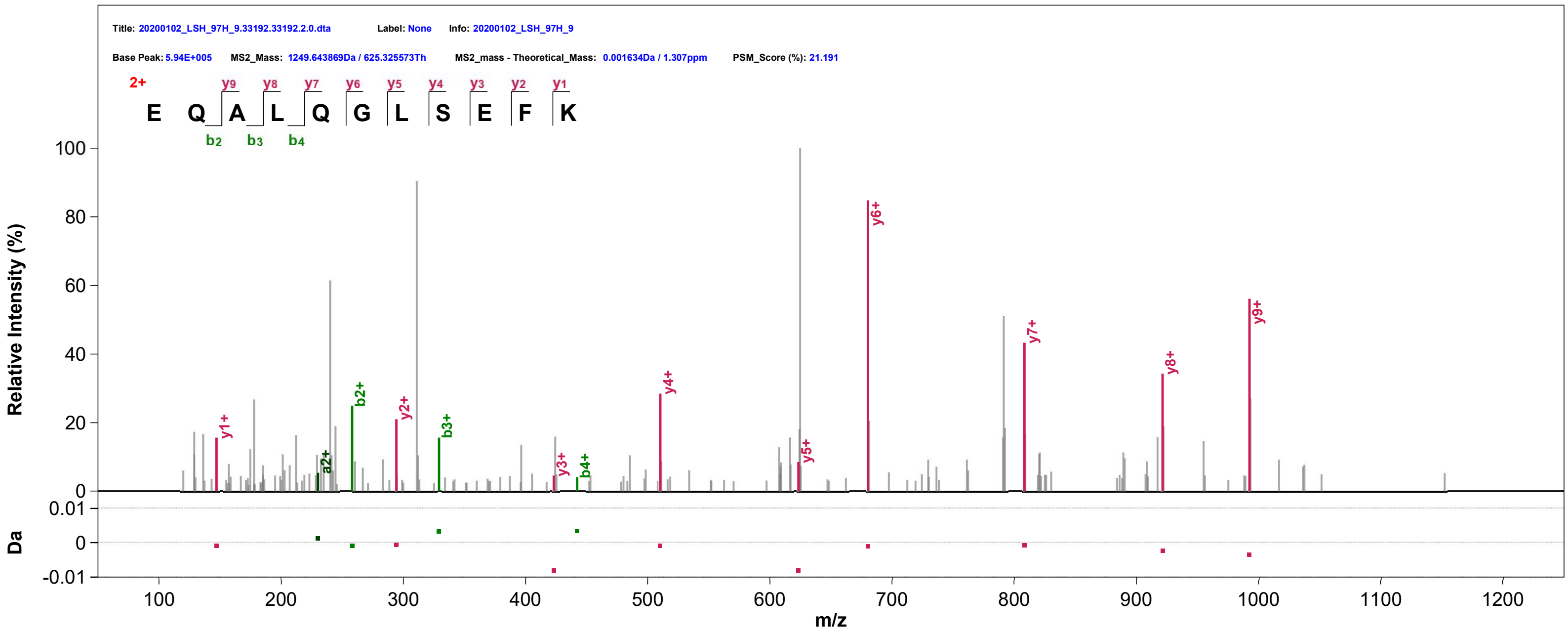

Protein Name: ARHGEF4_ORF_2
Peptide UUID: 230dce45-03d5-4d62-9040-b7c4224797f9_ARHGEF4_ORF_2_3

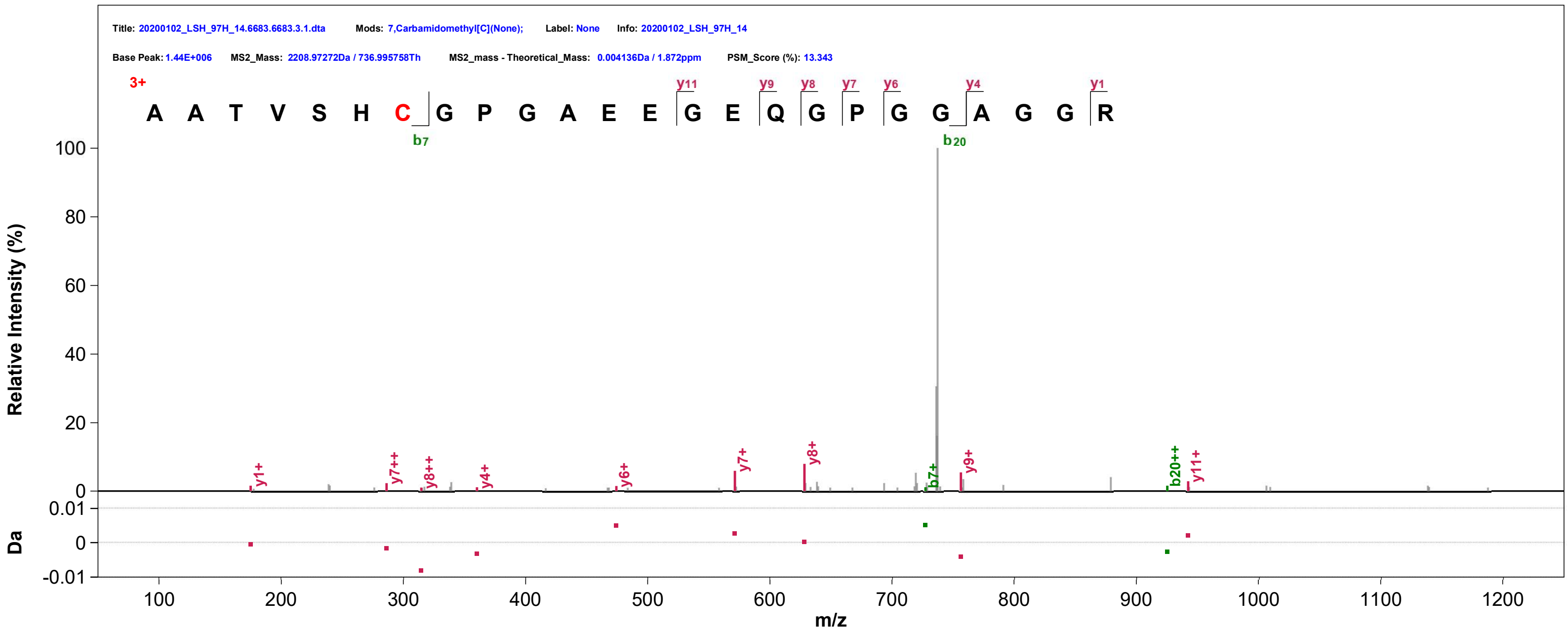

Protein Name: LGALS4_ORF_0
Peptide UUID: 2ad8b770-d5d0-4a96-9aca-3dcdf99631d5_LGALS4_ORF_0

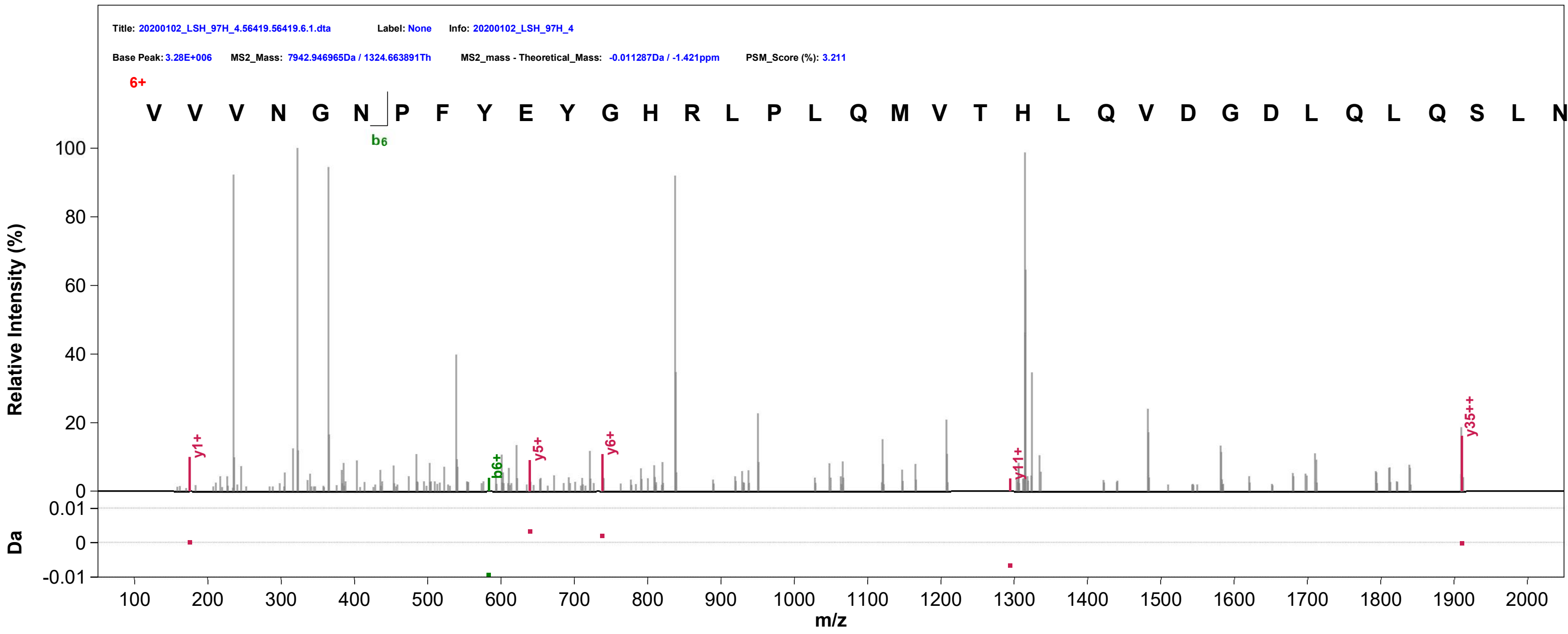

Protein Name: PHF10_ORF_0
Peptide UUID: 2db6834c-bdaf-464d-9f3c-acad02d60b74_PHF10_ORF_0_1

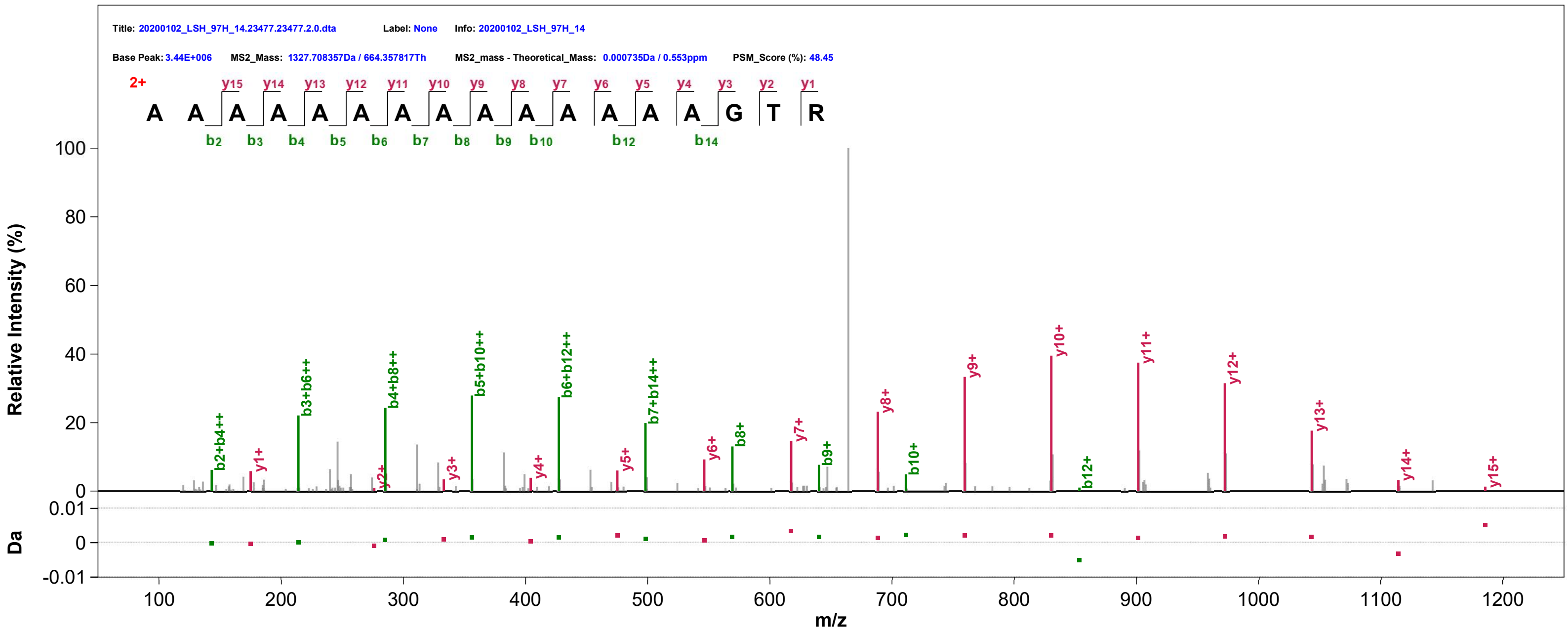

Protein Name: PHF10_ORF_0
Peptide UUID: 2db6834c-bdaf-464d-9f3c-acad02d60b74_PHF10_ORF_0_2

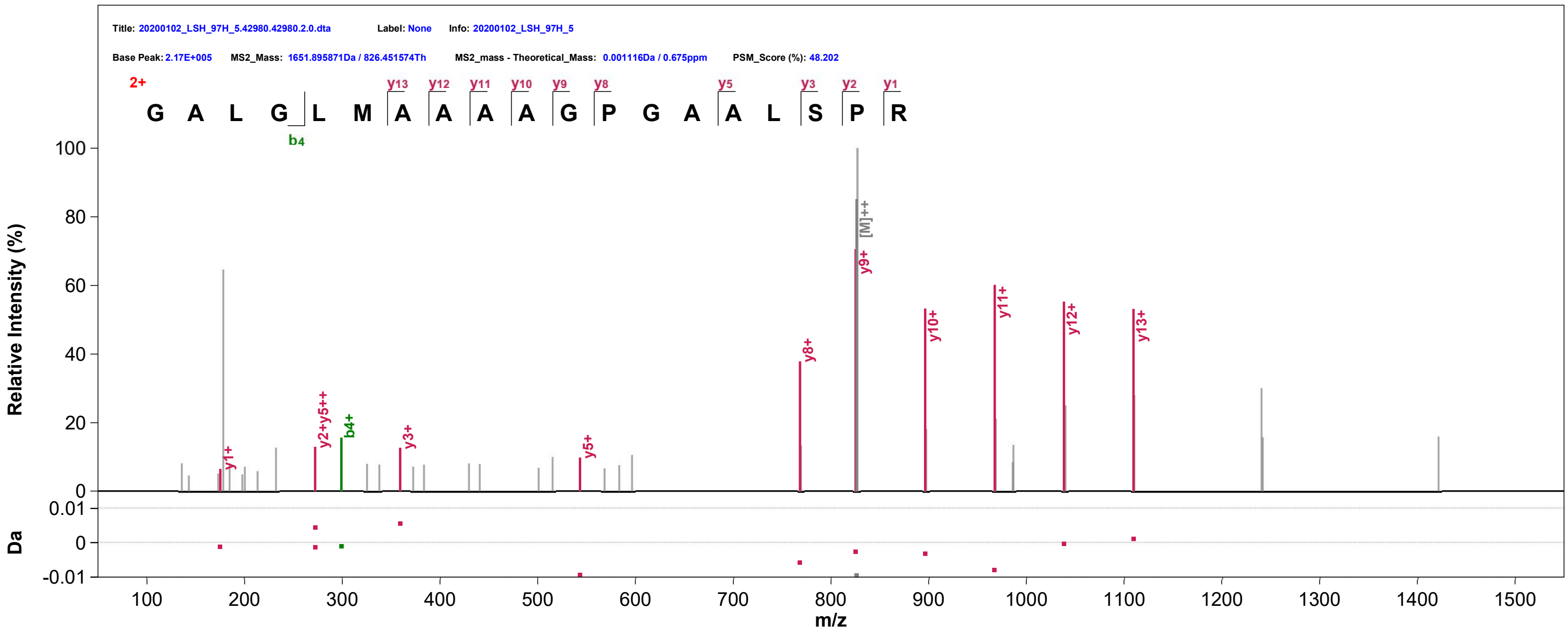

Protein Name: MPRIP_ORF_0
Peptide UUID: 488810e4-13ce-4650-9dfe-6057bc365e7b_MPRIP_ORF_0_1

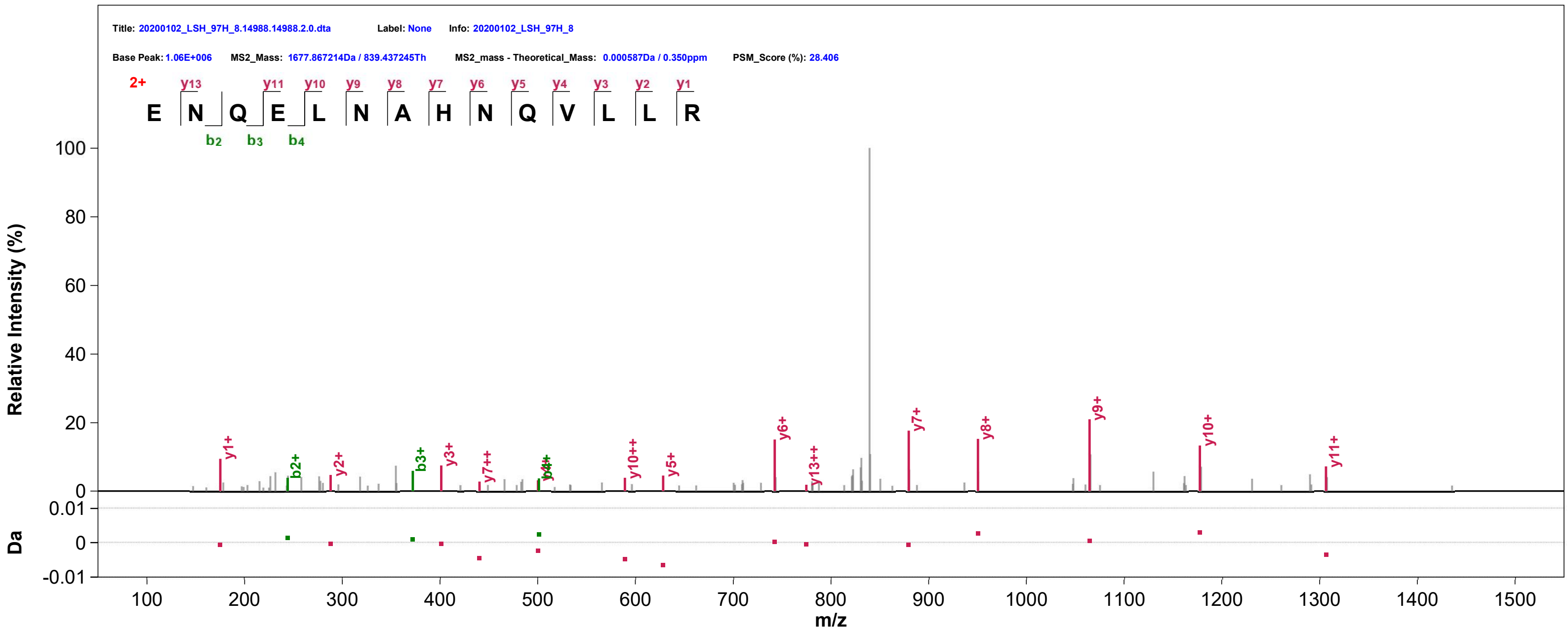

Protein Name: MPRIP_ORF_0
Peptide UUID: 488810e4-13ce-4650-9dfe-6057bc365e7b_MPRIP_ORF_0_2

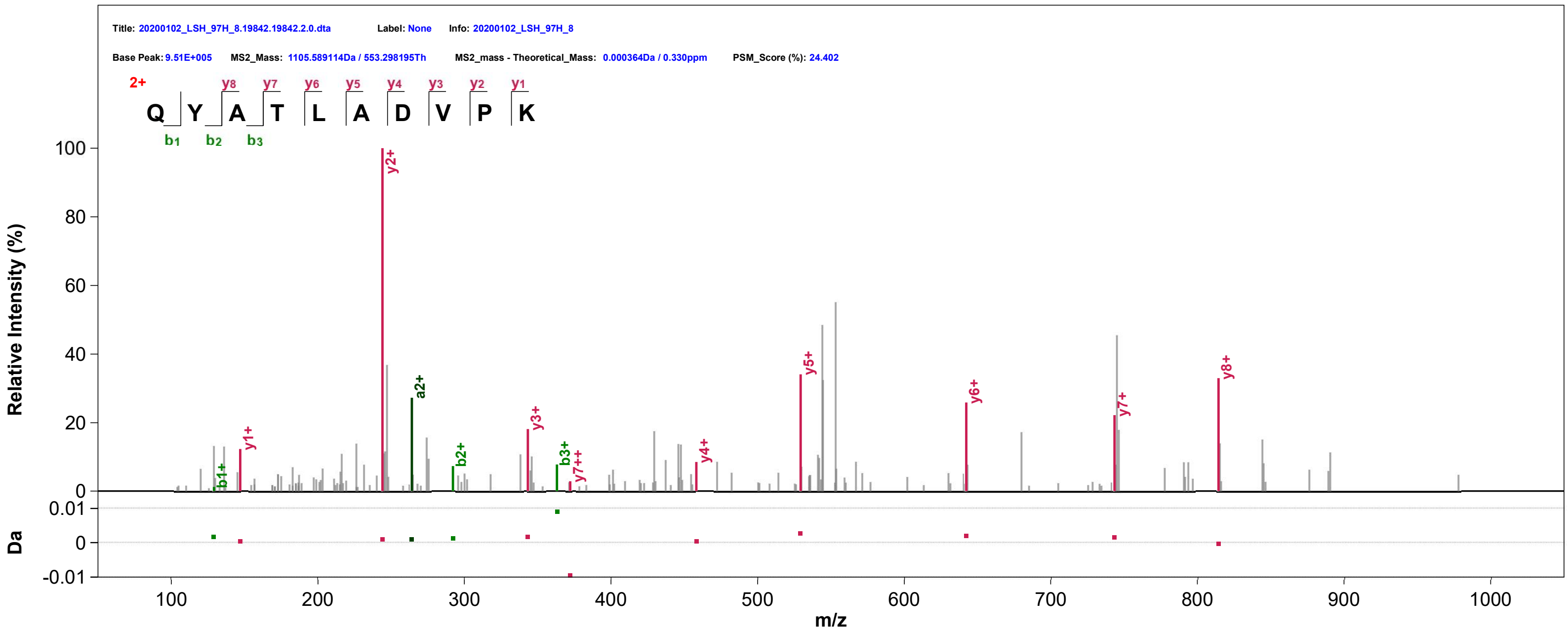

Protein Name: MPRIP_ORF_0
Peptide UUID: 488810e4-13ce-4650-9dfe-6057bc365e7b_MPRIP_ORF_0_3

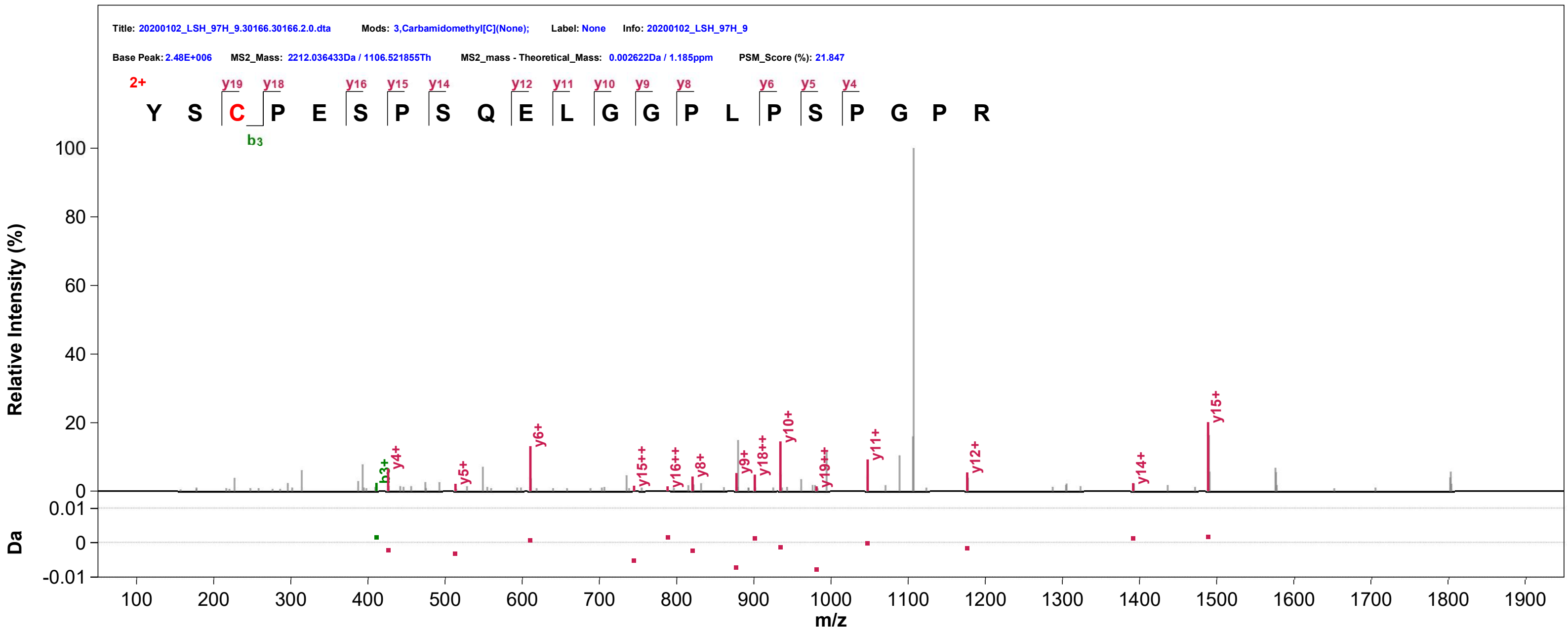

Protein Name: NCOR2_ORF_0
Peptide UUID: 502c1546-bc80-4b5b-8a1b-2721ce1a42b6_NCOR2_ORF_0_9

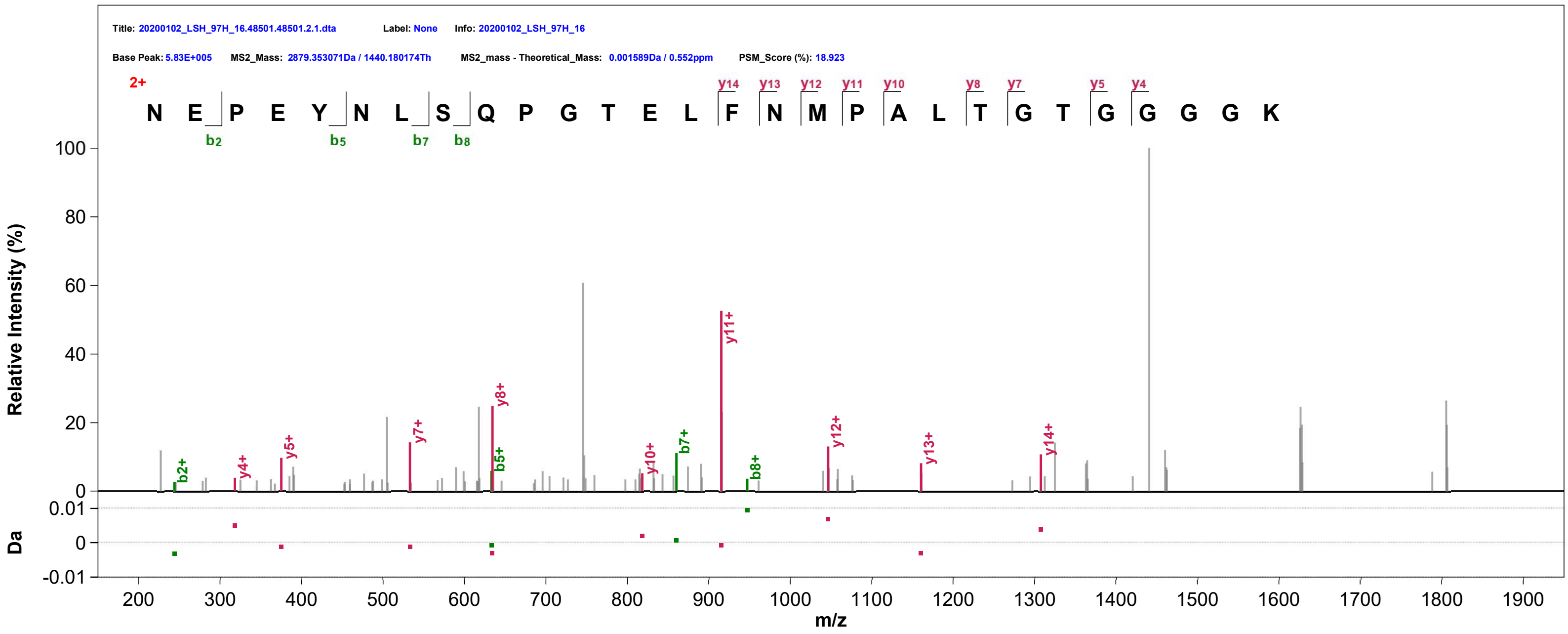

Protein Name: HLA-H_ORF_2
Peptide UUID: 50f5497b-53b0-44a5-96a9-86759cc493dd_HLA-H_ORF_2_1

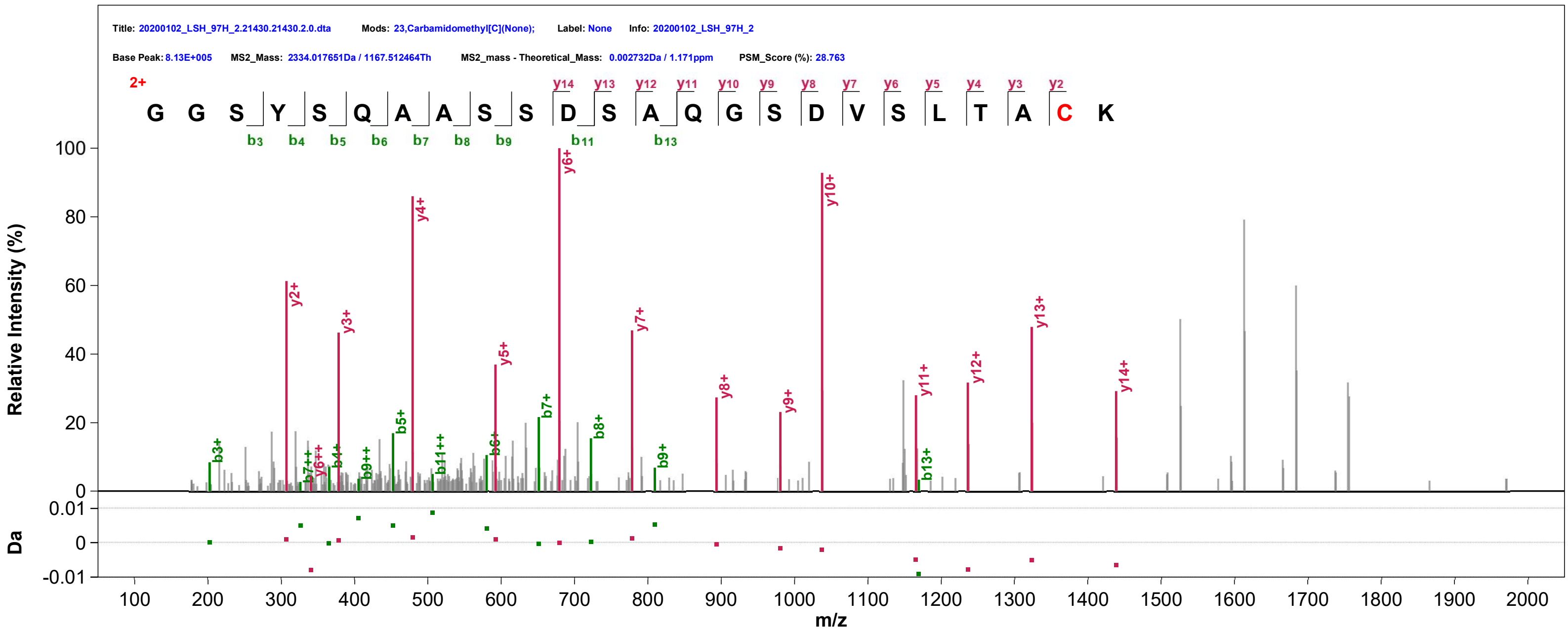

Protein Name: HLA-H_ORF_2
Peptide UUID: 50f5497b-53b0-44a5-96a9-86759cc493dd_HLA-H_ORF_2_2

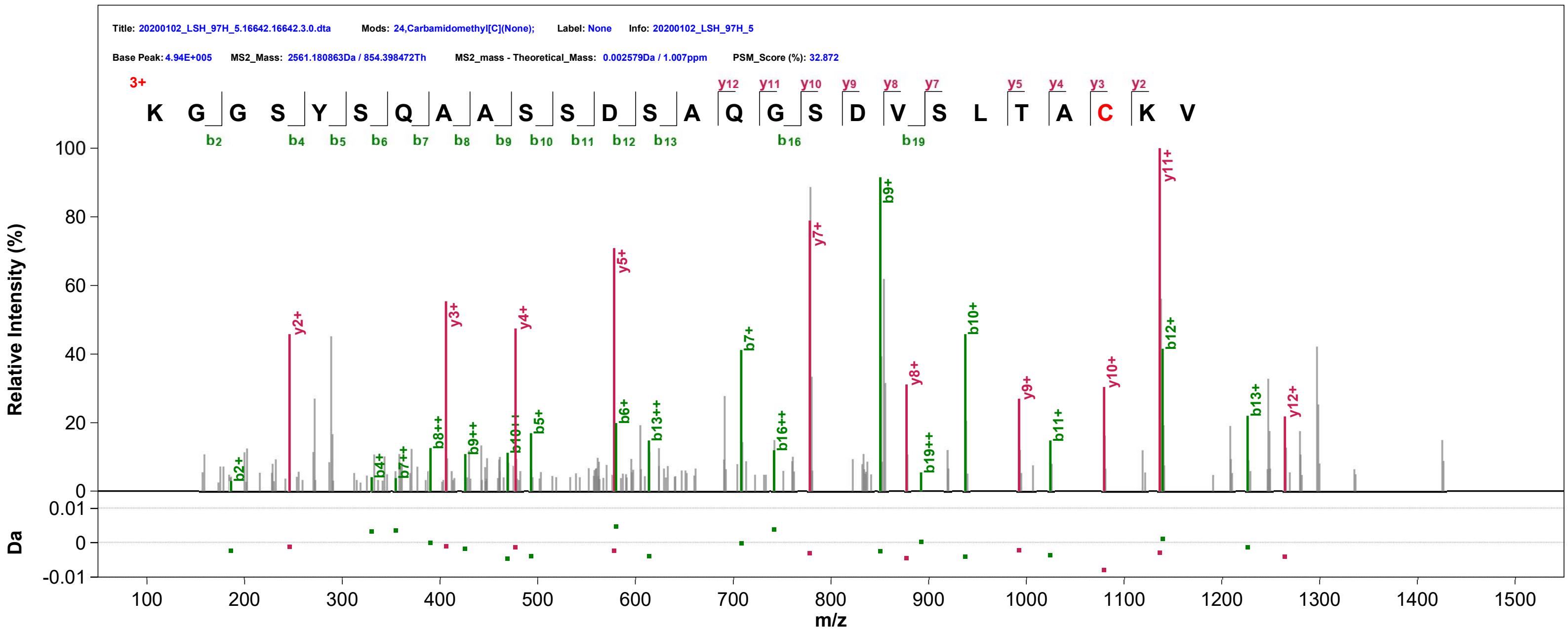

Protein Name: HLA-H_ORF_2

Peptide UUID: 50f5497b-53b0-44a5-96a9-86759cc493dd_HLA-H_ORF_2_3

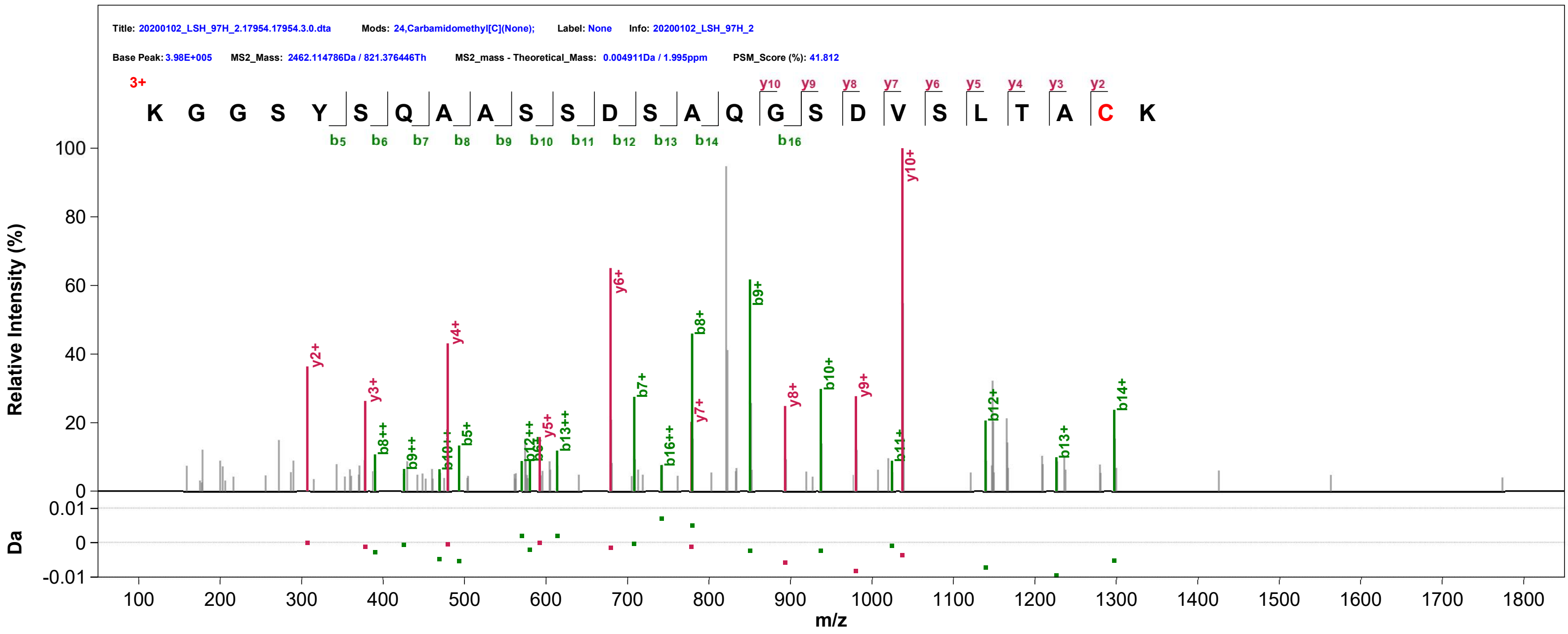

Protein Name: HLA-H_ORF_2
Peptide UUID: 50f5497b-53b0-44a5-96a9-86759cc493dd_HLA-H_ORF_2_4

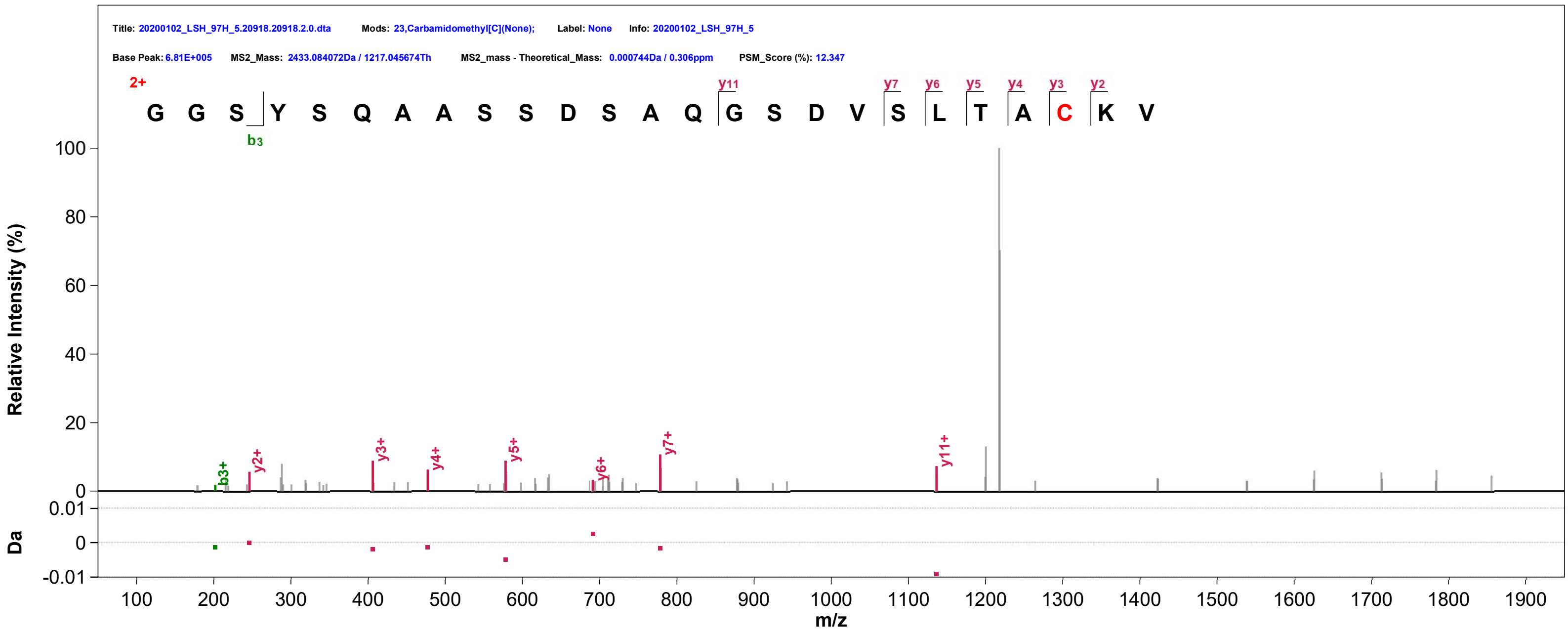

Protein Name: TNK2_ORF_1
Peptide UUID: 53c1baee-50d2-4721-8435-4b1ed626a560_TNK2_ORF_1_2

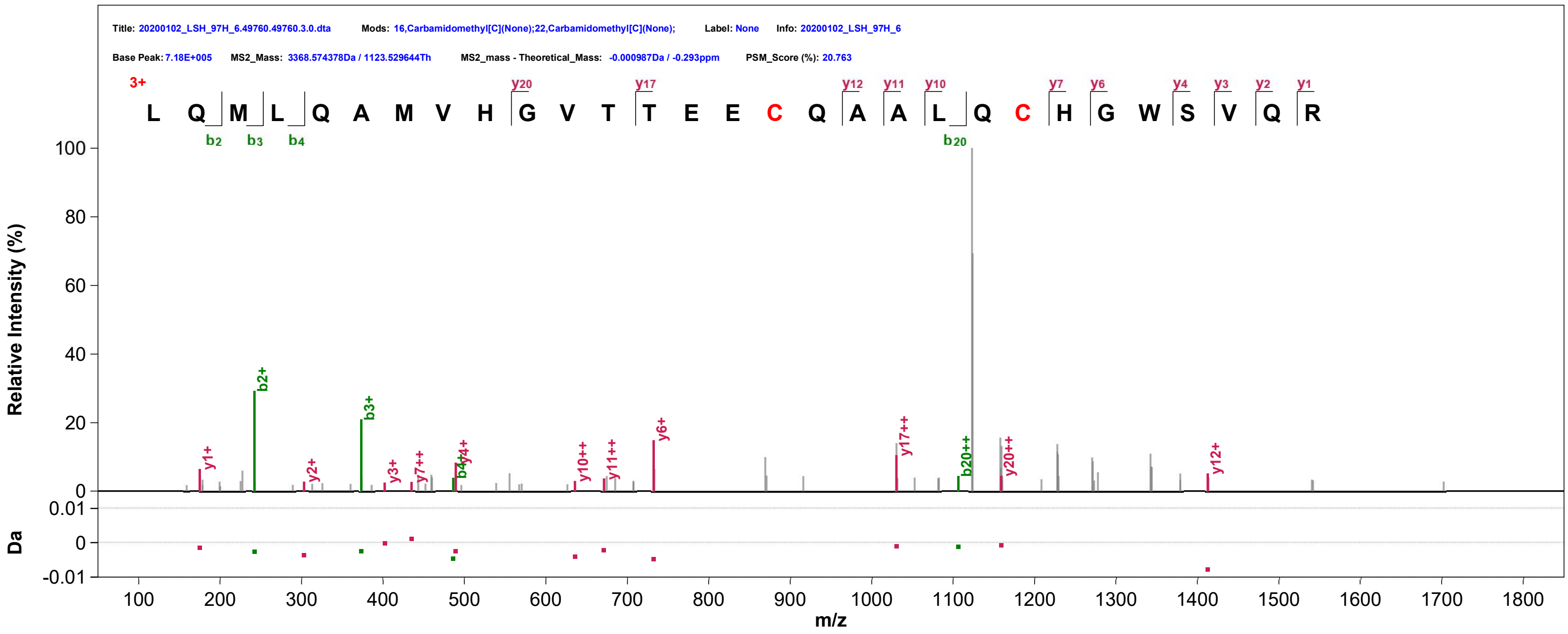

Protein Name: HMGA1_ORF_0
Peptide UUID: 56ceec8d-e3af-49b6-8231-218aa7a1a1c4_HMGA1_ORF_0

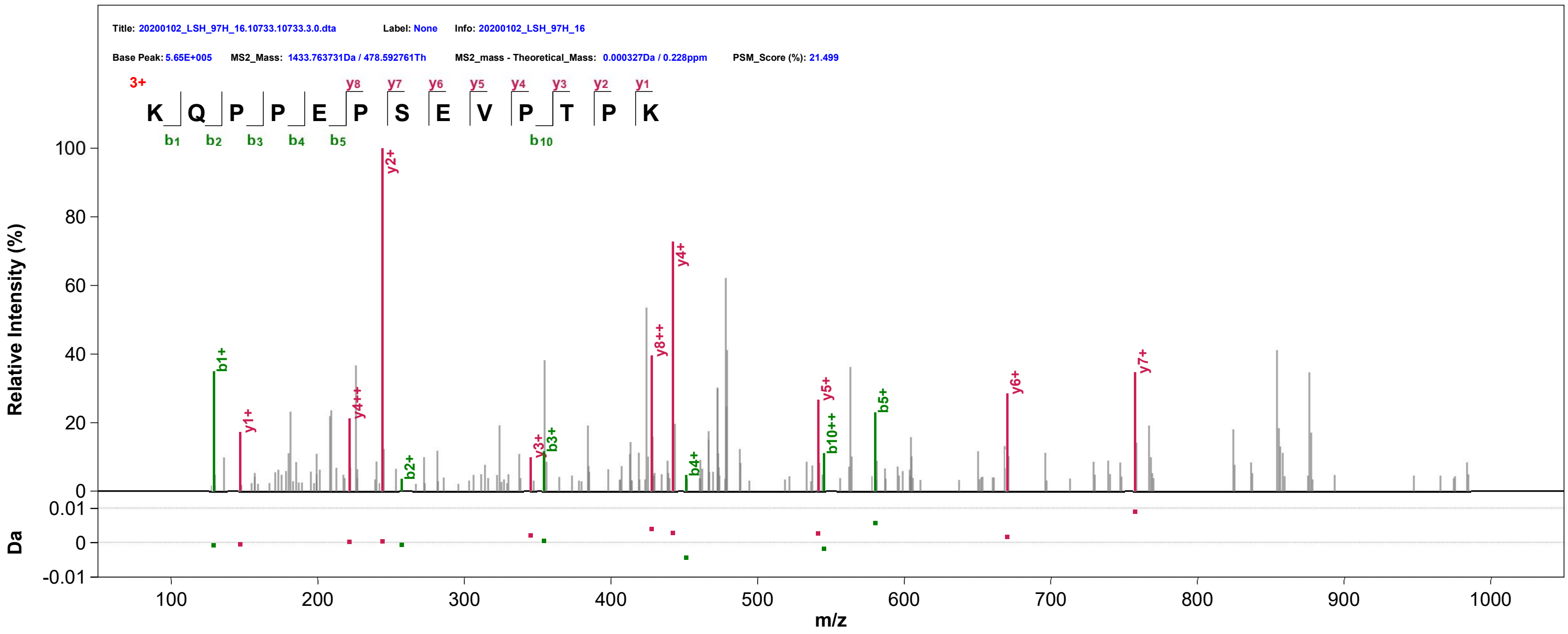

Protein Name: STK38_ORF_0
Peptide UUID: 632c5373-3f04-4494-9e69-c7bcbd5aa06c_STK38_ORF_0

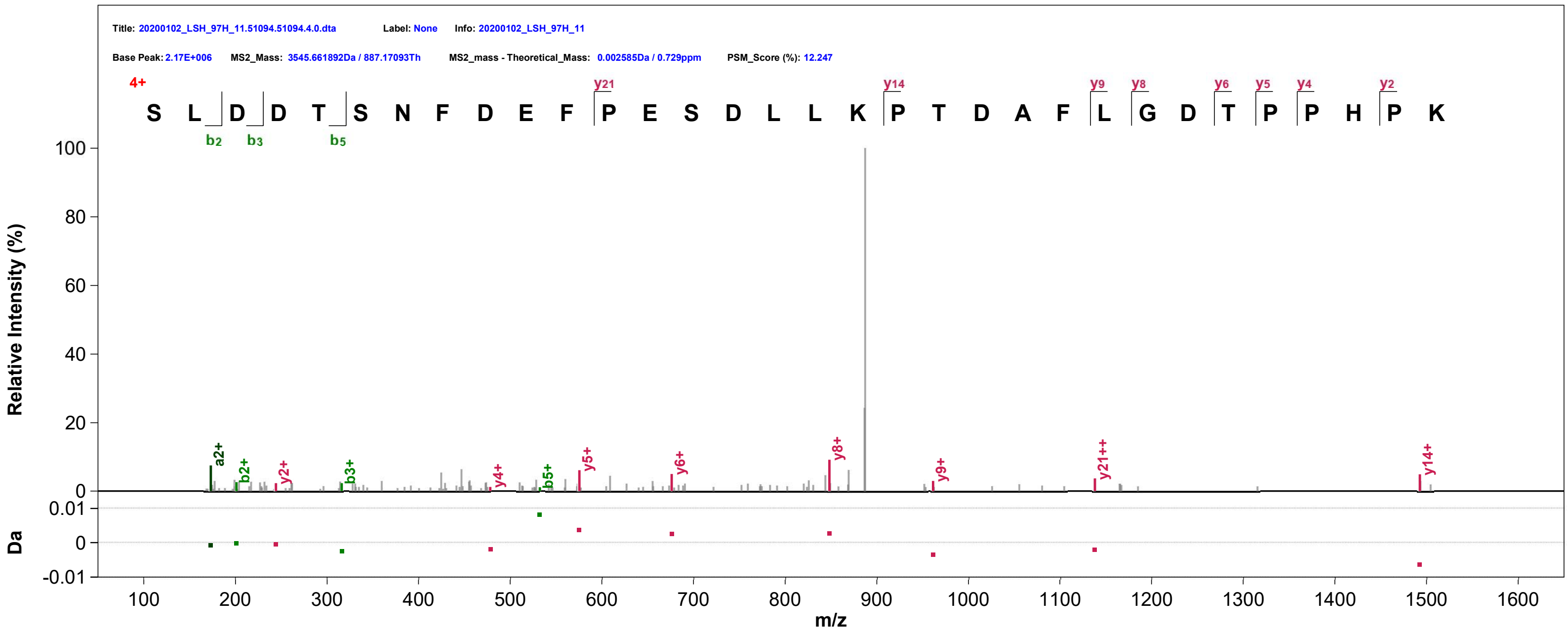

Protein Name: UBL4A_ORF_0
Peptide UUID: 635d7bcc-5546-4446-97e1-dd4806f47a53_UBL4A_ORF_0_1

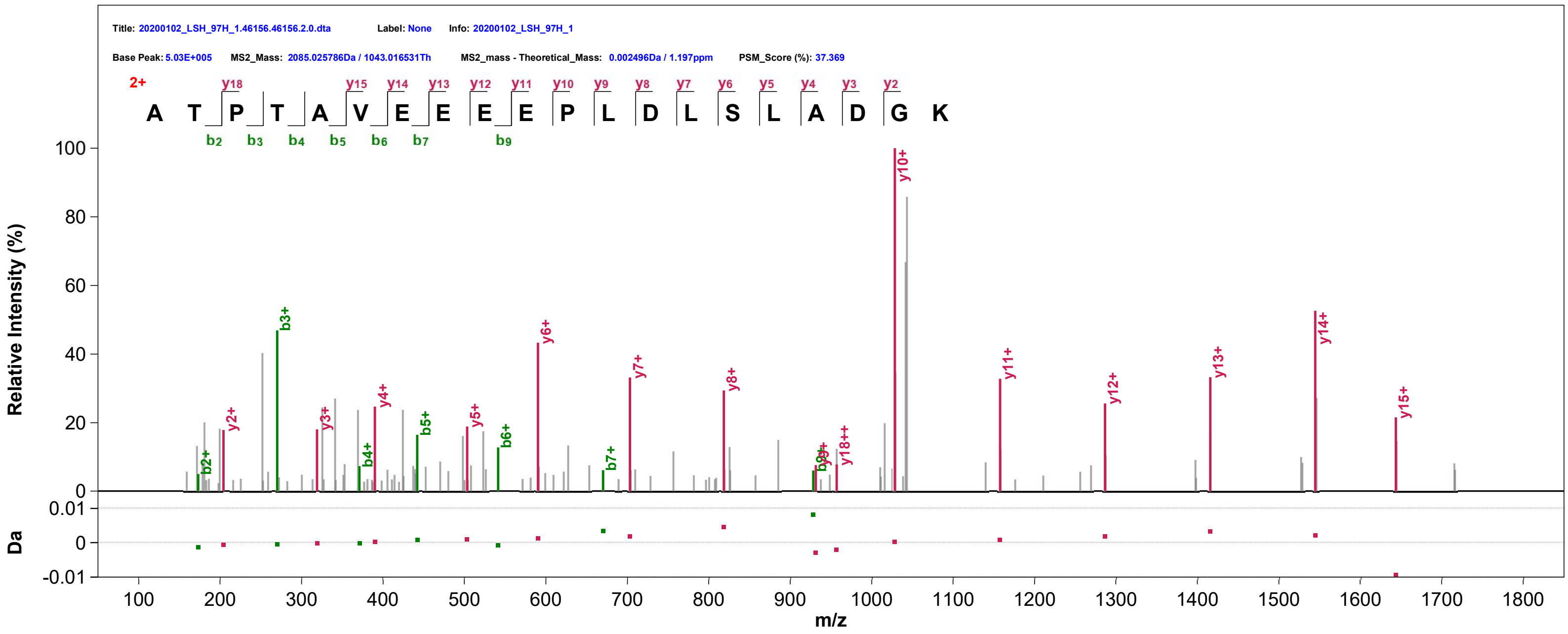

Protein Name: SCAMP3_ORF_0
Peptide UUID: 6425ac44-a04e-4514-871c-c4c3966bf193_SCAMP3_ORF_0

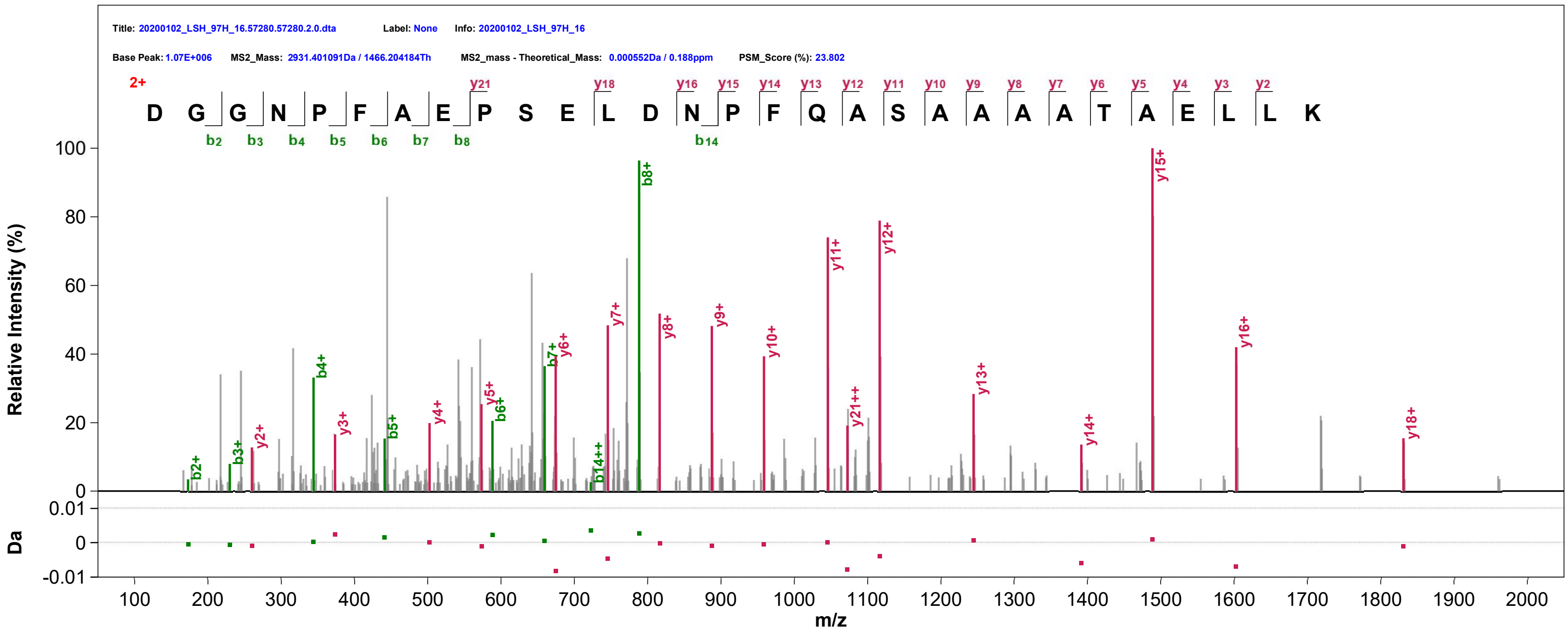

Protein Name: PLEC_ORF_0
Peptide UUID: 68717080-ef6e-40c8-a059-58cc32ad718f_PLEC_ORF_0_3

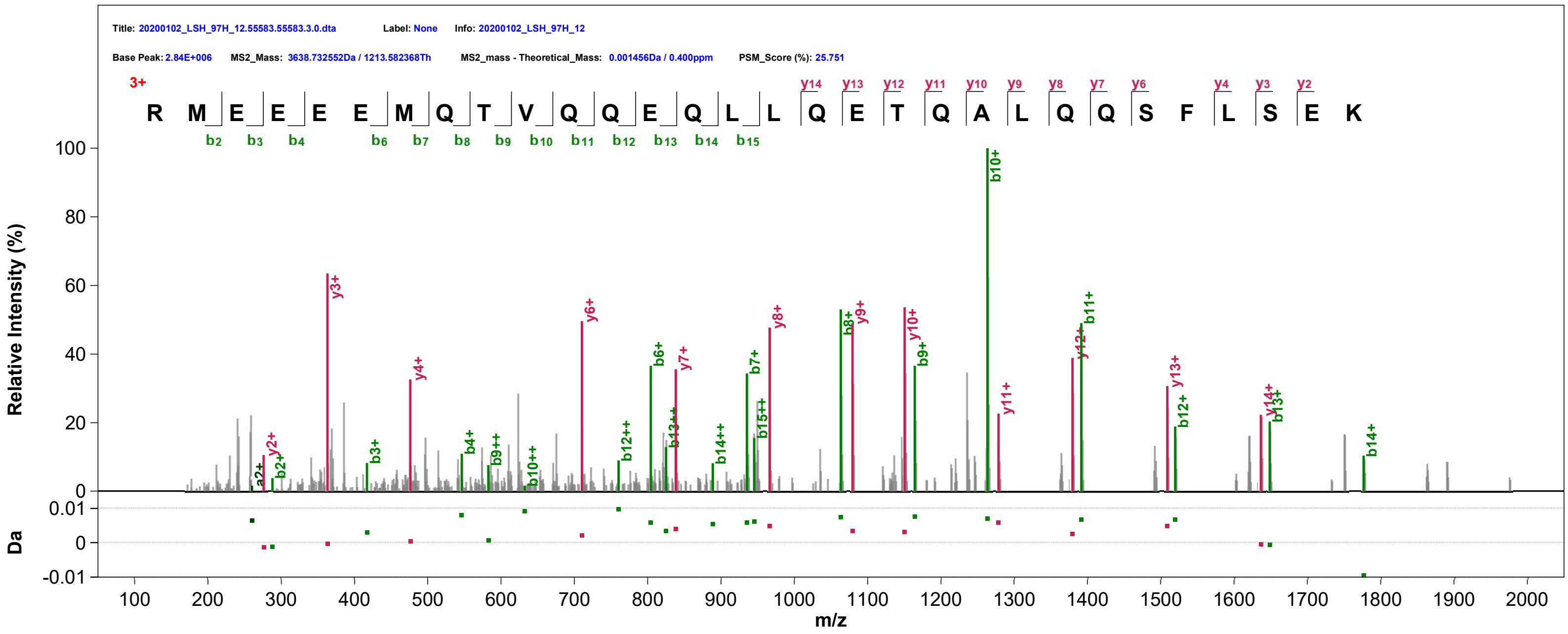

Protein Name: PLEC_ORF_0
Peptide UUID: 68717080-ef6e-40c8-a059-58cc32ad718f_PLEC_ORF_0_12

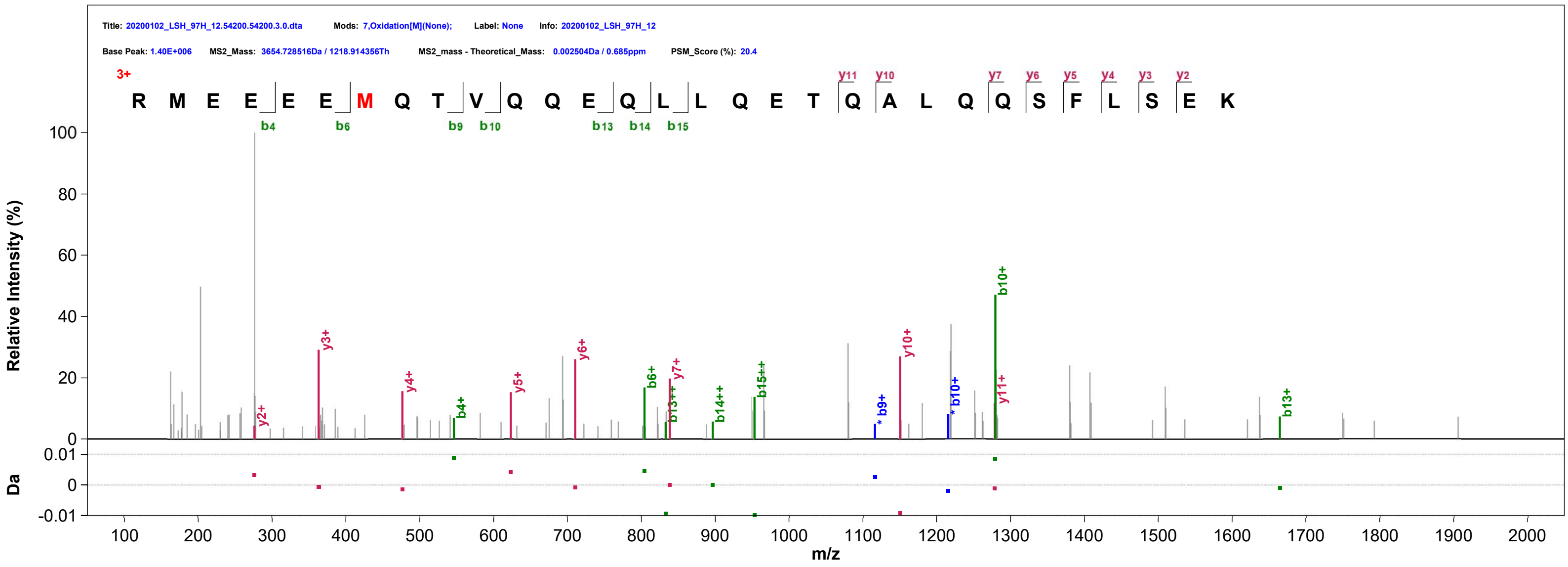

Protein Name: PLEC_ORF_0
Peptide UUID: 68717080-ef6e-40c8-a059-58cc32ad718f_PLEC_ORF_0_4

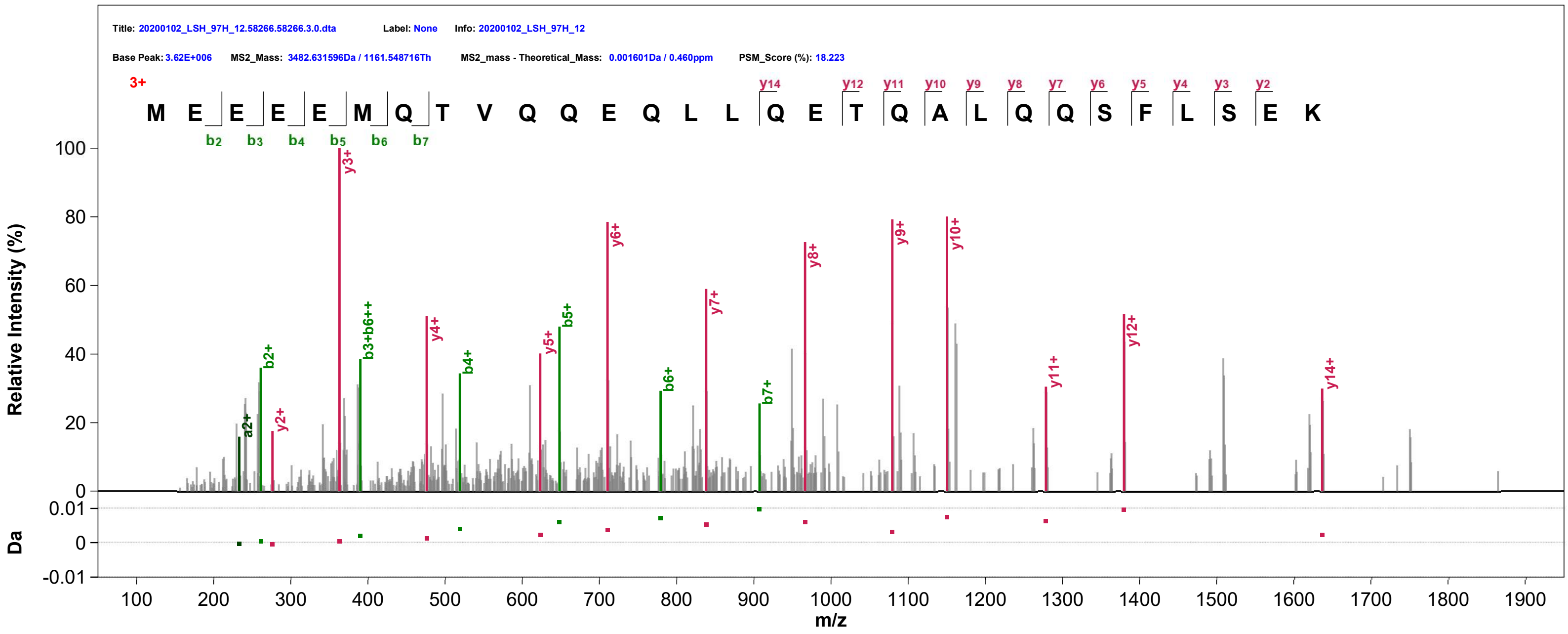

Protein Name: PLEC_ORF_0
Peptide UUID: 68717080-ef6e-40c8-a059-58cc32ad718f_PLEC_ORF_0_8

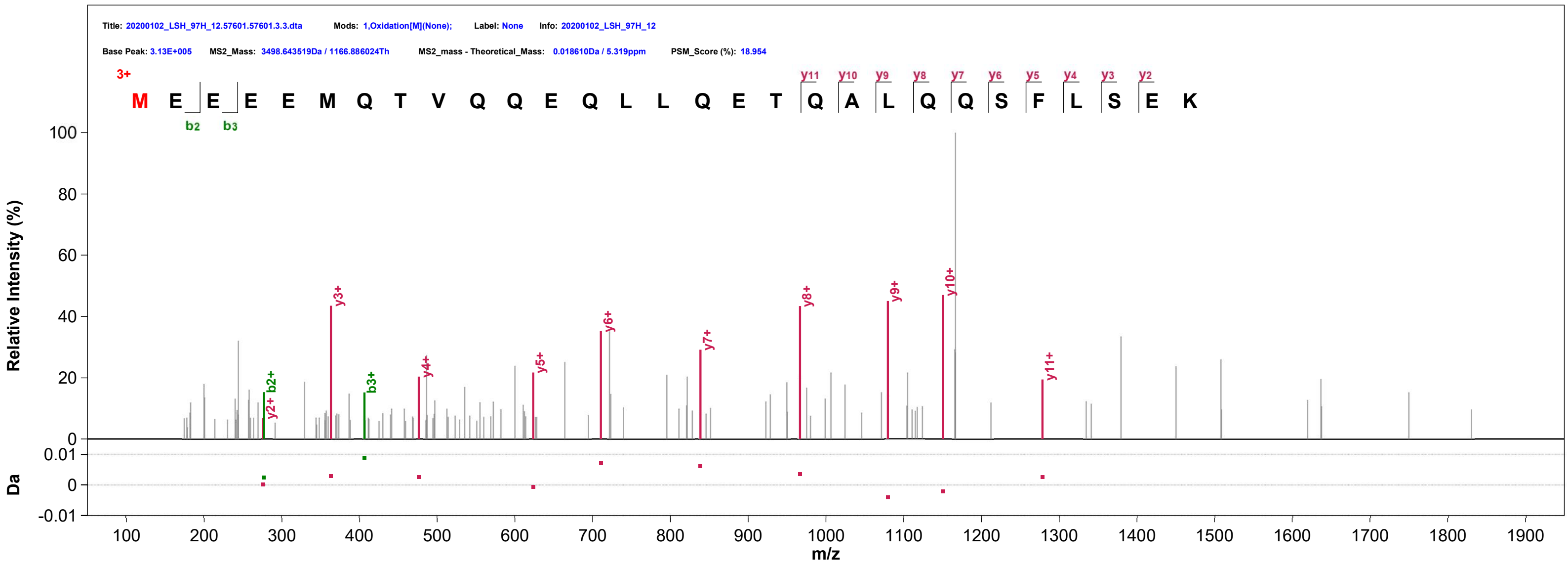

Protein Name: CLASRP_ORF_0
Peptide UUID: 7430a9a0-4994-4778-8f0d-82c007641f34_CLASRP_ORF_0_4

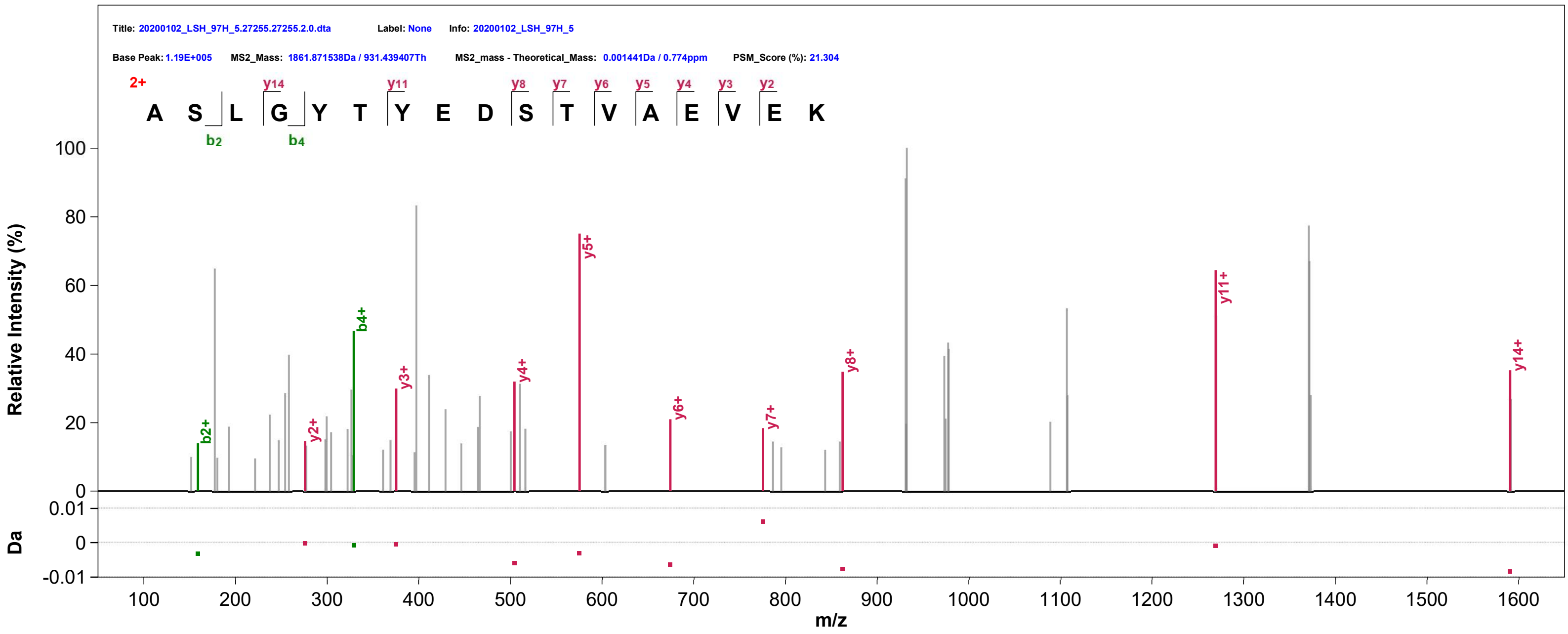

Protein Name: SCLY_ORF_2
Peptide UUID: 78feb568-08da-44c3-a909-f44fa185f148_SCLY_ORF_2

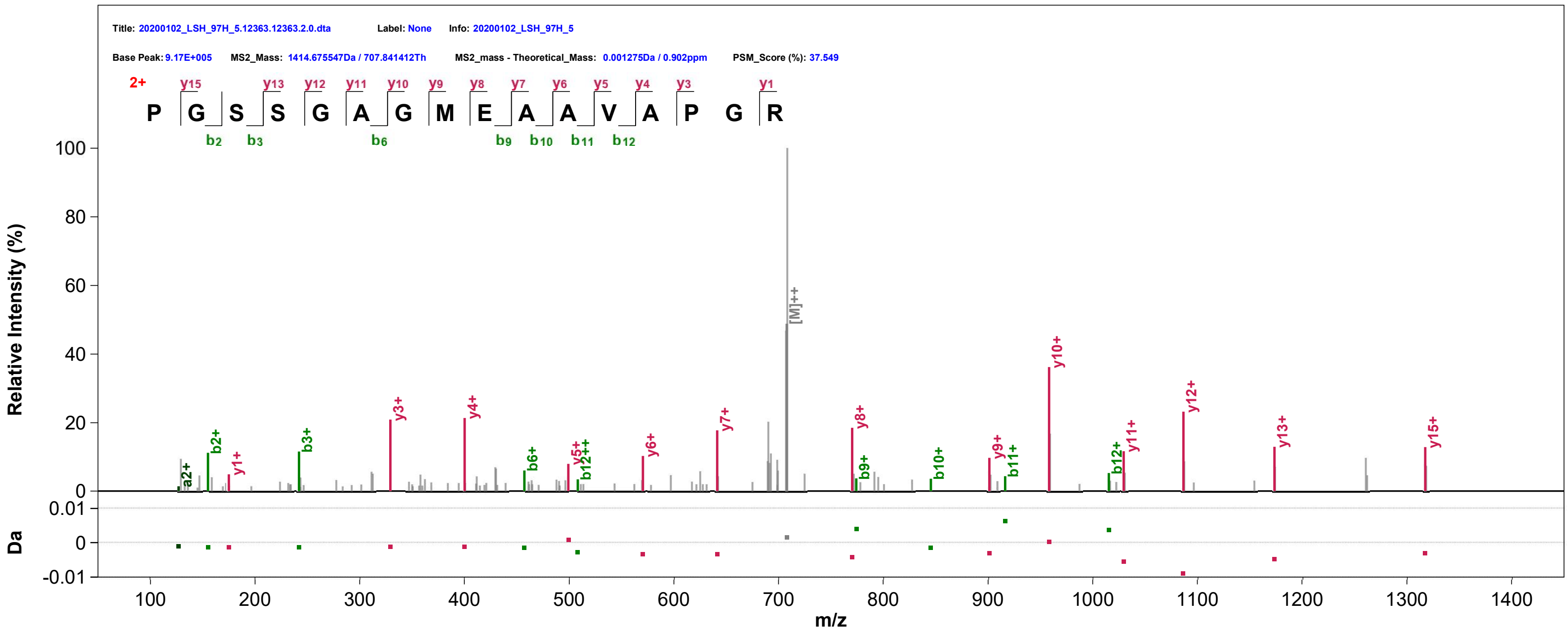

Protein Name: HNRNPL_ORF_0
Peptide UUID: 7c2d8540-40af-4645-8aab-69160c336310_HNRNPL_ORF_0_1

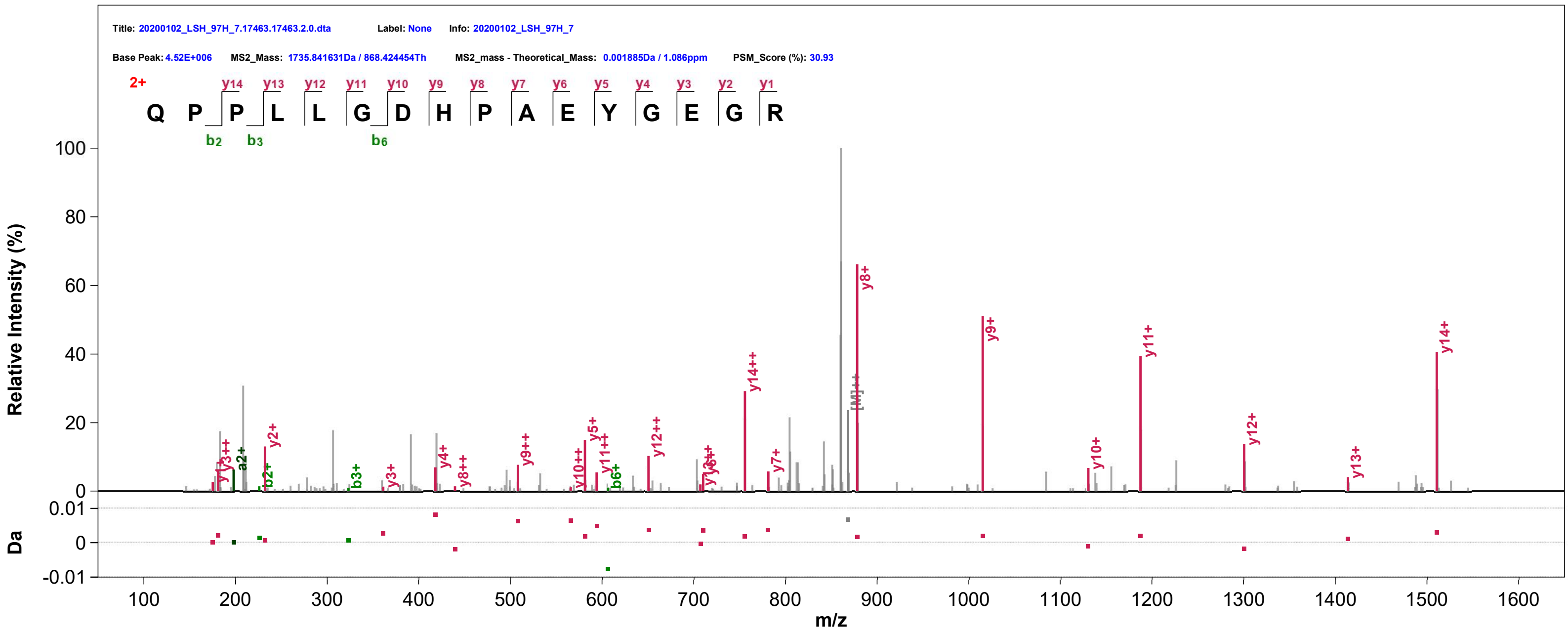

Protein Name: TAF8_ORF_0
Peptide UUID: 7f5ba327-41b3-439b-9138-c3d3505a04ee_TAF8_ORF_0_2

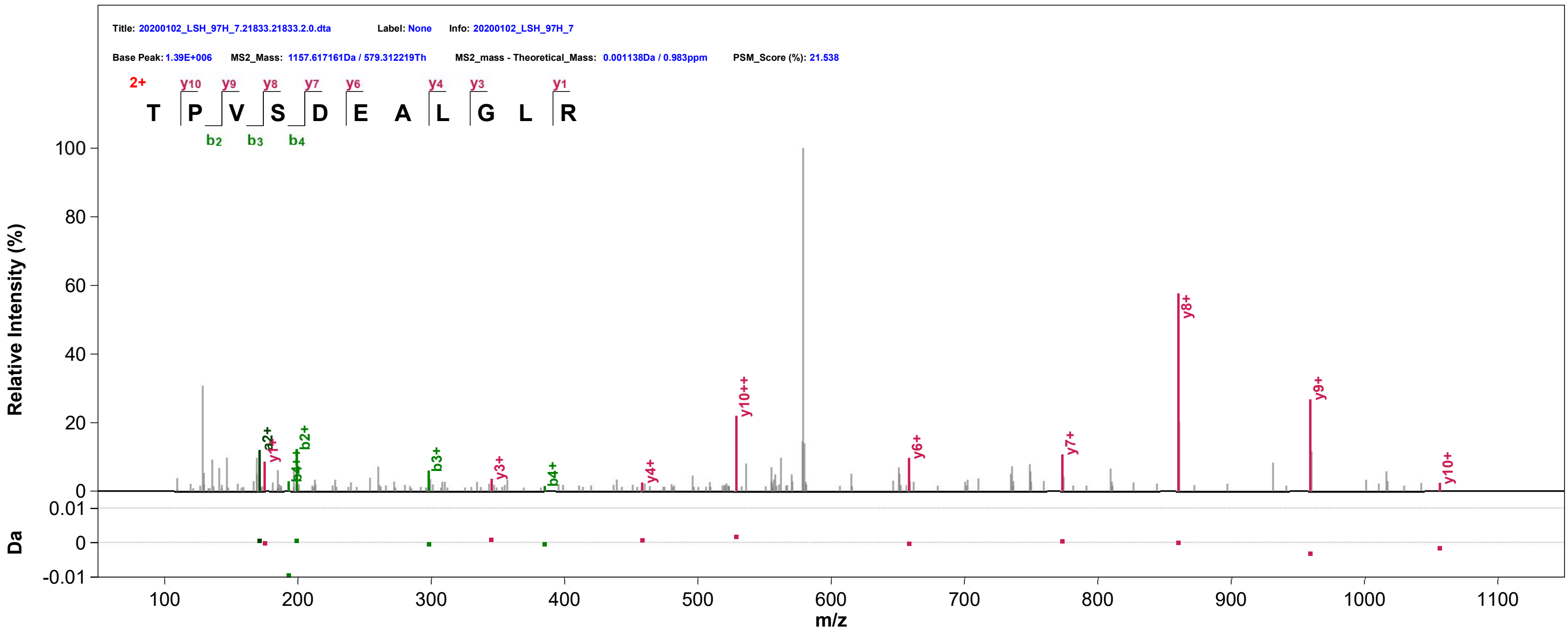

Protein Name: KIF16B_ORF_0
Peptide UUID: 833a01f2-0478-4011-9370-f75003b78c16_KIF16B_ORF_0_1

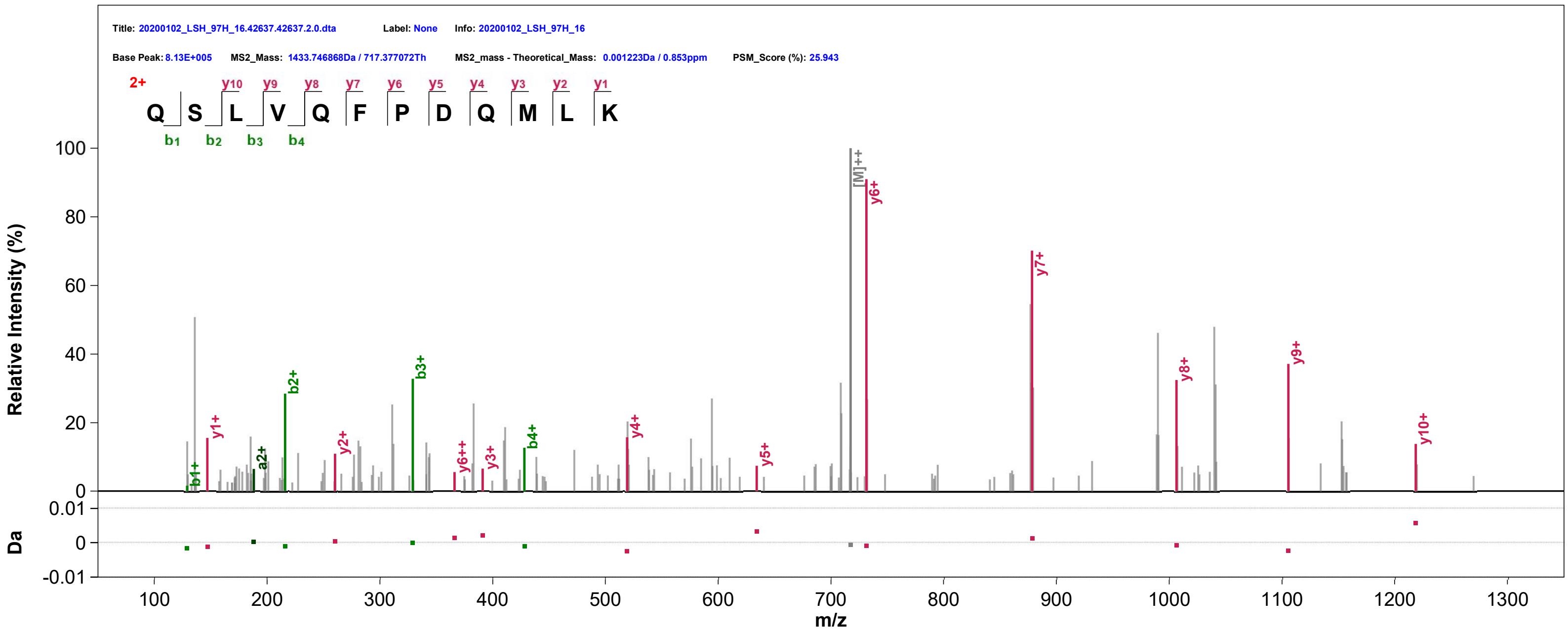

Protein Name: KIF16B_ORF_0
Peptide UUID: 833a01f2-0478-4011-9370-f75003b78c16_KIF16B_ORF_0_2

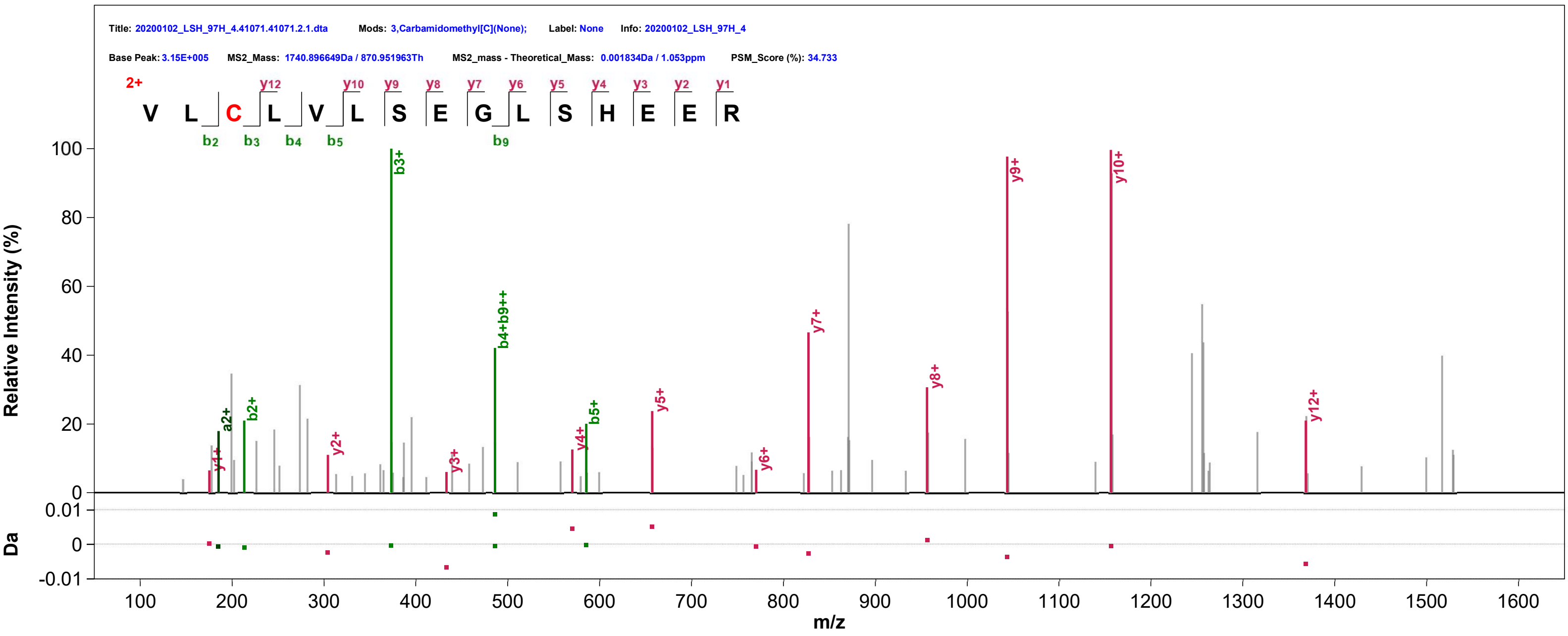

Protein Name: KIF16B_ORF_0
Peptide UUID: 833a01f2-0478-4011-9370-f75003b78c16_KIF16B_ORF_0_4

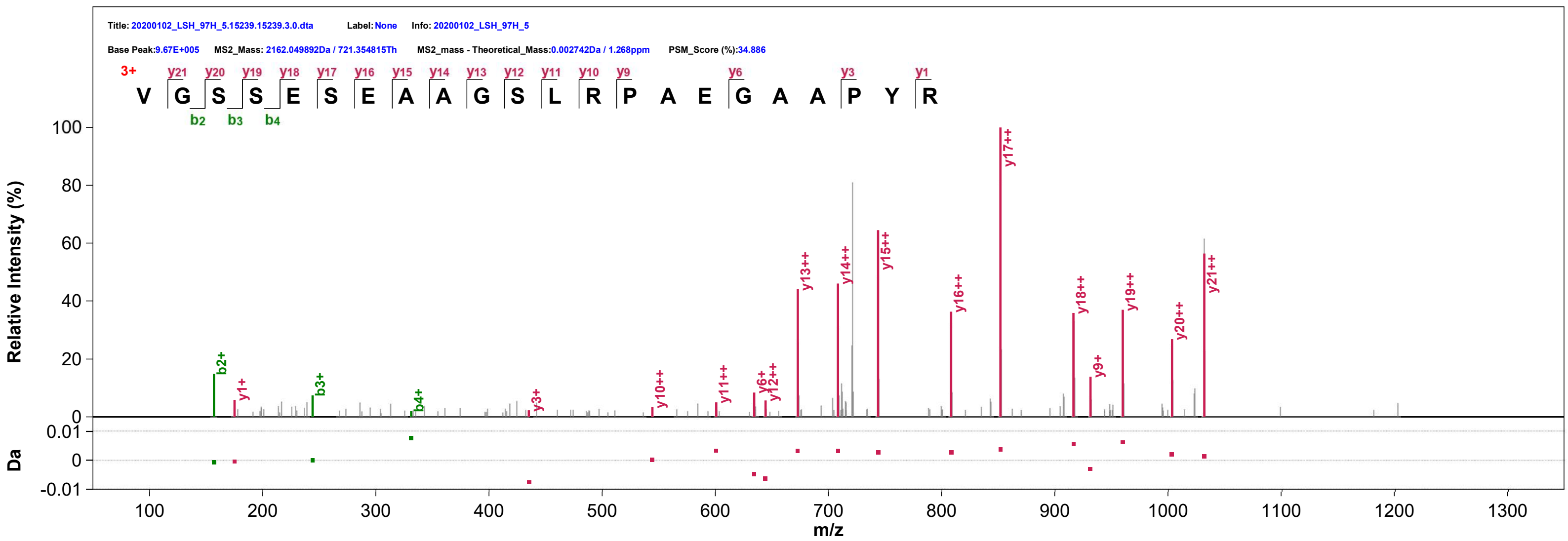

Protein Name: KIF16B_ORF_0
Peptide UUID: 833a01f2-0478-4011-9370-f75003b78c16_KIF16B_ORF_0_5

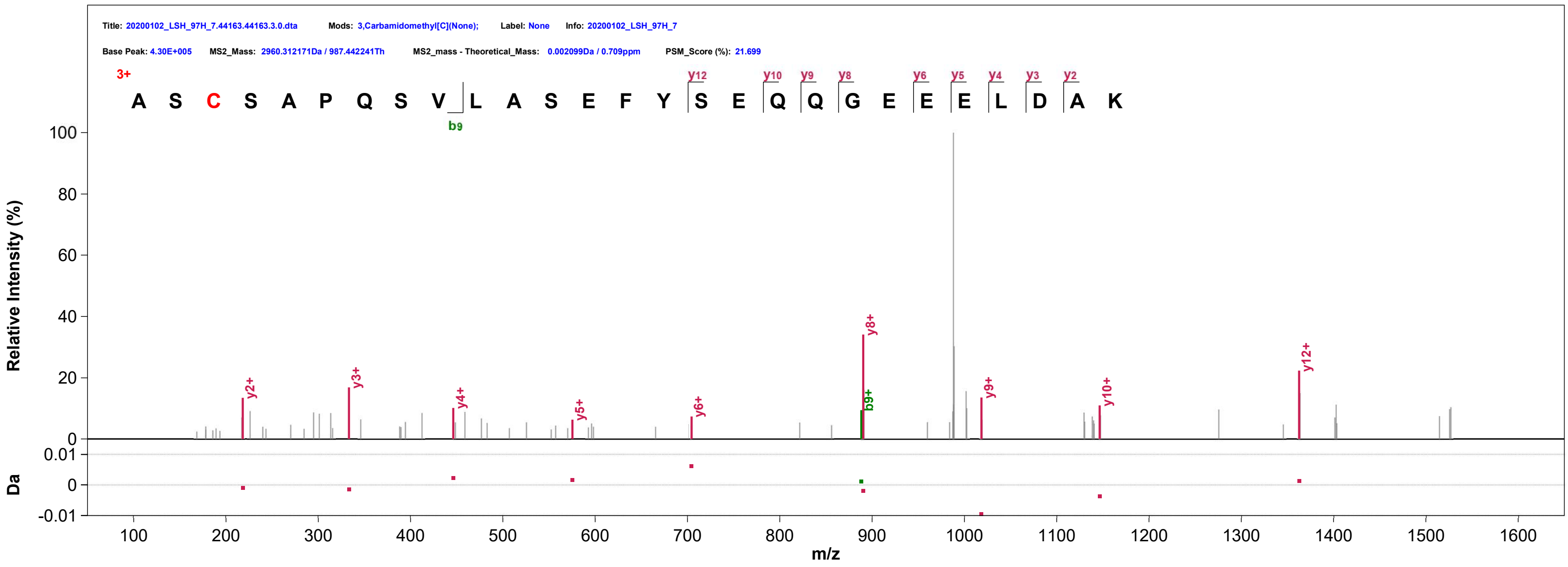

Protein Name: KIF16B_ORF_0
Peptide UUID: 833a01f2-0478-4011-9370-f75003b78c16_KIF16B_ORF_0_7

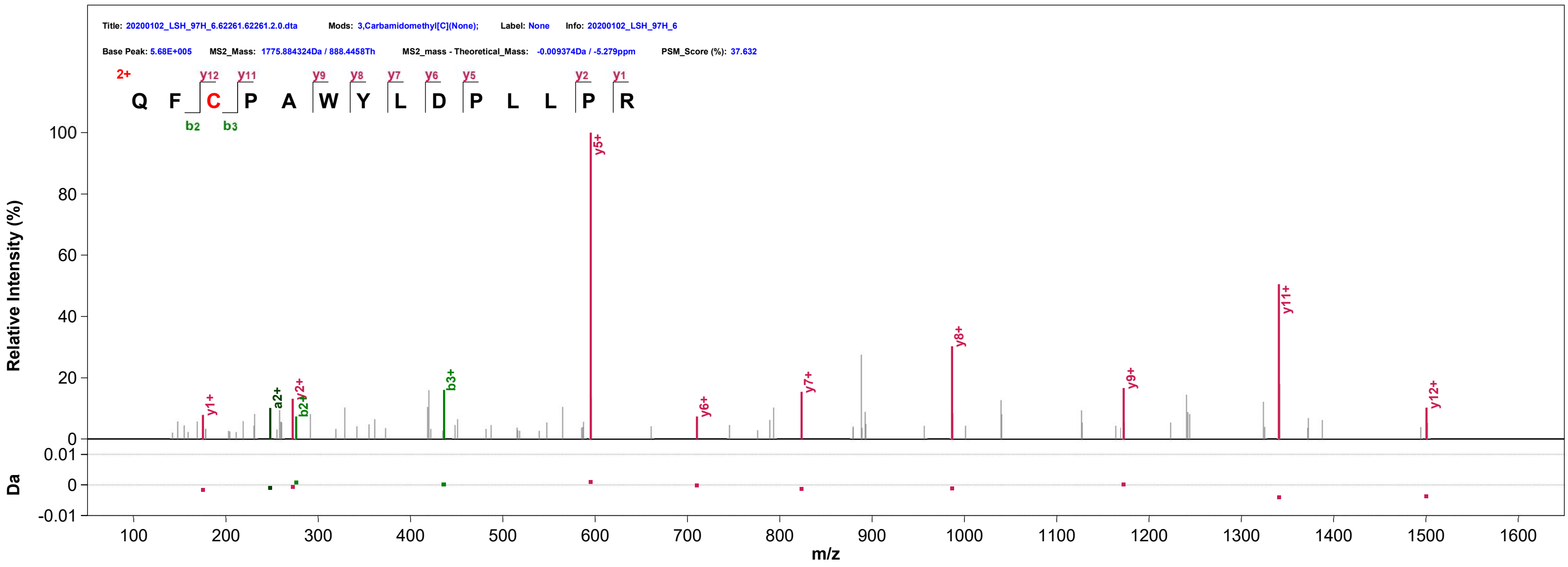

Protein Name: KIF16B_ORF_0
Peptide UUID: 833a01f2-0478-4011-9370-f75003b78c16_KIF16B_ORF_0_8

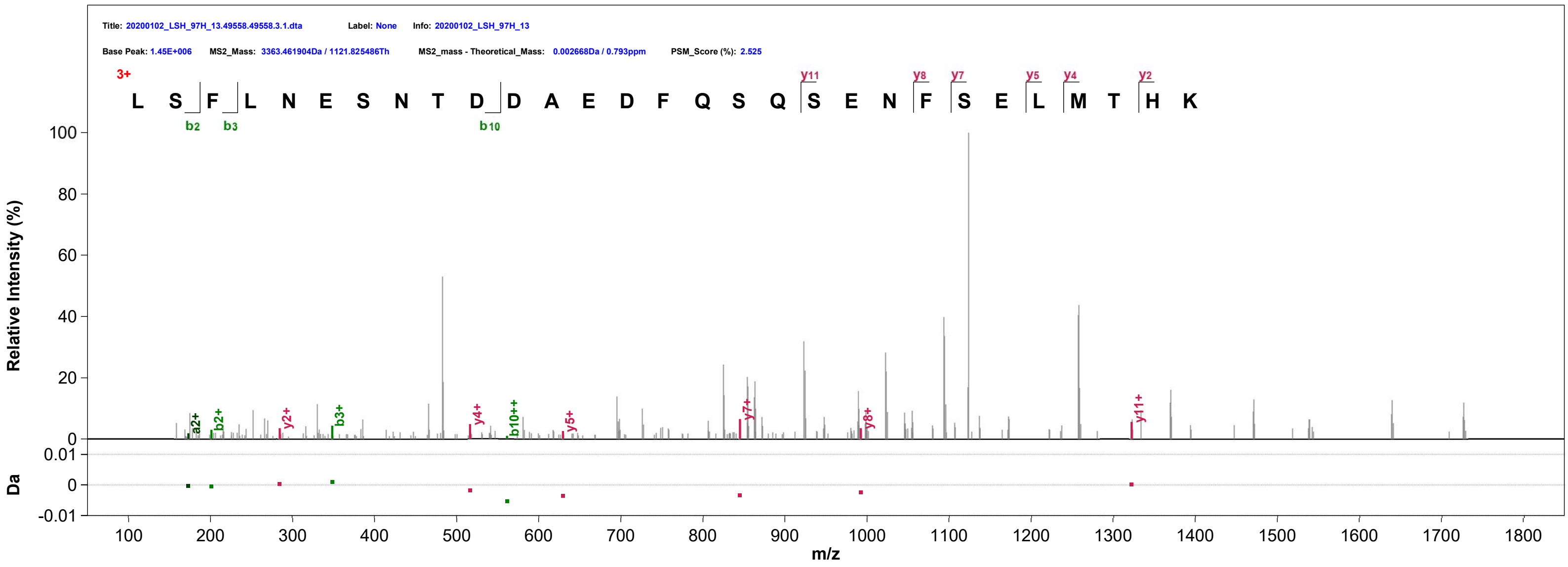

Protein Name: KIF16B_ORF_0
Peptide UUID: 833a01f2-0478-4011-9370-f75003b78c16_KIF16B_ORF_0_9

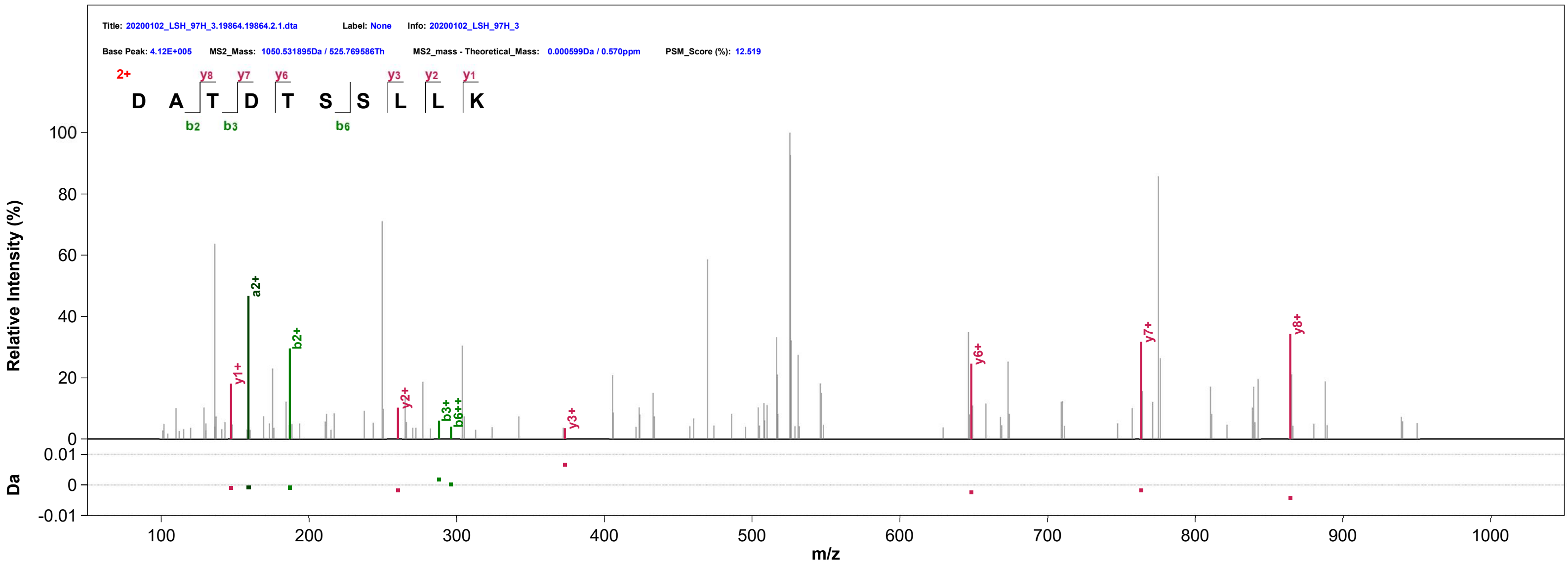

Protein Name: TXNRD2_ORF_0
Peptide UUID: 842dc850-db23-48cc-84b5-c80a255c50e9_TXNRD2_ORF_0_1

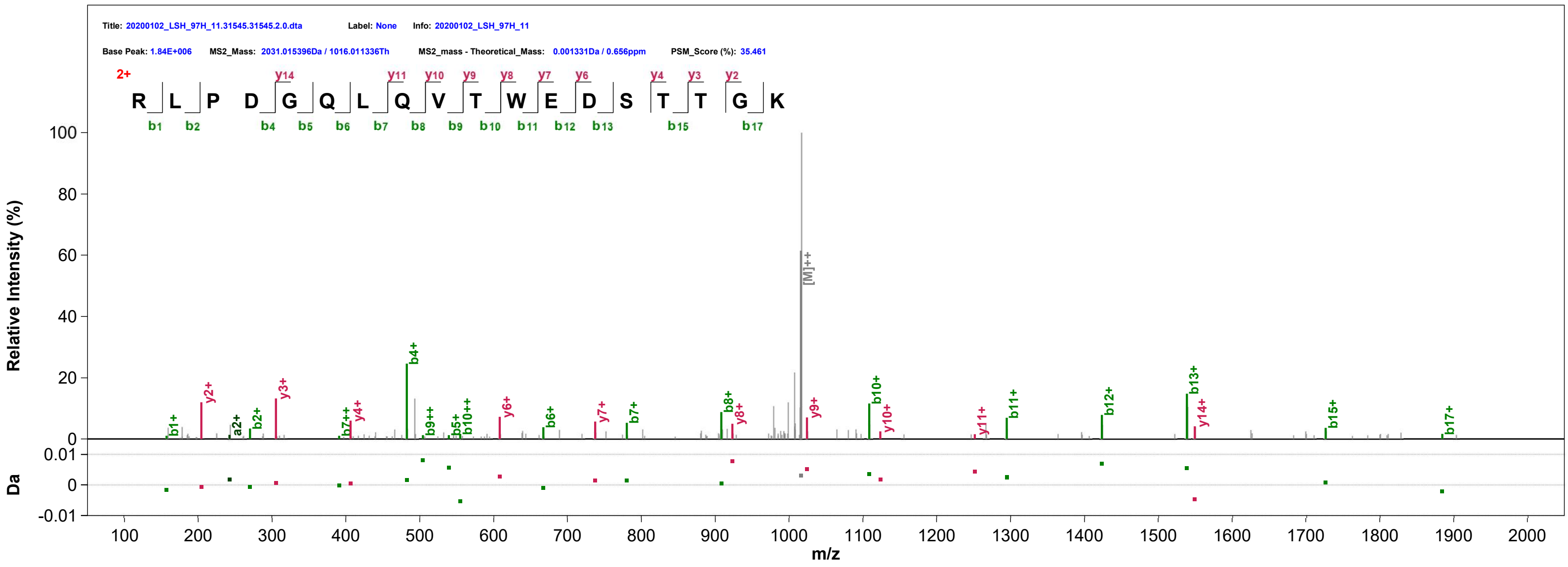

Protein Name: TXNRD2_ORF_0
Peptide UUID: 842dc850-db23-48cc-84b5-c80a255c50e9_TXNRD2_ORF_0_2

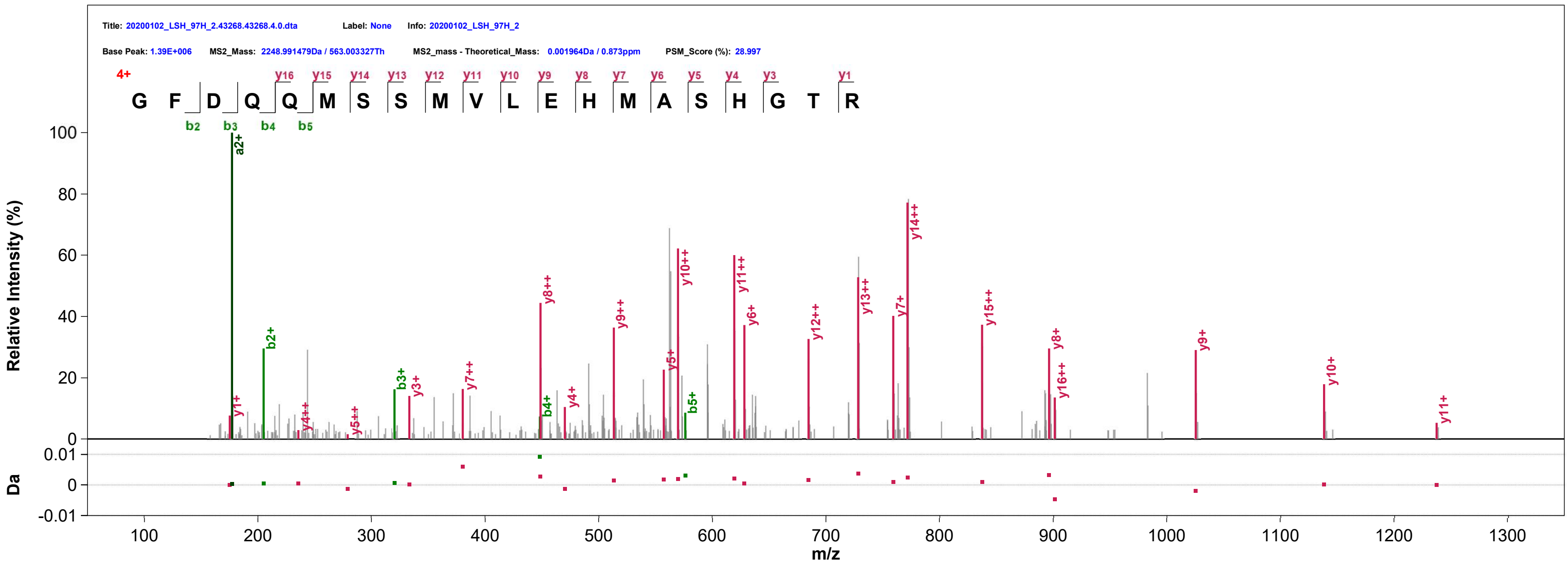

Protein Name: TXNRD2_ORF_0
Peptide UUID: 842dc850-db23-48cc-84b5-c80a255c50e9_TXNRD2_ORF_0_3

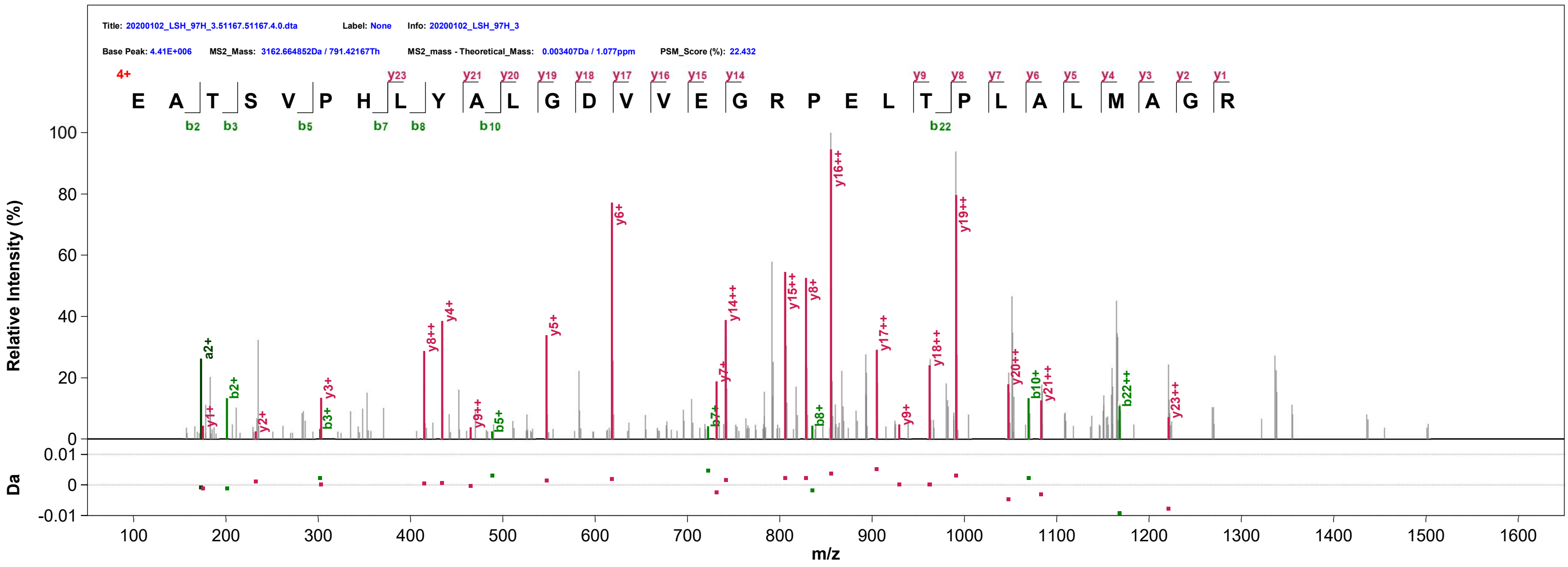

Protein Name: TXNRD2_ORF_0
Peptide UUID: 842dc850-db23-48cc-84b5-c80a255c50e9_TXNRD2_ORF_0_4

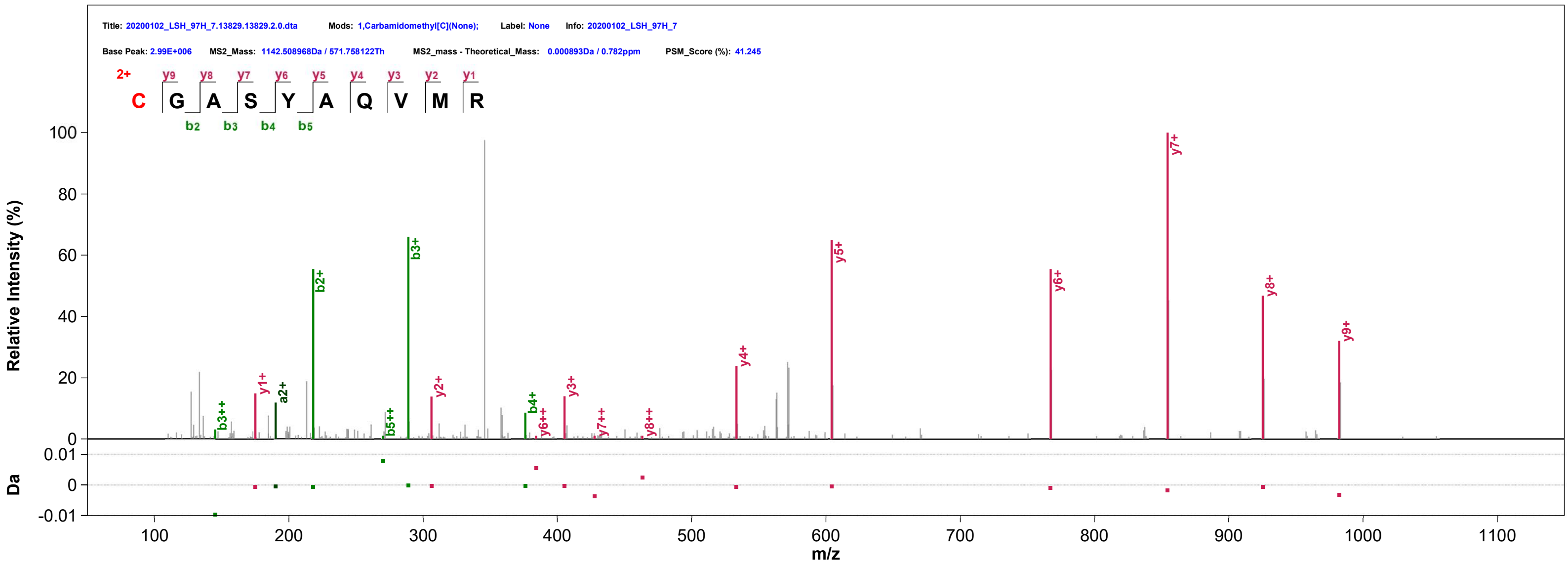

Protein Name: TXNRD2_ORF_0
Peptide UUID: 842dc850-db23-48cc-84b5-c80a255c50e9_TXNRD2_ORF_0_5

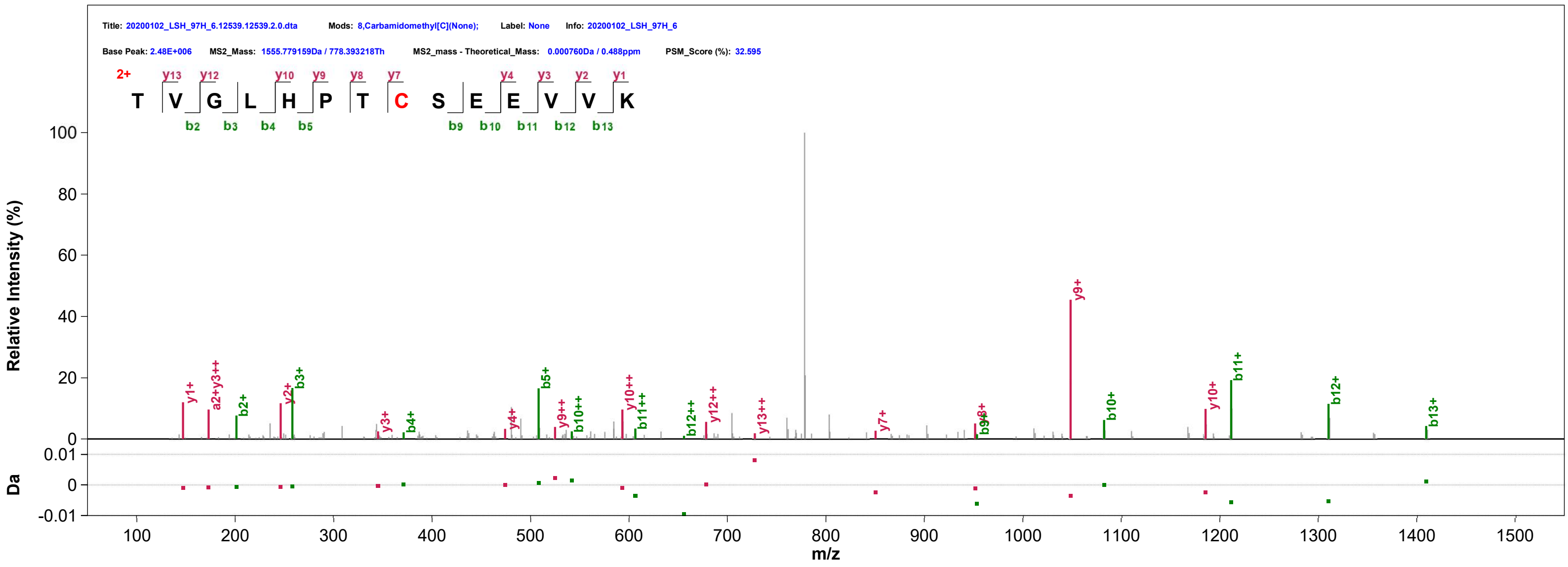

Protein Name: TXNRD2_ORF_0
Peptide UUID: 842dc850-db23-48cc-84b5-c80a255c50e9_TXNRD2_ORF_0_6

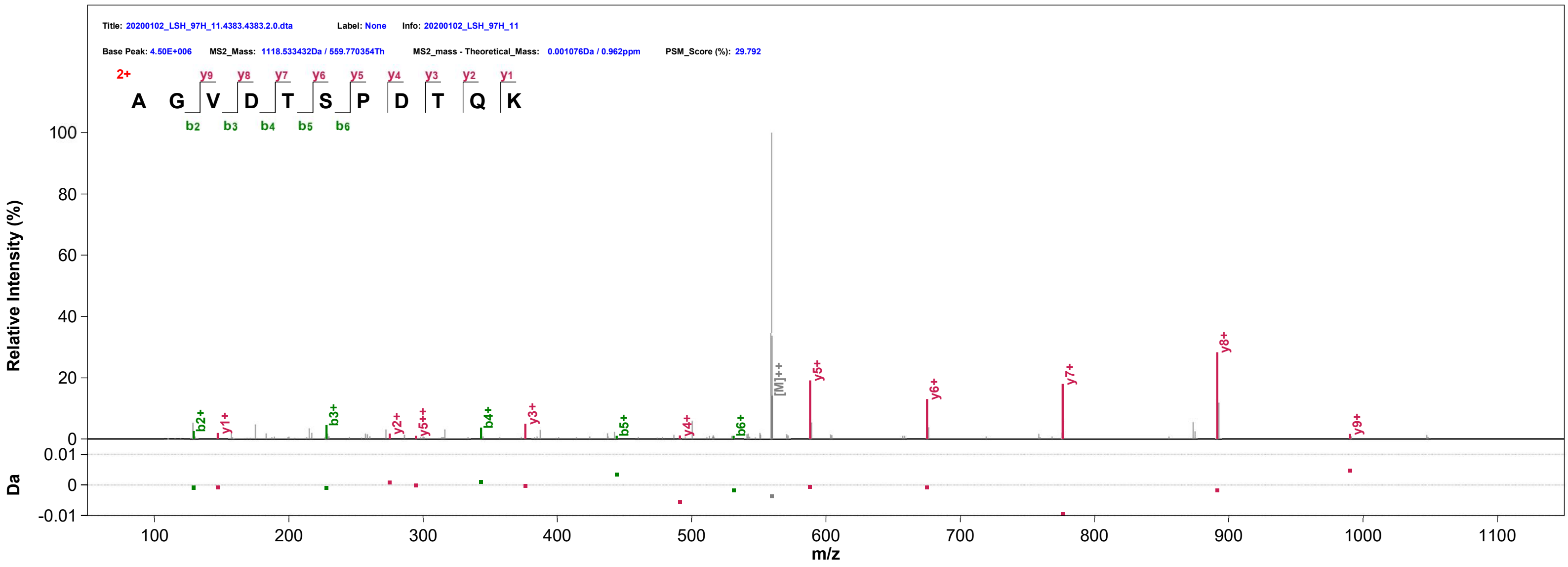

Protein Name: TXNRD2_ORF_2
Peptide UUID: 842dc850-db23-48cc-84b5-c80a255c50e9_TXNRD2_ORF_2_1

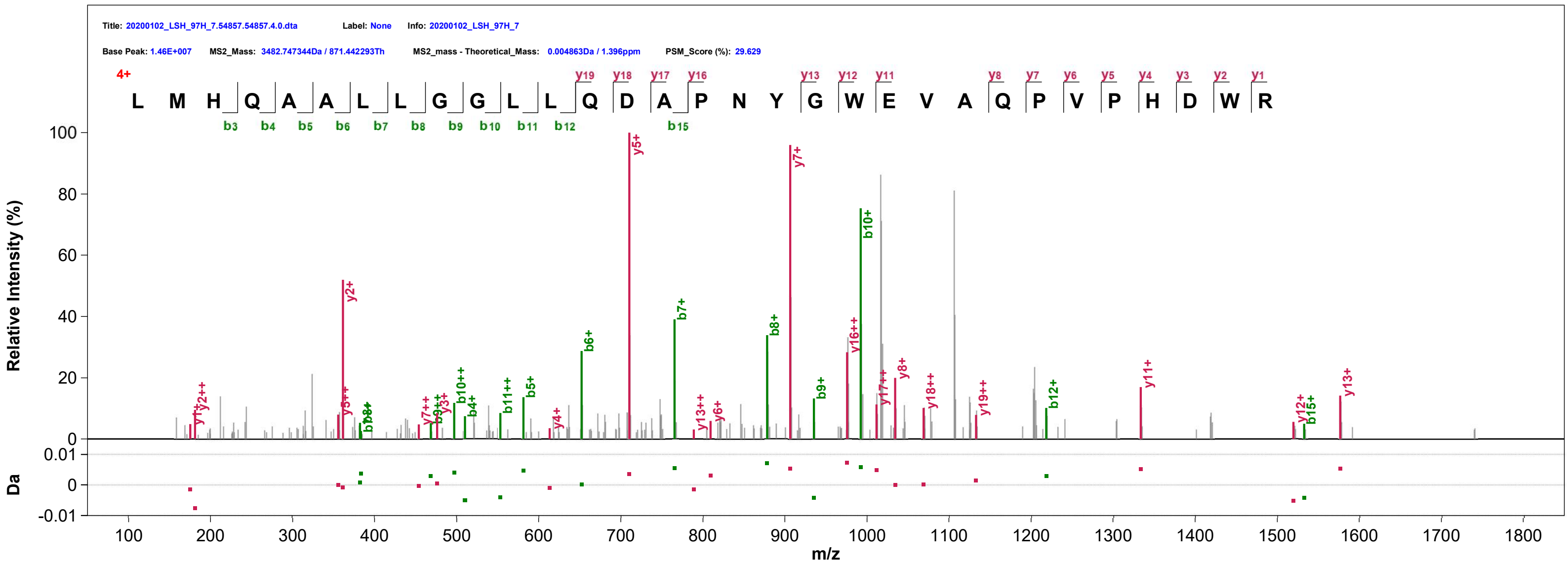

Protein Name: TXNRD2_ORF_2
Peptide UUID: 842dc850-db23-48cc-84b5-c80a255c50e9_TXNRD2_ORF_2_2

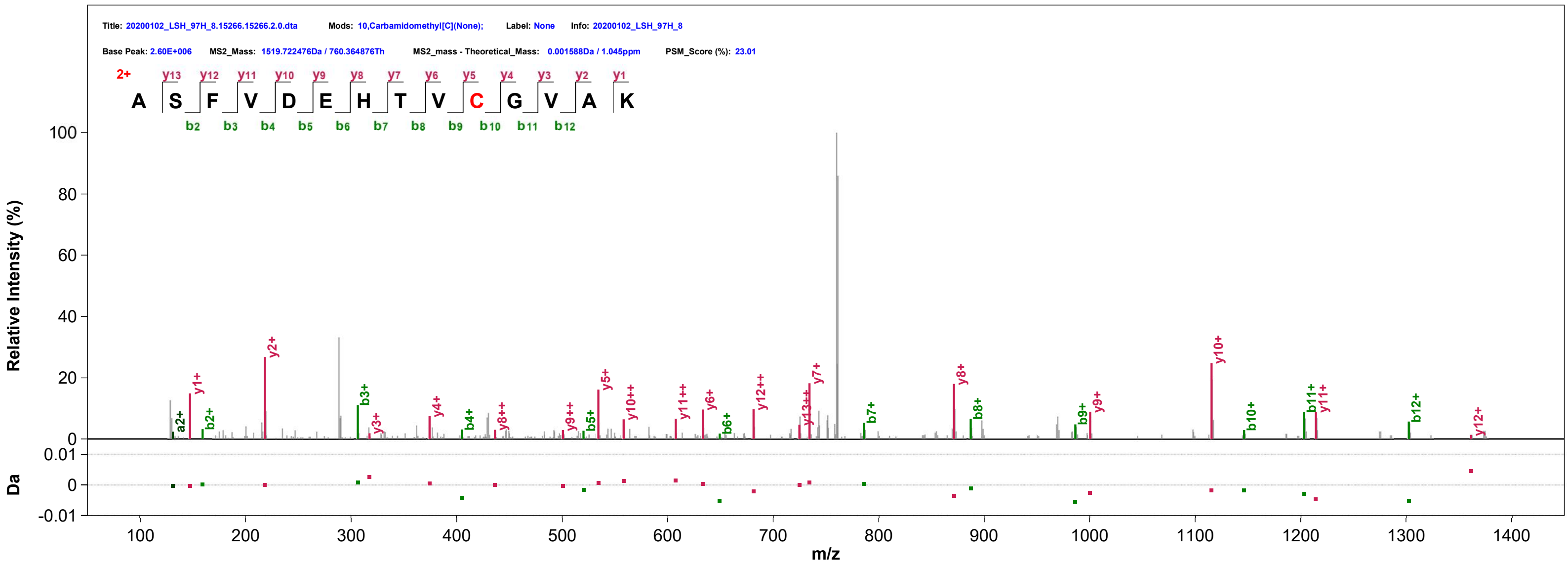

Protein Name: TXNRD2_ORF_2
Peptide UUID: 842dc850-db23-48cc-84b5-c80a255c50e9_TXNRD2_ORF_2_3

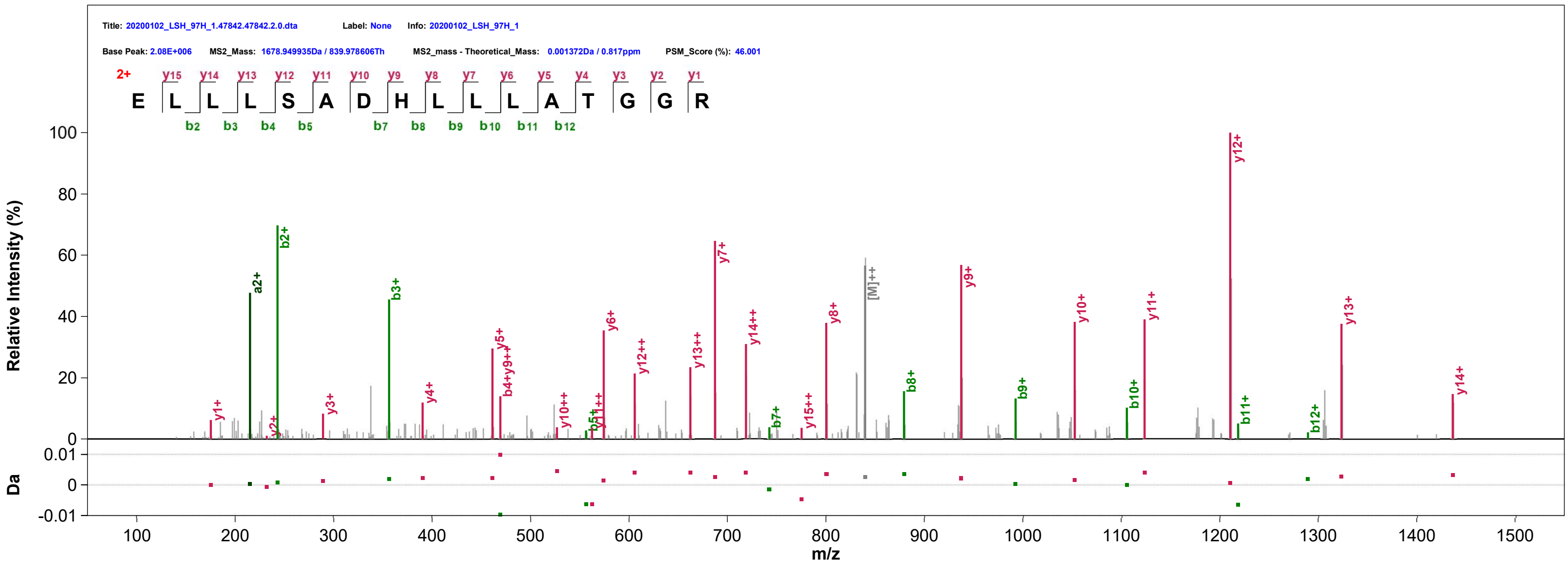

Protein Name: TXNRD2_ORF_2
Peptide UUID: 842dc850-db23-48cc-84b5-c80a255c50e9_TXNRD2_ORF_2_4

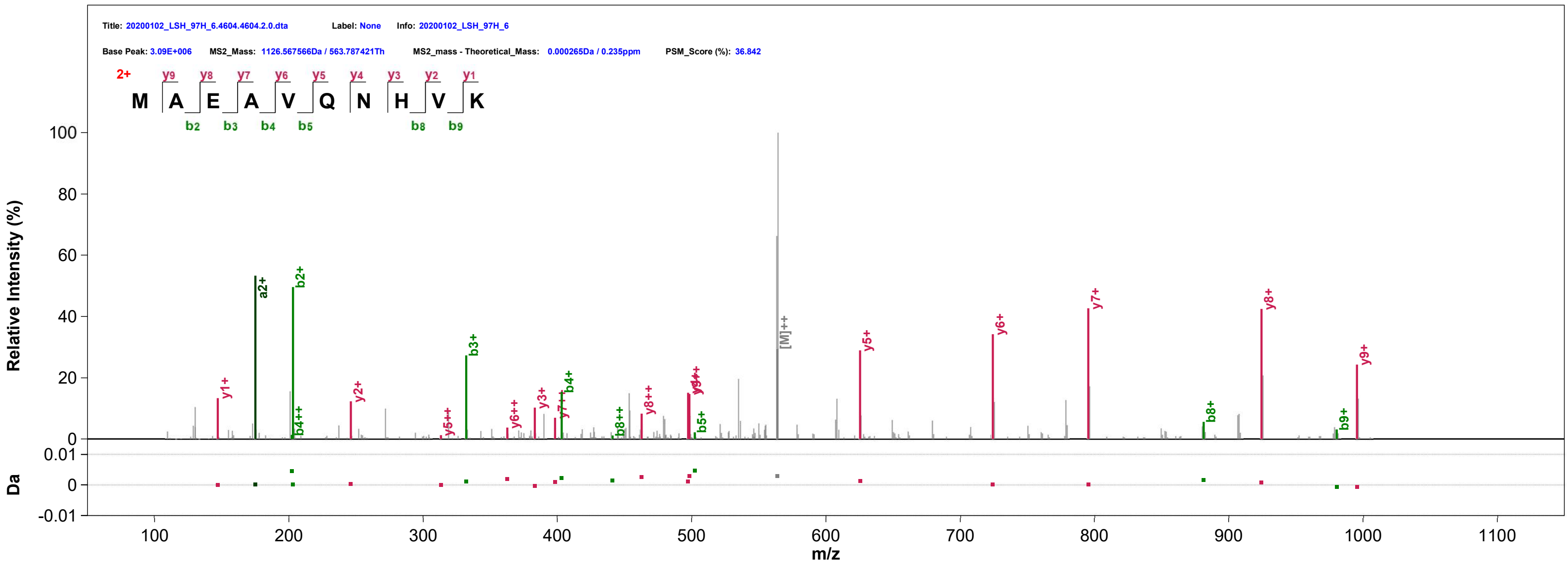

Protein Name: TXNRD2_ORF_2
Peptide UUID: 842dc850-db23-48cc-84b5-c80a255c50e9_TXNRD2_ORF_2_7

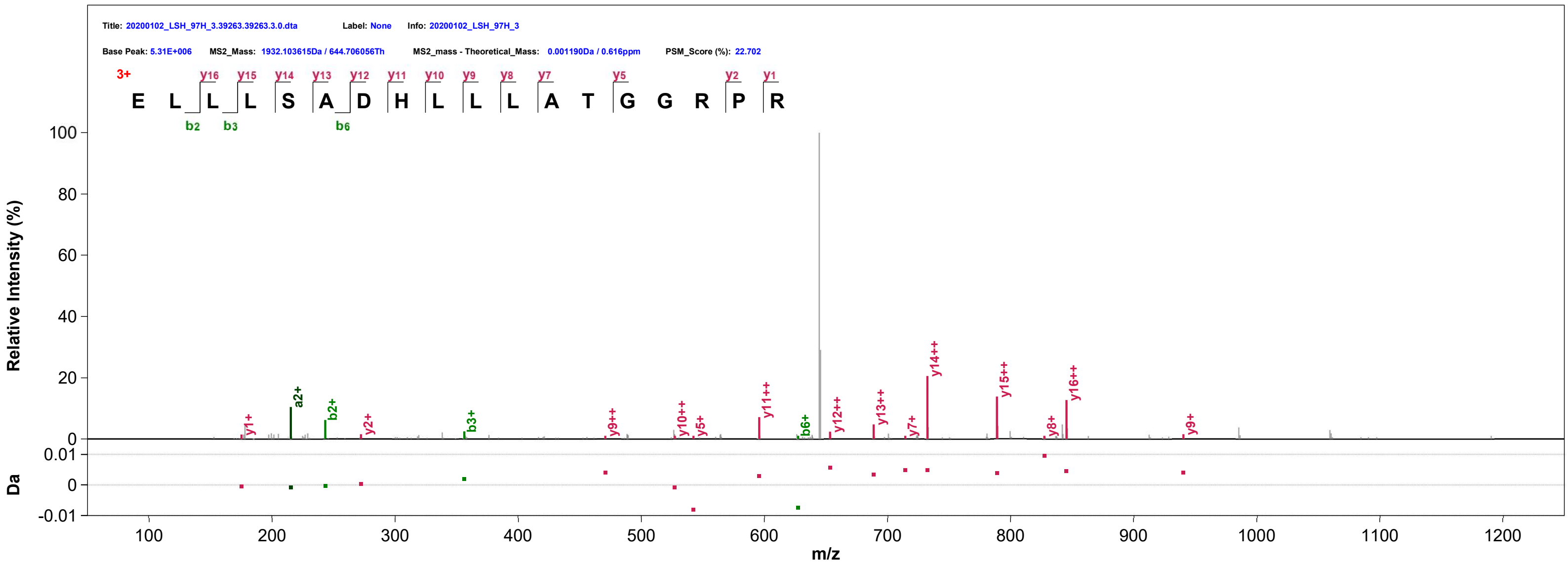

Protein Name: GAPDH_ORF_1
Peptide UUID: 86a335f1-d2b4-4d19-8f5b-dfdafabebc5d_GAPDH_ORF_1

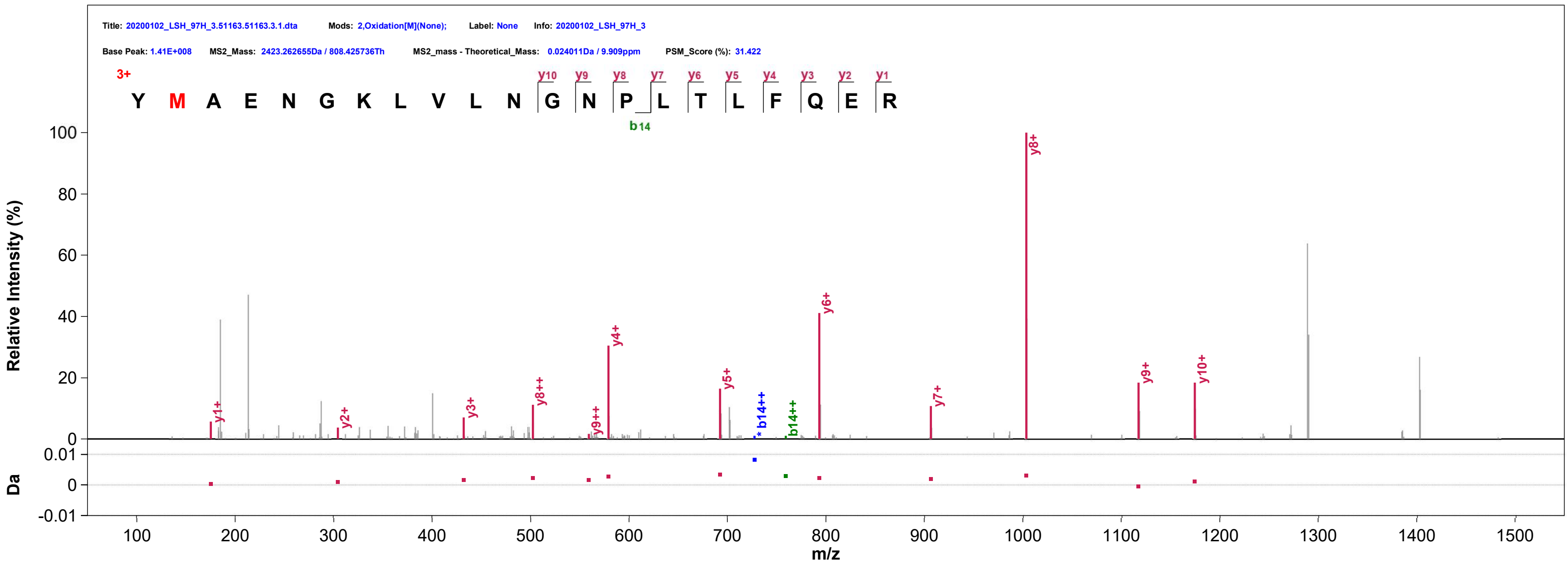

Protein Name: PON2_ORF_0
Peptide UUID: 8d50f3a4-726b-4d45-af4d-061cf0aecb81_PON2_ORF_0_1

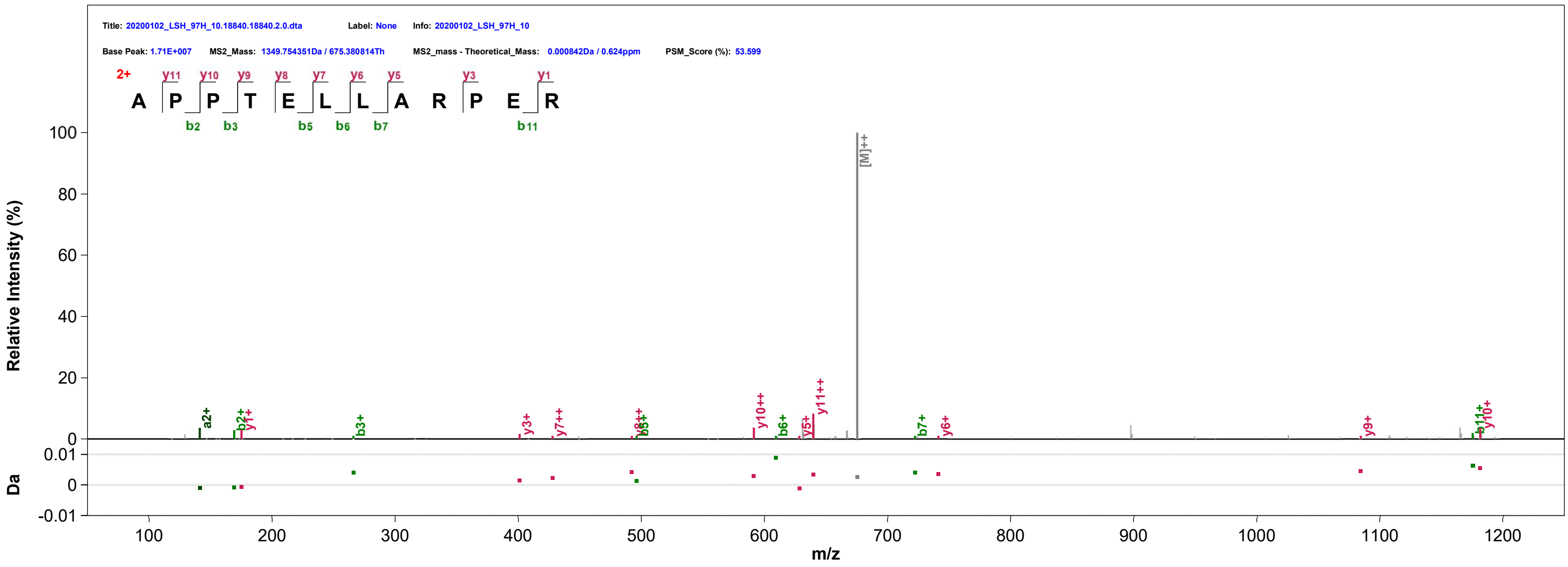

Protein Name: BPTF_ORF_1
Peptide UUID: 92cb1b0a-6b0b-4055-9d58-2e482dd78f8e_BPTF_ORF_1_2

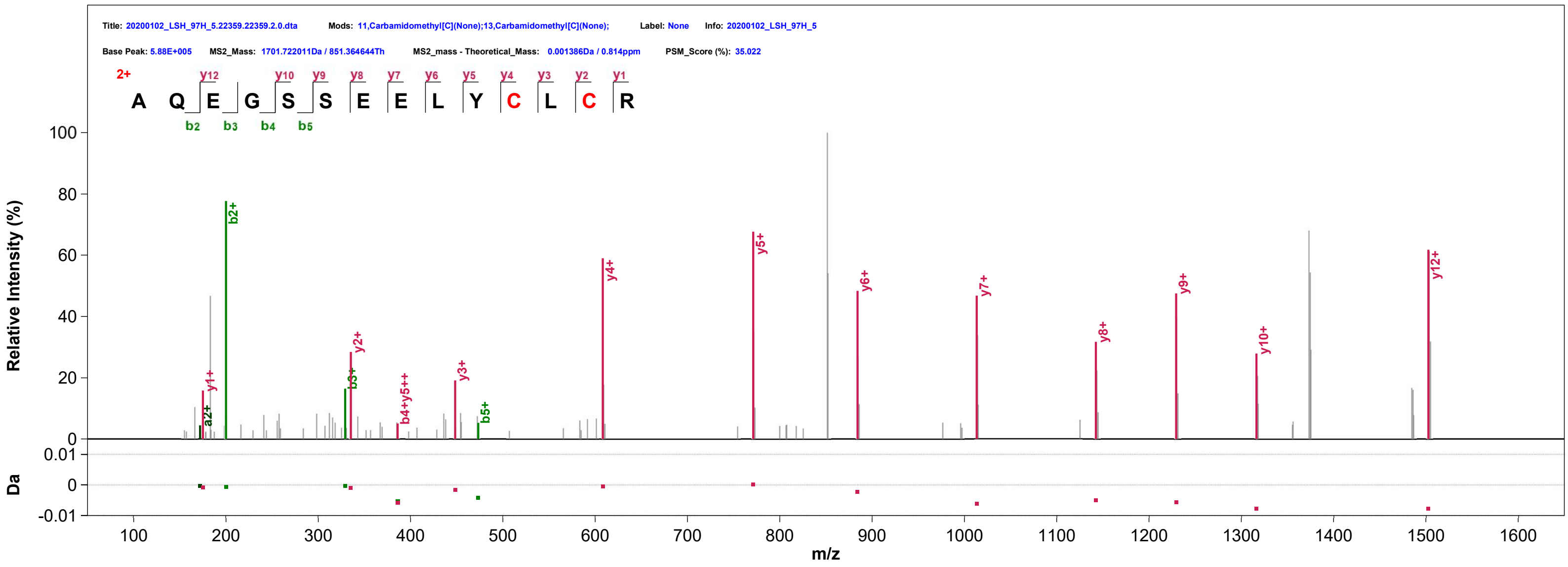

Protein Name: BPTF_ORF_1
Peptide UUID: 92cb1b0a-6b0b-4055-9d58-2e482dd78f8e_BPTF_ORF_1_4

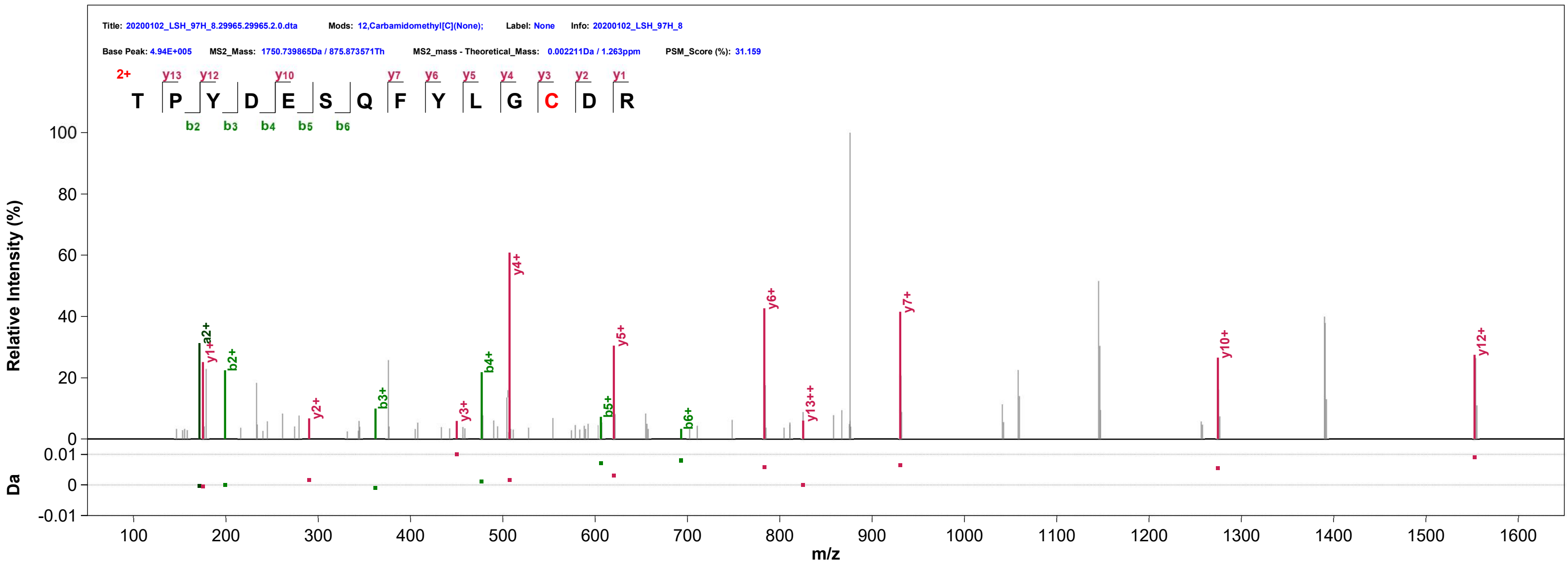

Protein Name: APOBEC3B_ORF_1
Peptide UUID: 96188e31-f11d-4a9b-8144-a3d7c073911a_APOBEC3B_ORF_1

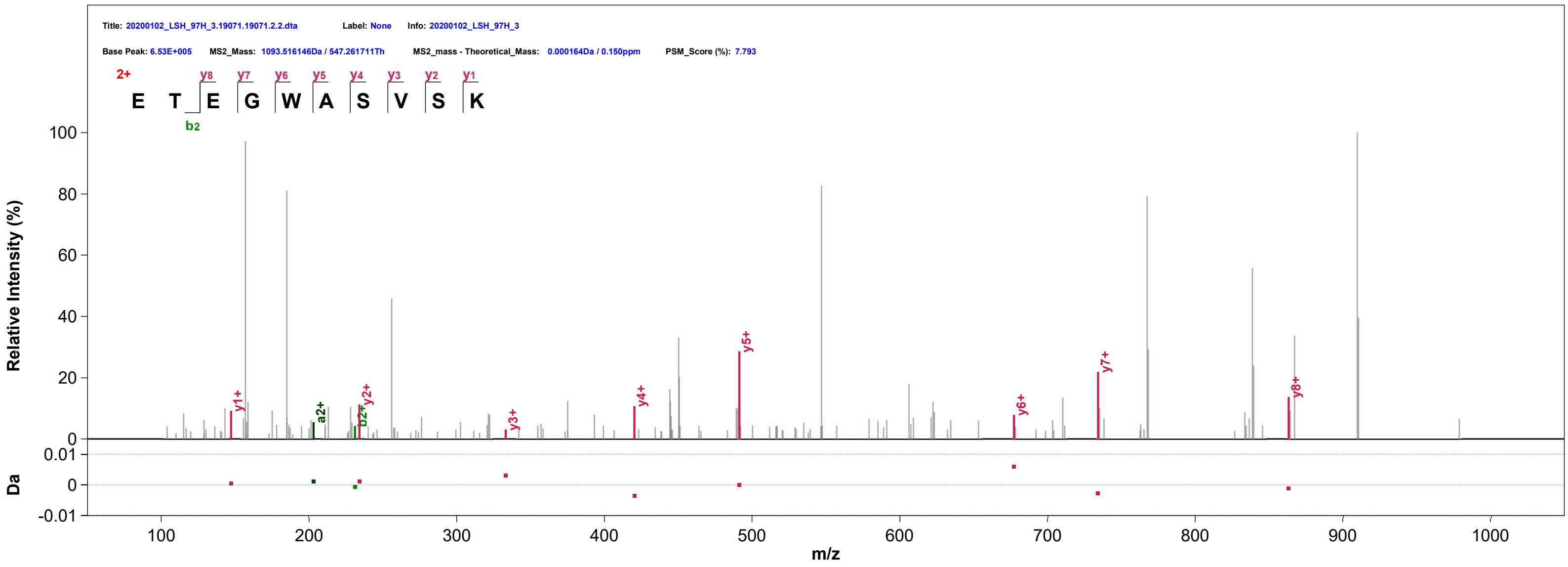

Protein Name: TPRXL_ORF_2
Peptide UUID: 97703fbe-fb1f-4e88-b8e1-7b2ccf09f094_TPRXL_ORF_2_1

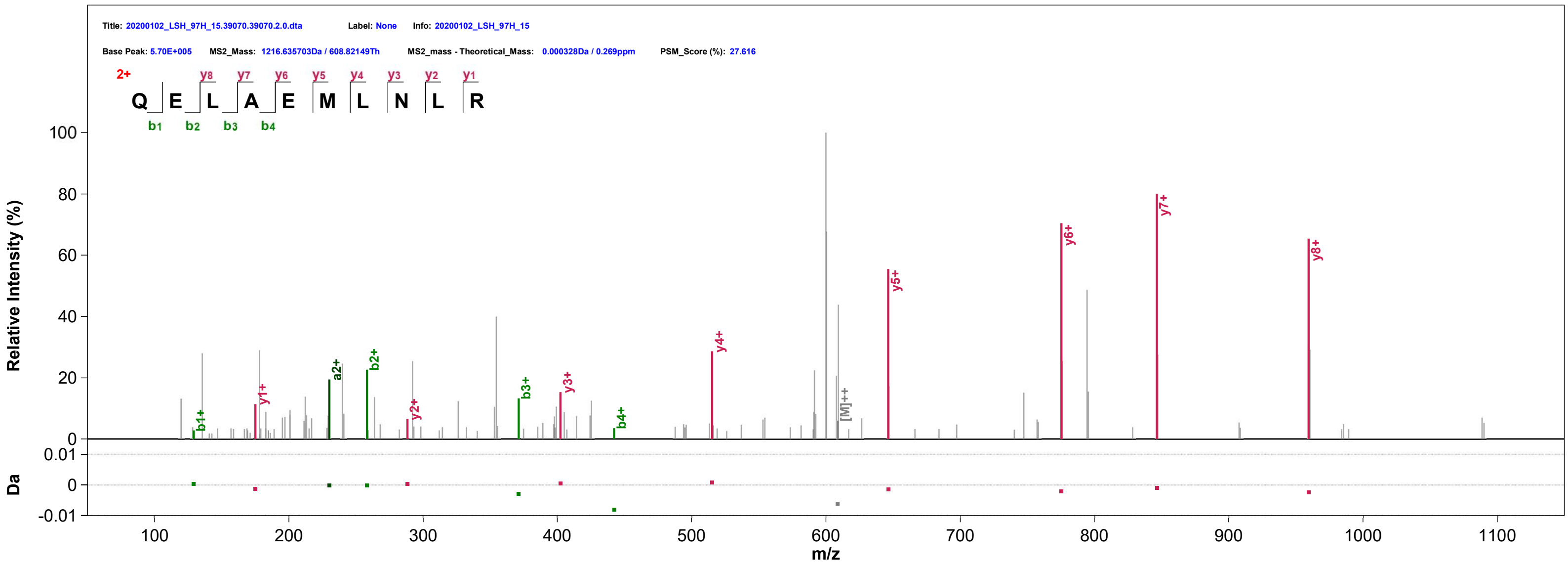

Protein Name: TPRXL_ORF_2
Peptide UUID: 97703fbe-fb1f-4e88-b8e1-7b2ccf09f094_TPRXL_ORF_2_3

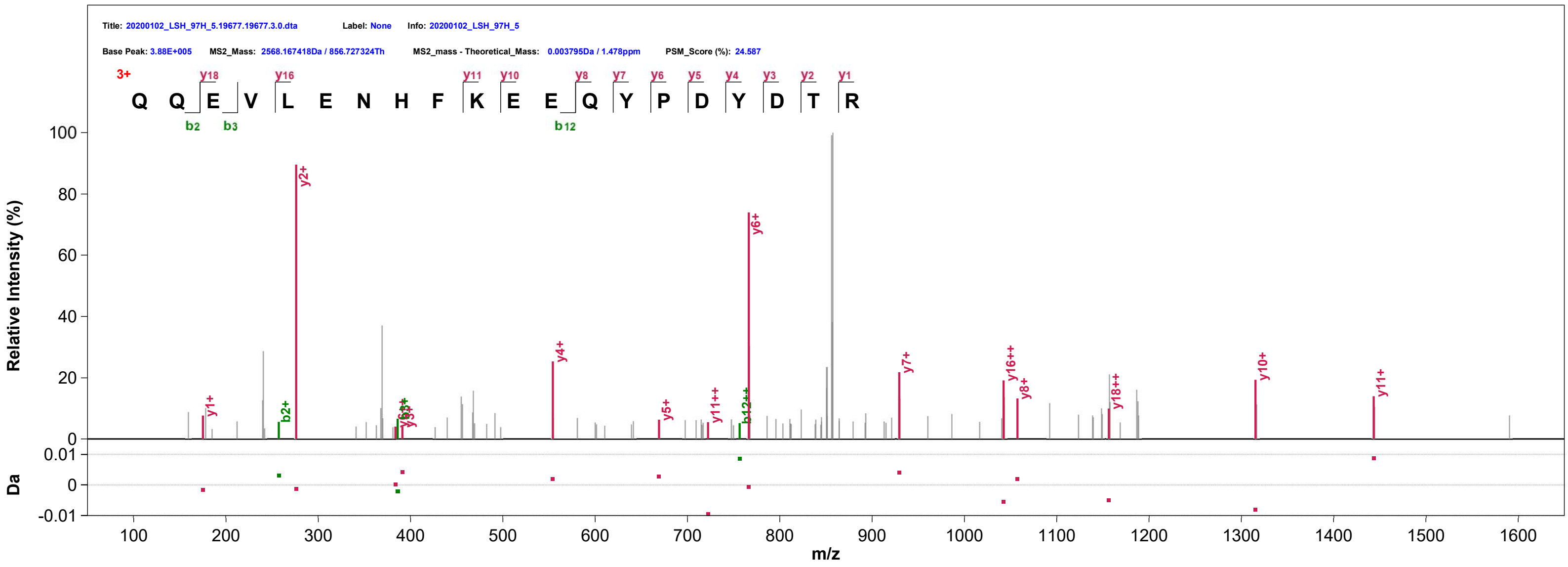

Protein Name: TPRXL_ORF_2
Peptide UUID: 97703fbe-fb1f-4e88-b8e1-7b2ccf09f094_TPRXL_ORF_2_4

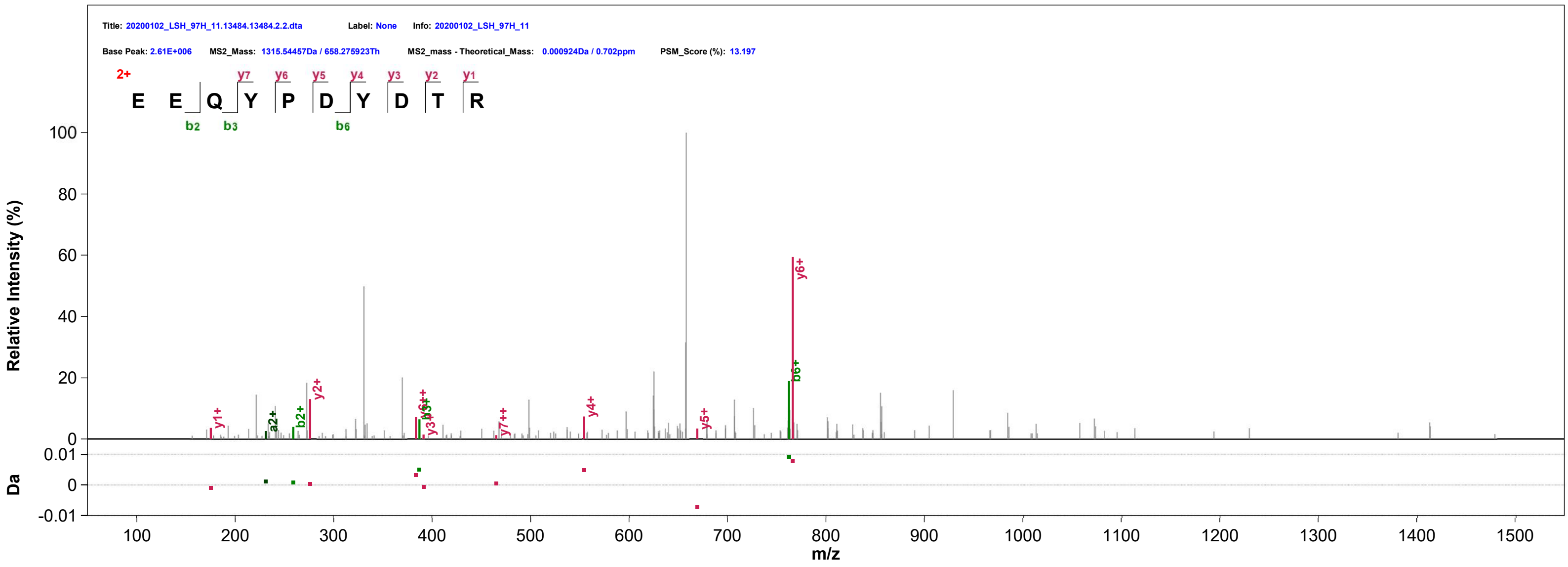

Protein Name: PSIP1_ORF_1
Peptide UUID: 9a89e8a2-6fea-41a7-8afa-b31a8353f23d_PSIP1_ORF_1_9

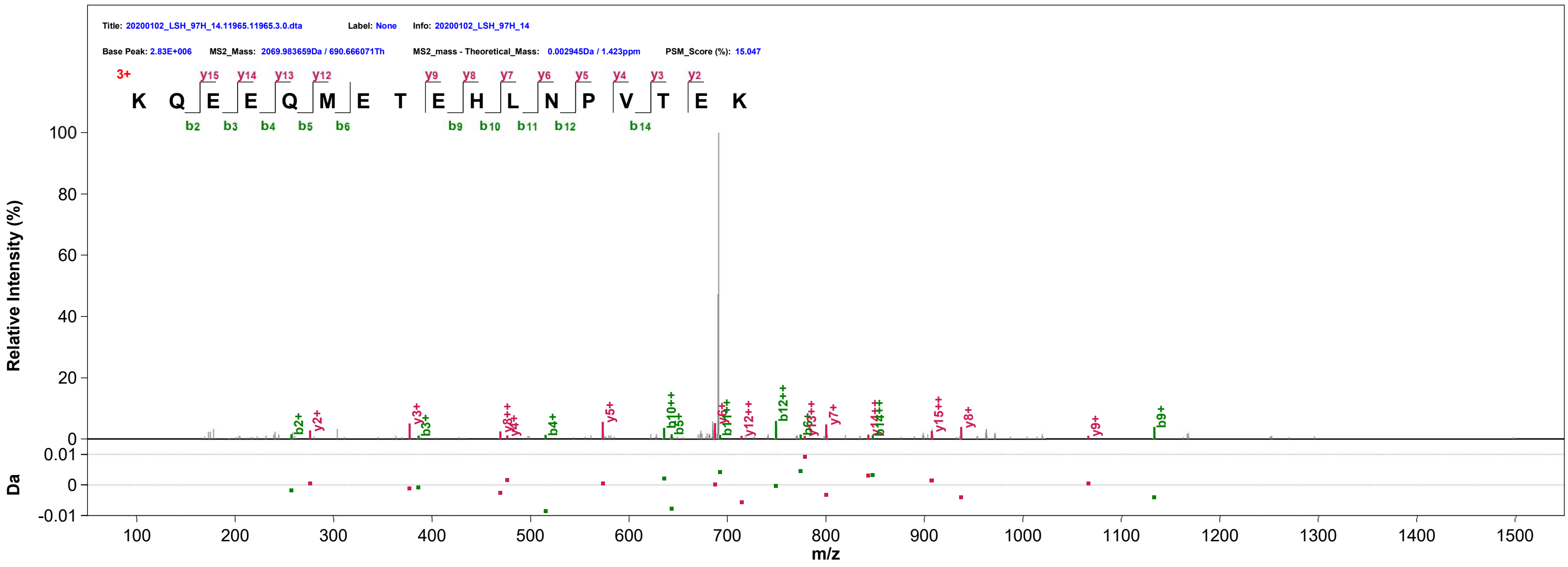

Protein Name: PDE4DIP_ORF_0
Peptide UUID: 9eb4687a-0921-4199-ad60-d62ccdf87ba2_PDE4DIP_ORF_0_8

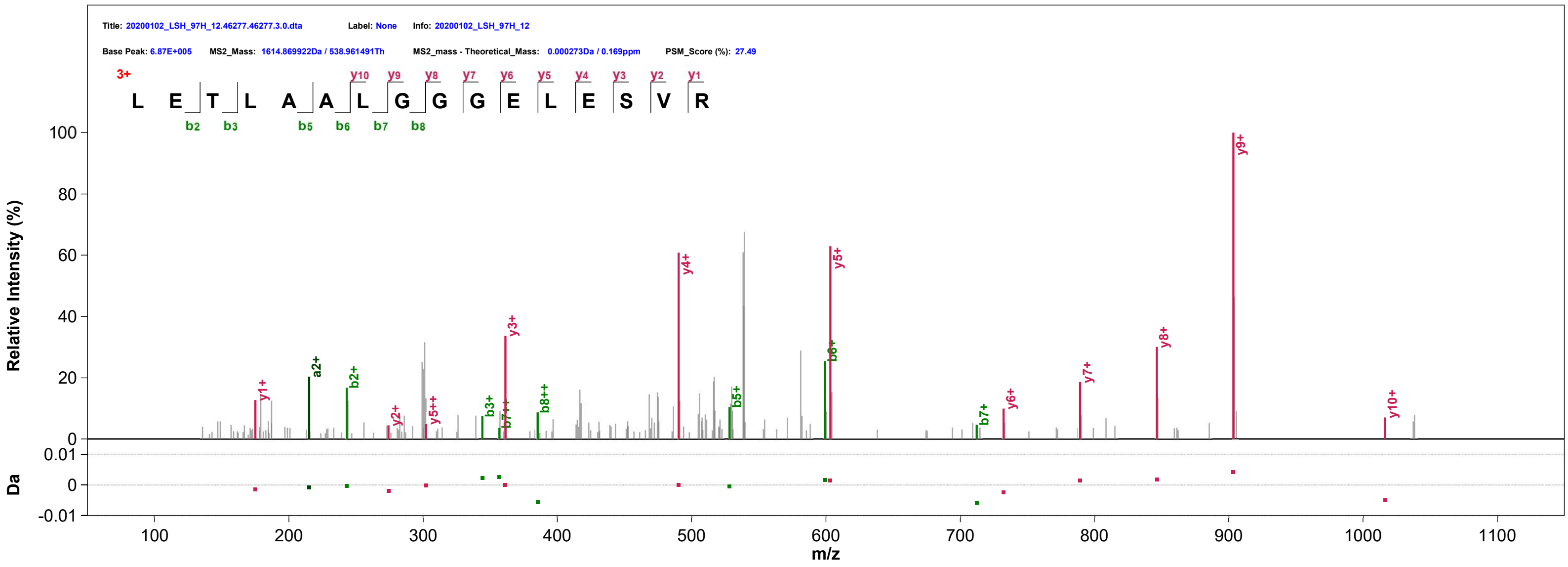

Protein Name: ELMSAN1_ORF_1
Peptide UUID: 9f580cff-ef97-4fc8-8dbd-93b2b9743816_ELMSAN1_ORF_1_4

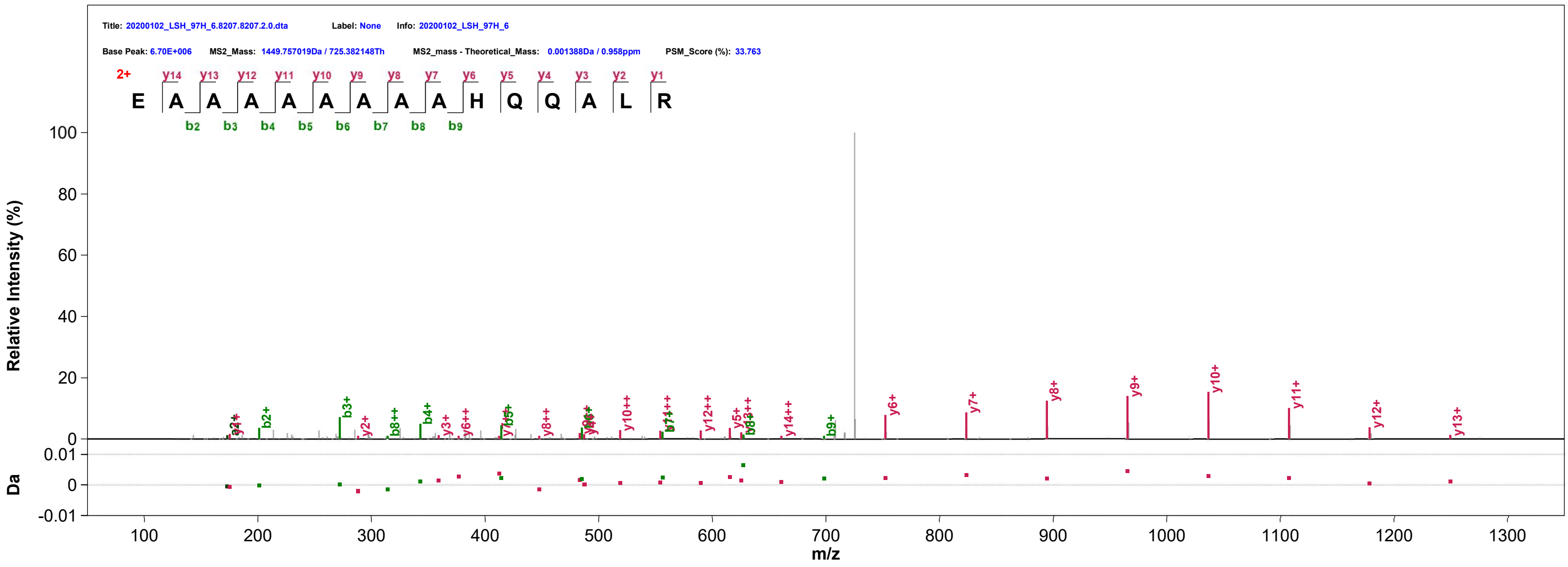

Protein Name: ASPH_ORF_0
Peptide UUID: a600aef5-f9ad-4ff8-b437-5fe0186e7b3a_ASPH_ORF_0

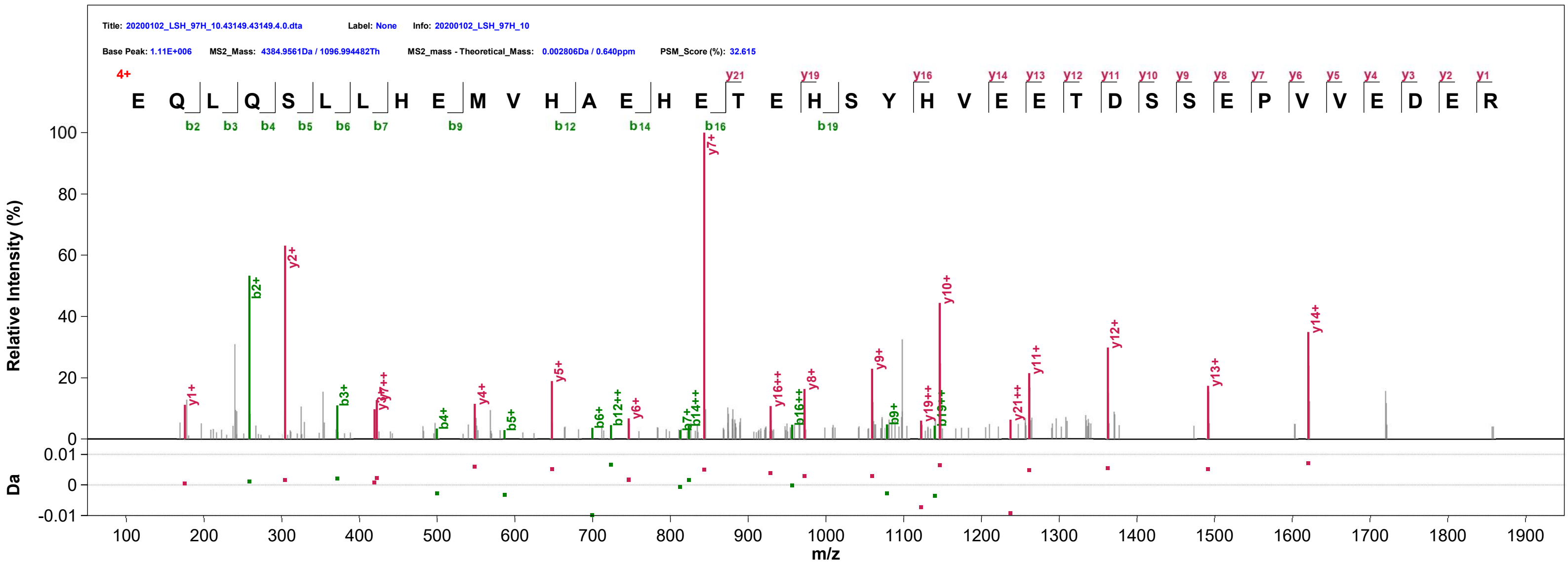

Protein Name: AP1M2_ORF_1
Peptide UUID: aefe2b91-834d-4662-afb4-483d999c75e5_AP1M2_ORF_1

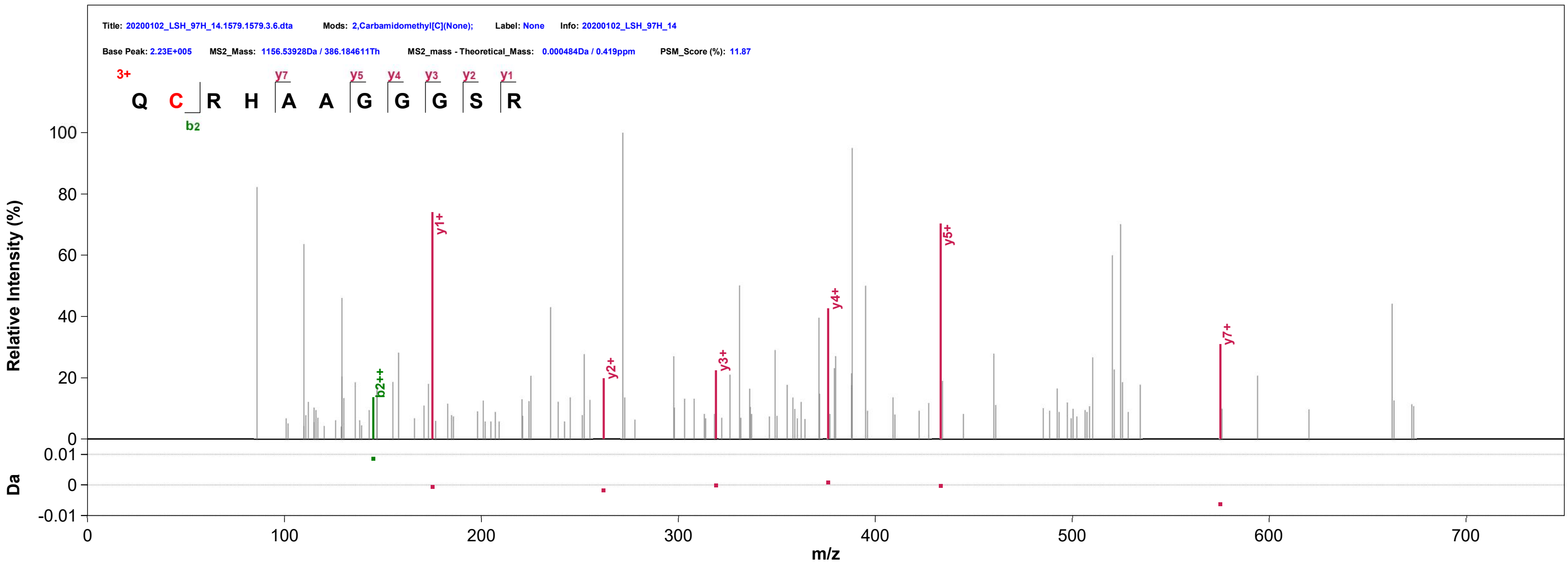

Protein Name: PRRC2C_ORF_1
Peptide UUID: b224e4b2-9d5f-4d57-8979-1183e1a00a2b_PRRC2C_ORF_1_37

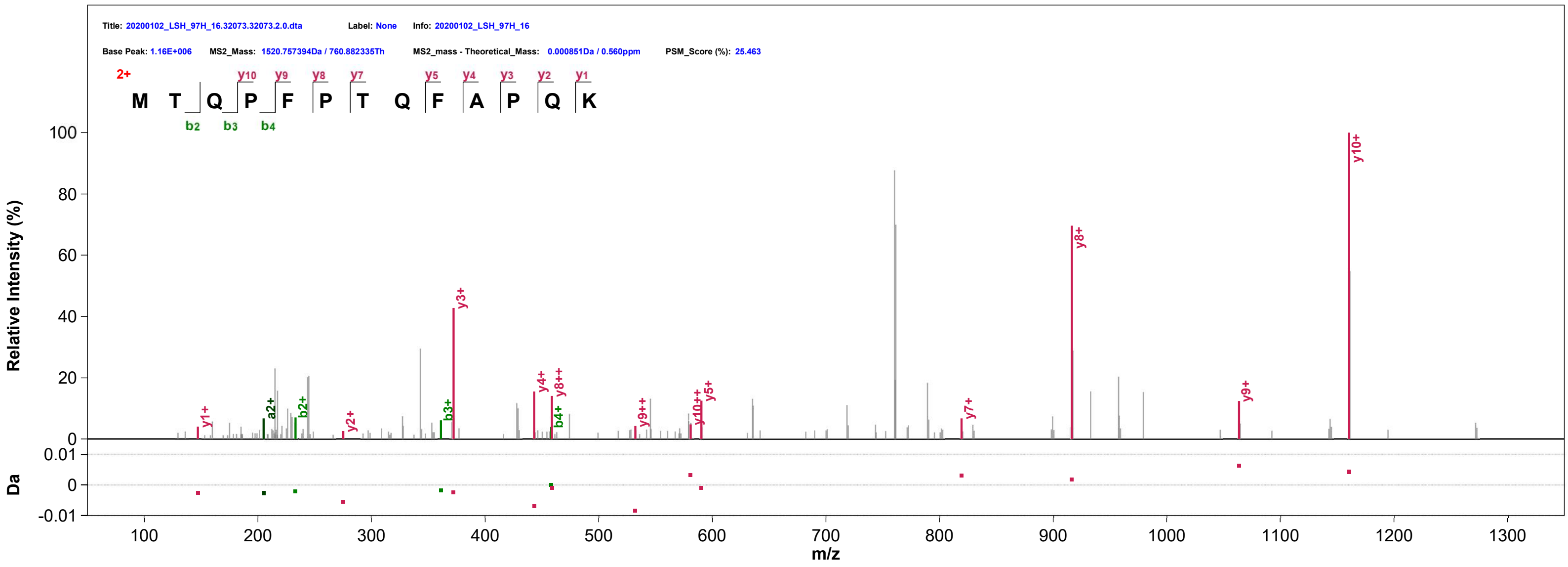

Protein Name: PCBP2_ORF_0
Peptide UUID: b830049d-ac27-4d19-b25f-331b420d547e_PCBP2_ORF_0_1

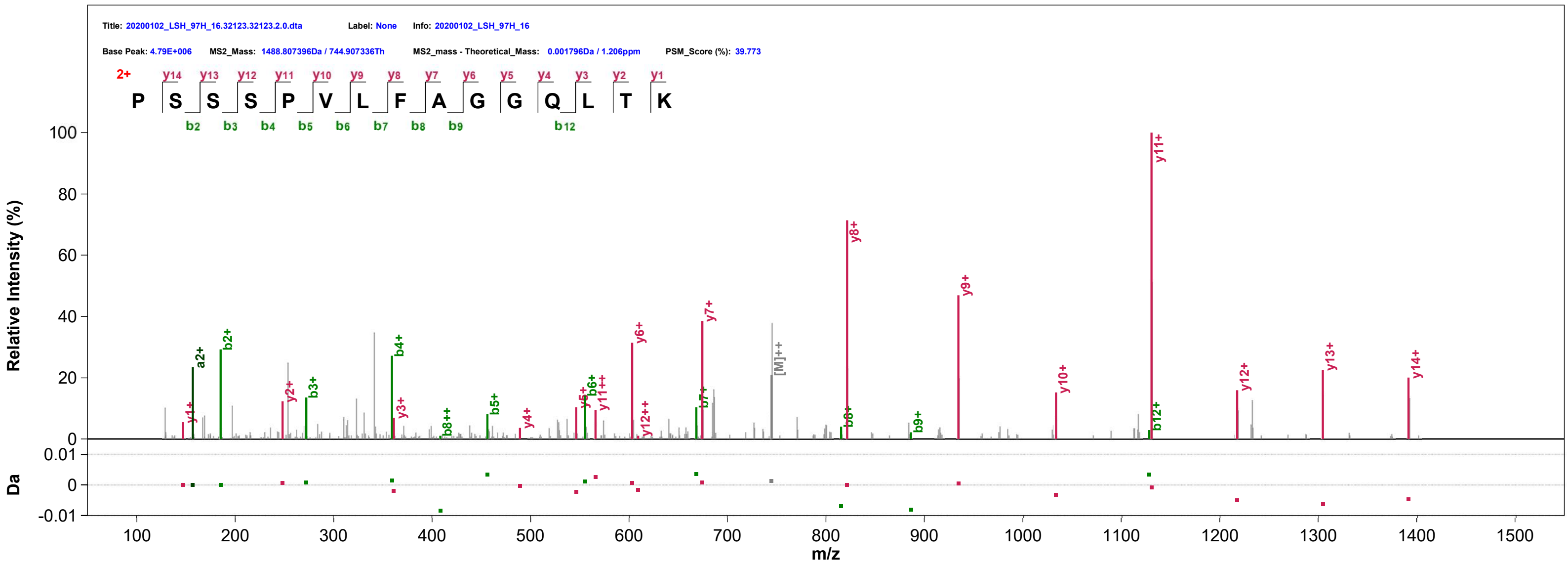

Protein Name: PCBP2_ORF_0
Peptide UUID: b830049d-ac27-4d19-b25f-331b420d547e_PCBP2_ORF_0_2

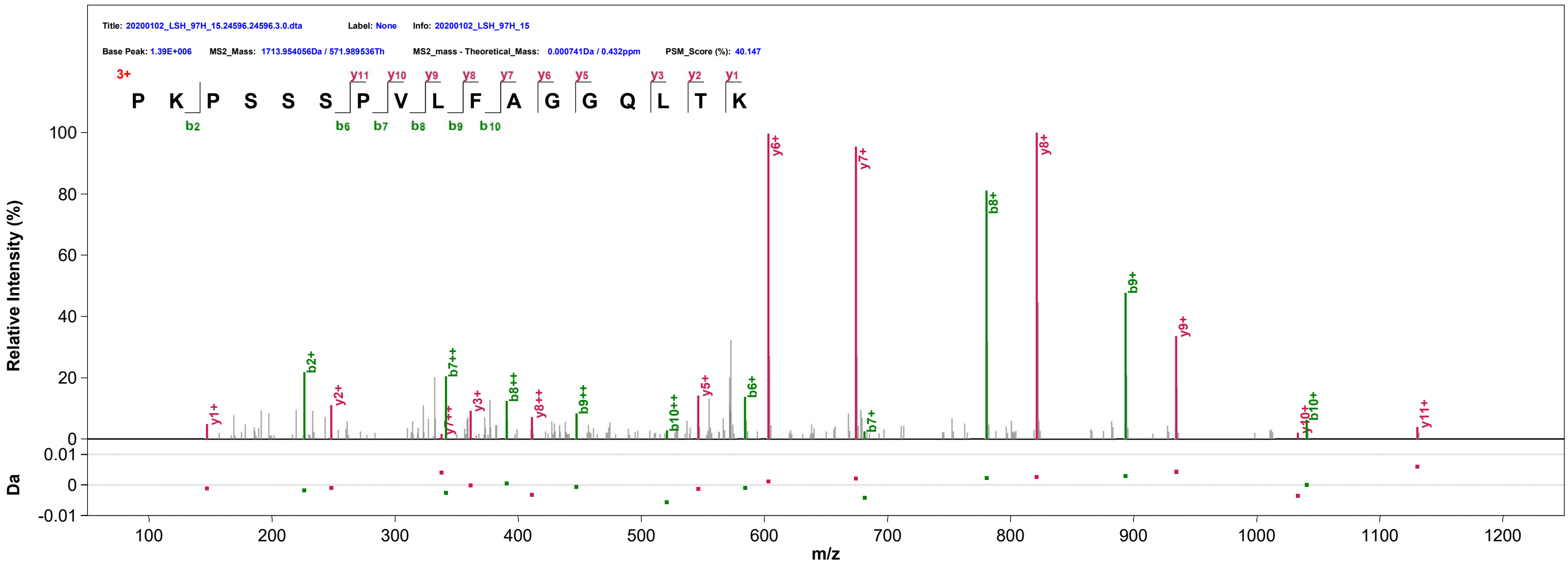

Protein Name: DNMT1_ORF_1
Peptide UUID: c08f210c-a7fa-4b40-acae-8fb2bf90a619_DNMT1_ORF_1

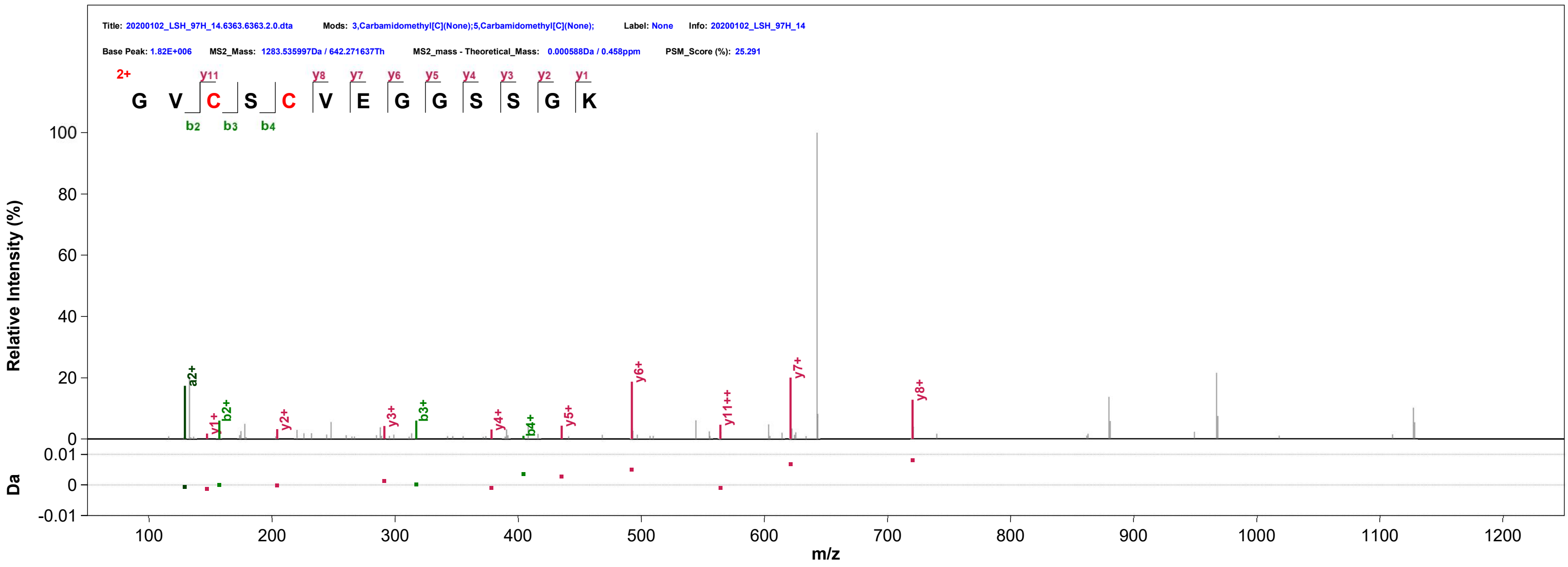

Protein Name: MACF1_ORF_0
Peptide UUID: c313ece0-48f6-4d66-a152-210c67b5a688_MACF1_ORF_0_20

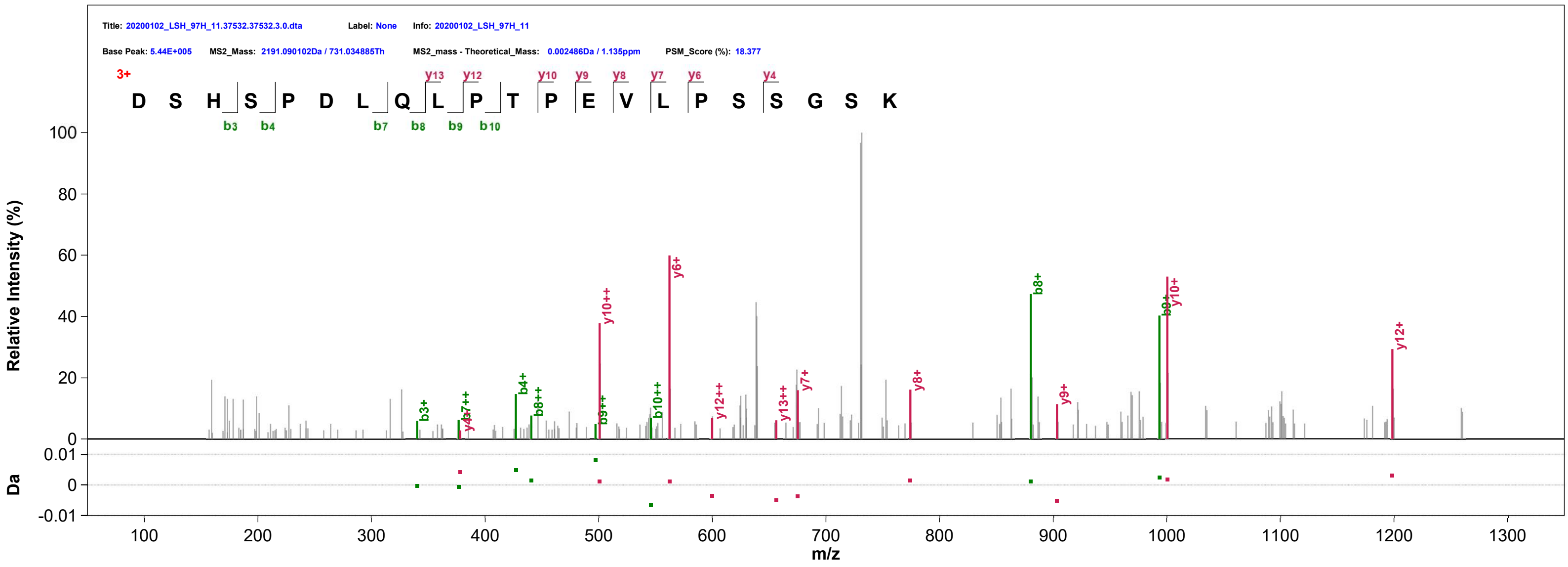

Protein Name: HNRNPAB_ORF_2
Peptide UUID: c32a27bd-316e-4bbb-b639-7733640591e2_HNRNPAB_ORF_2

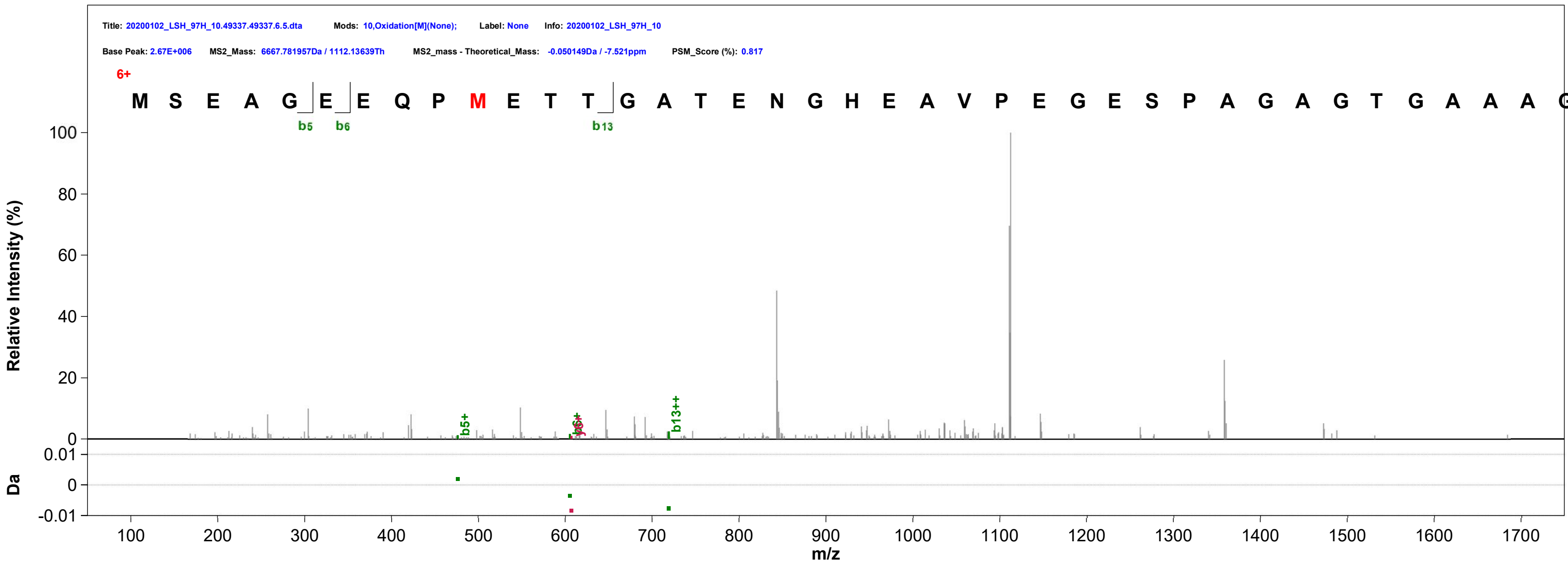

Protein Name: EEF1AKMT2_ORF_0
Peptide UUID: c45e0084-349c-4db7-bcae-1d620355efaa_EEF1AKMT2_ORF_0_2

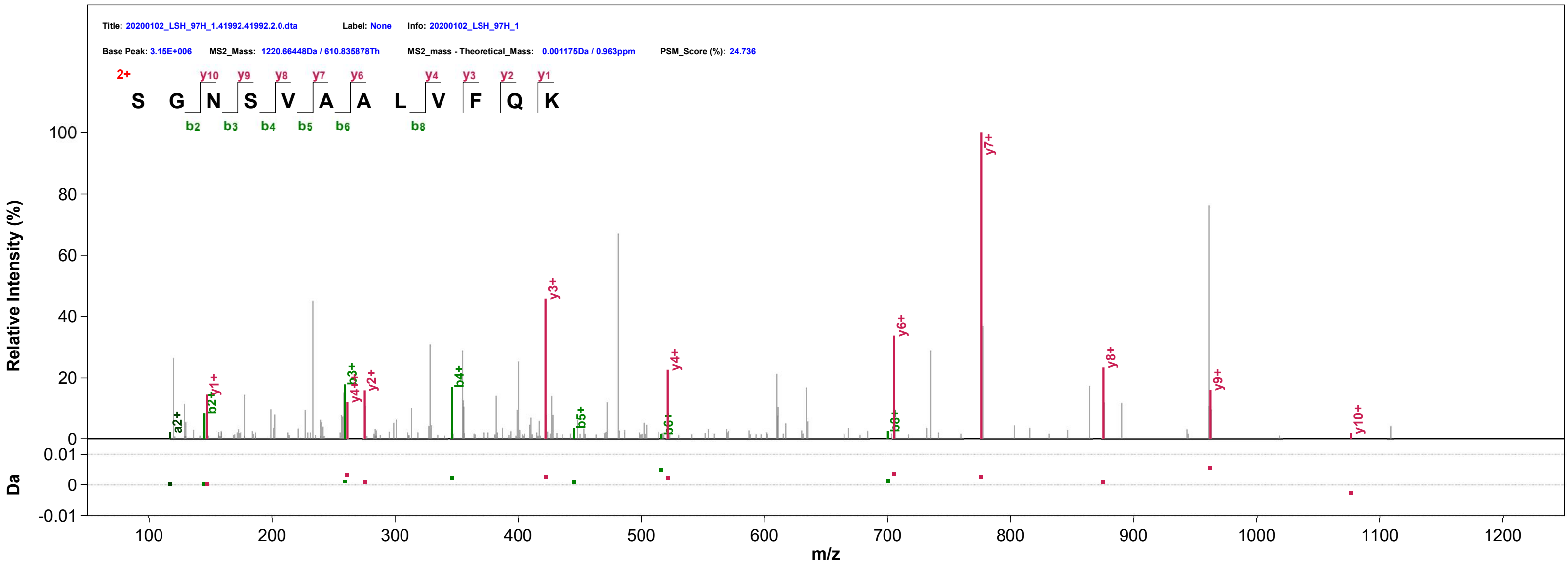

Protein Name: FLNA_ORF_1
Peptide UUID: d3ca5b5c-4266-41c5-b34c-595b8b545cdb_FLNA_ORF_1

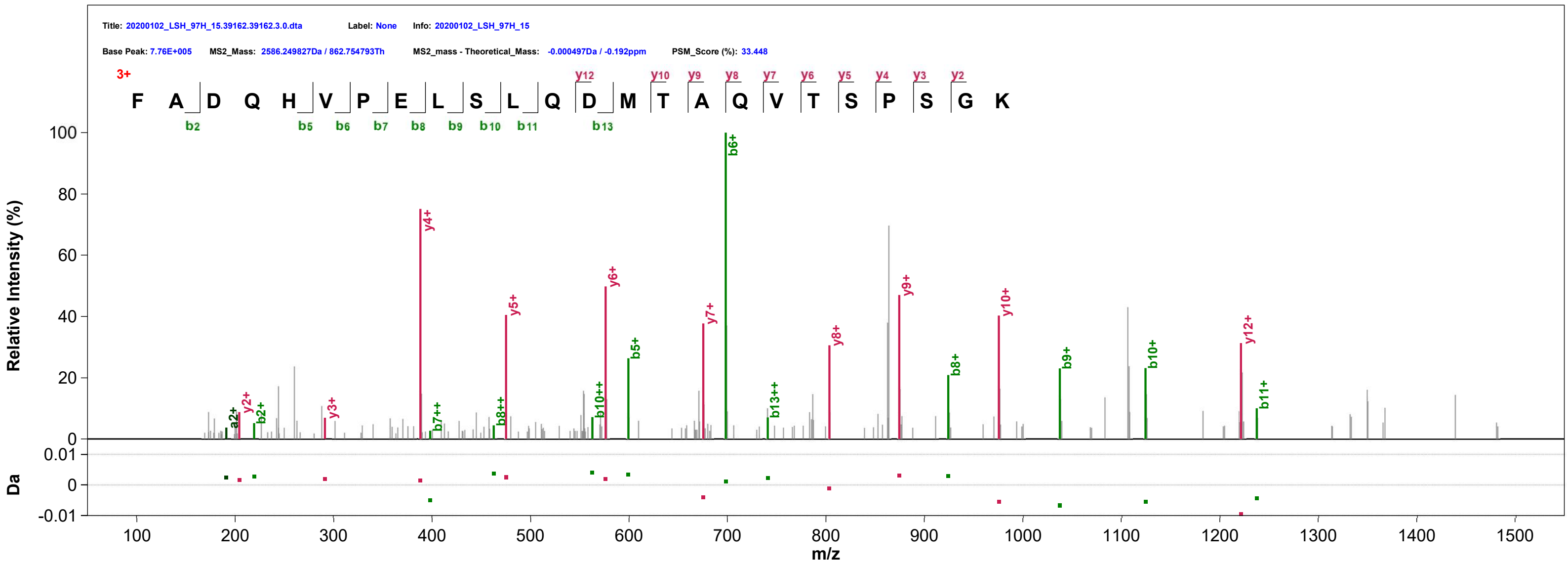

Protein Name: MET_ORF_0
Peptide UUID: e4dd6293-cbc8-4f6a-94ad-e8428ab33492_MET_ORF_0

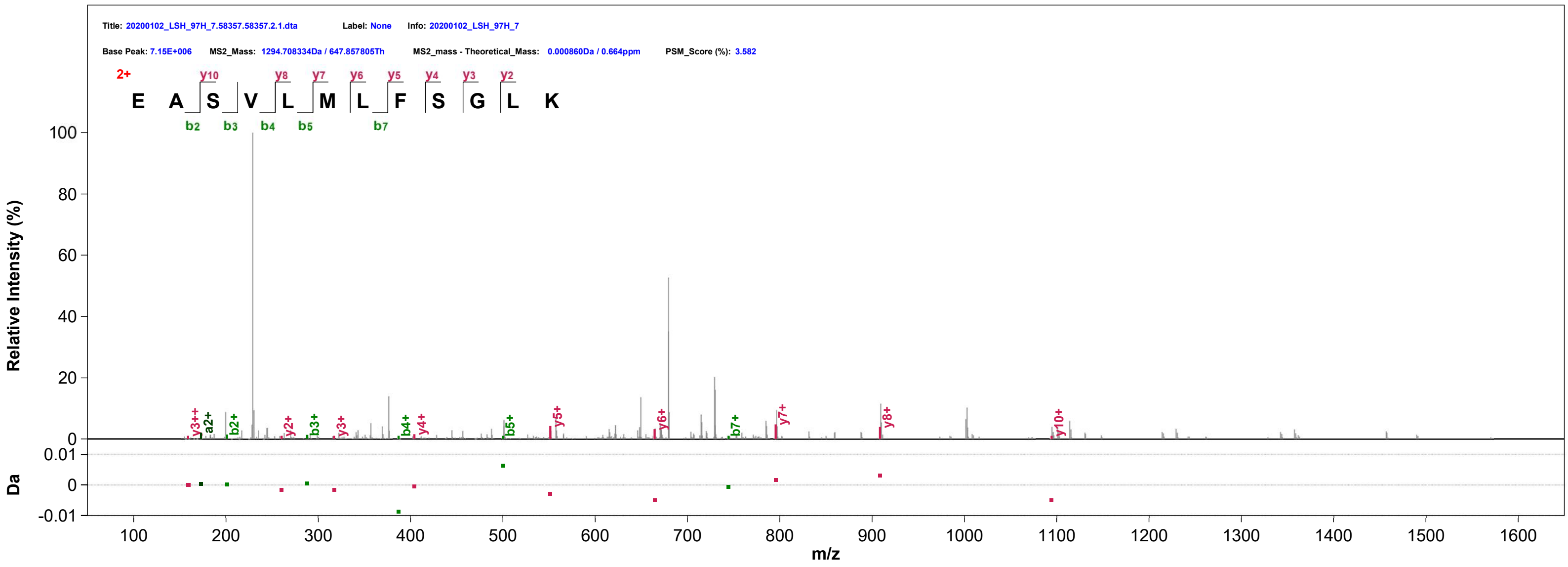

Protein Name: FARP2_ORF_0
Peptide UUID: e6426579-d099-4f68-b5c6-351bfd07ec53_FARP2_ORF_0_1

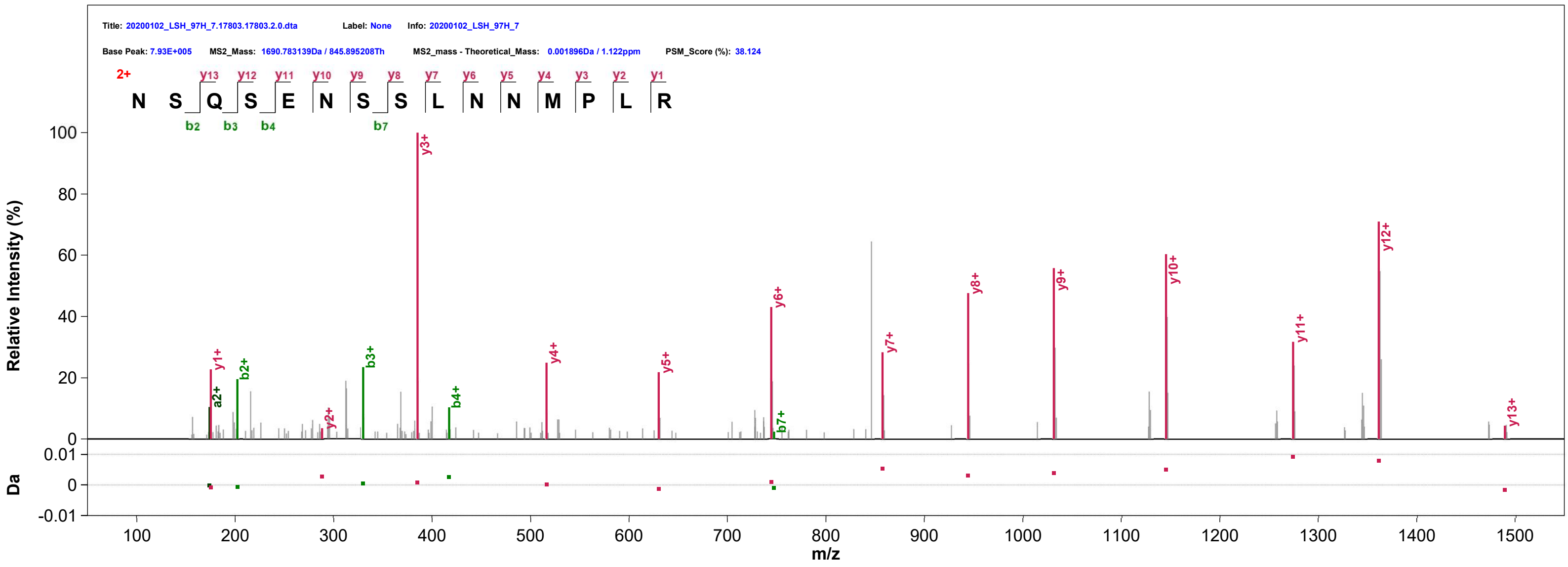

Protein Name: FARP2_ORF_0
Peptide UUID: e6426579-d099-4f68-b5c6-351bfd07ec53_FARP2_ORF_0_11

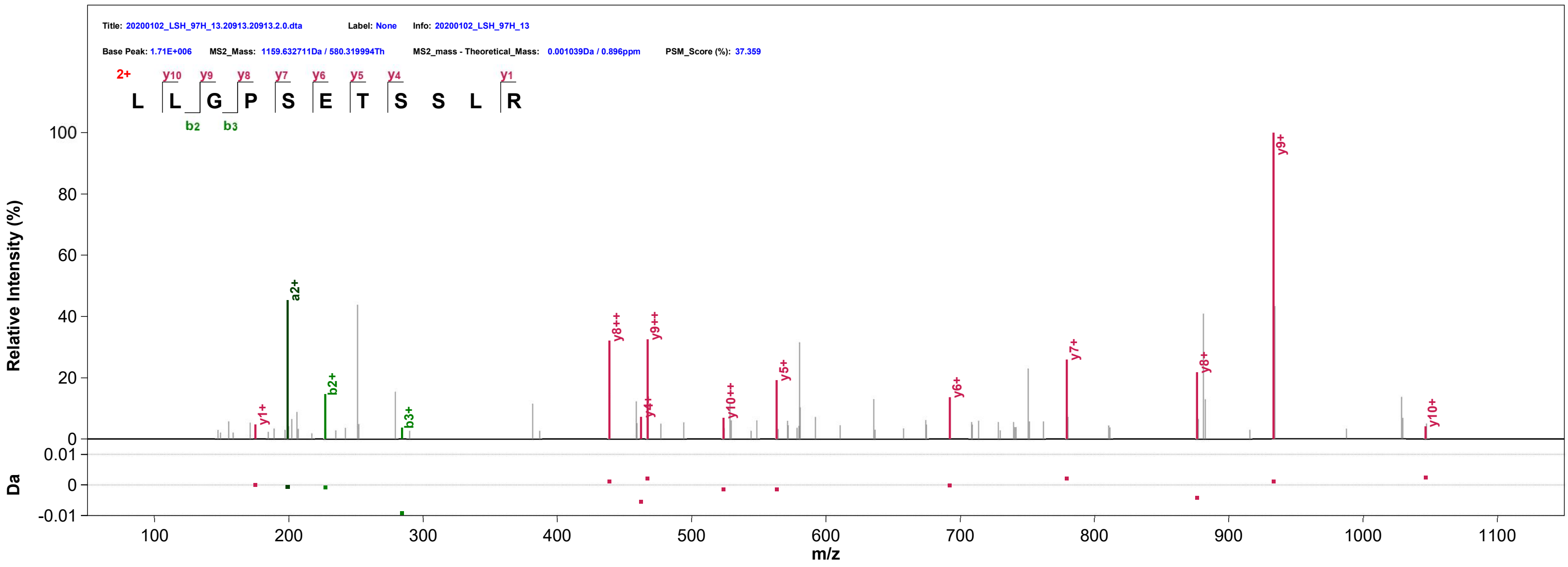

Protein Name: FARP2_ORF_0
Peptide UUID: e6426579-d099-4f68-b5c6-351bfd07ec53_FARP2_ORF_0_14

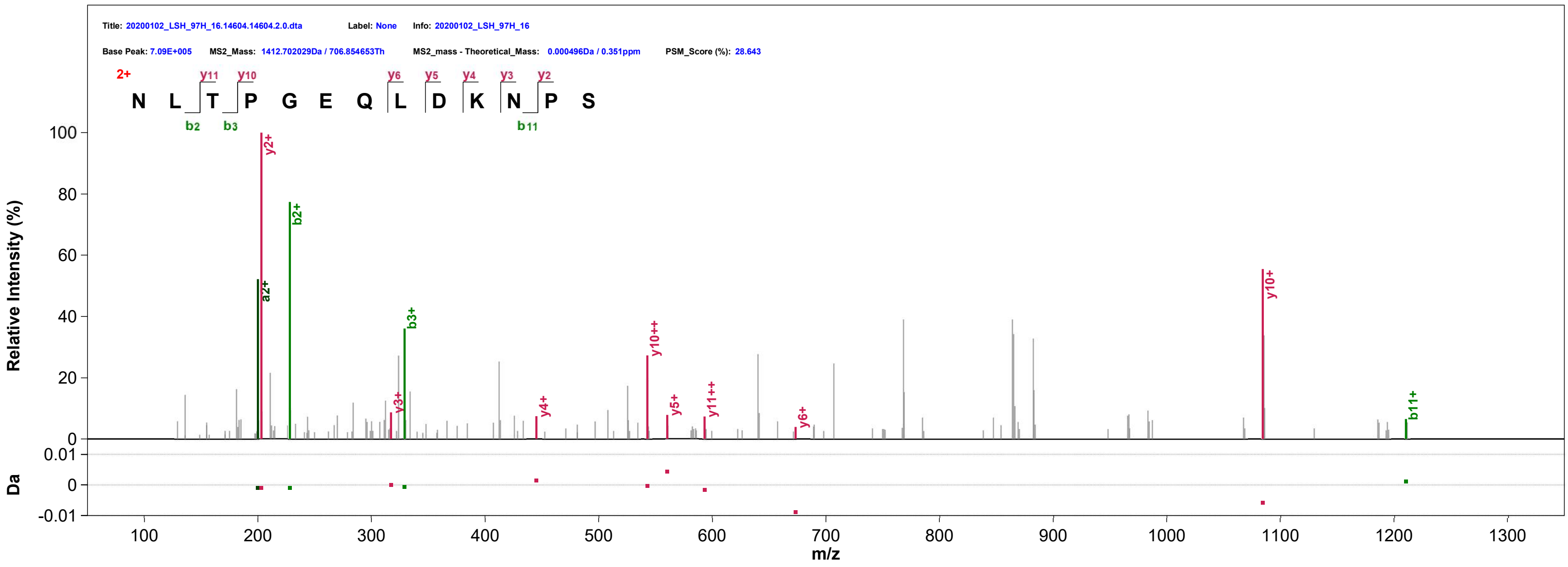

Protein Name: PCBP2_ORF_1
Peptide UUID: e65fee4b-8fcd-4f46-894e-aa6e2b906556_PCBP2_ORF_1

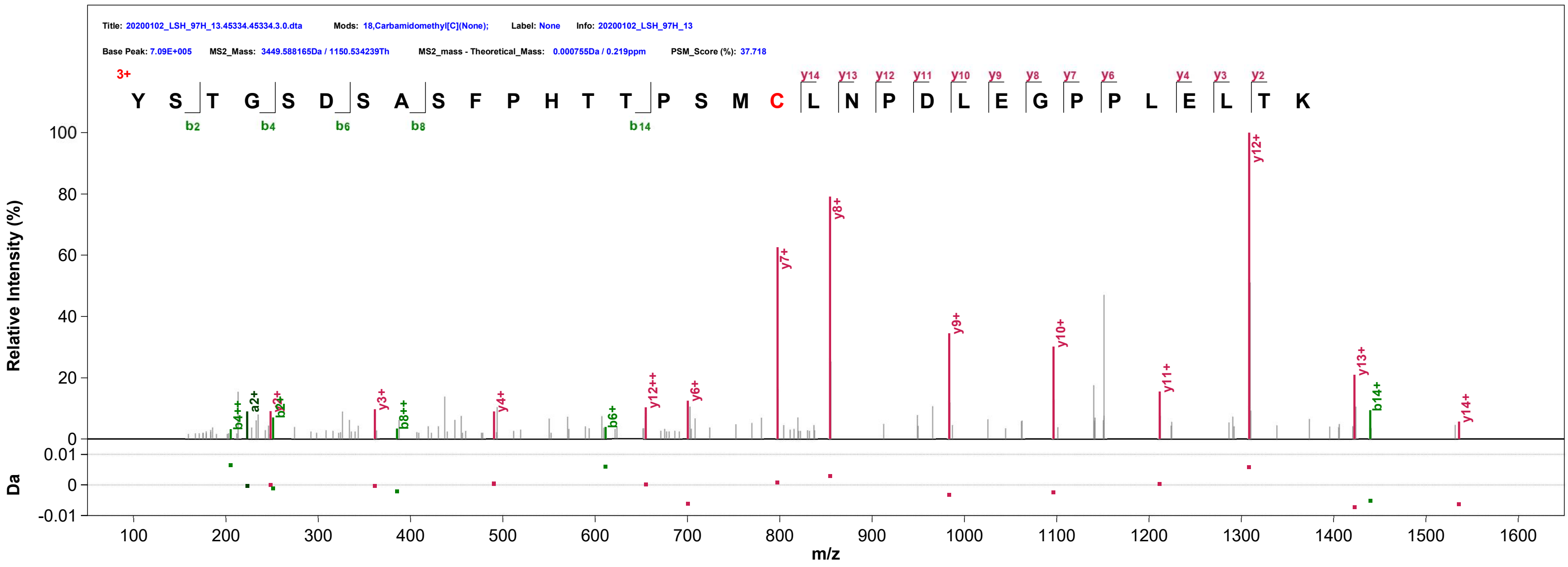

Protein Name: CDV3_ORF_0
Peptide UUID: e6e008d3-0620-40c7-9c6d-58b5f0f6c2cd_CDV3_ORF_0_1

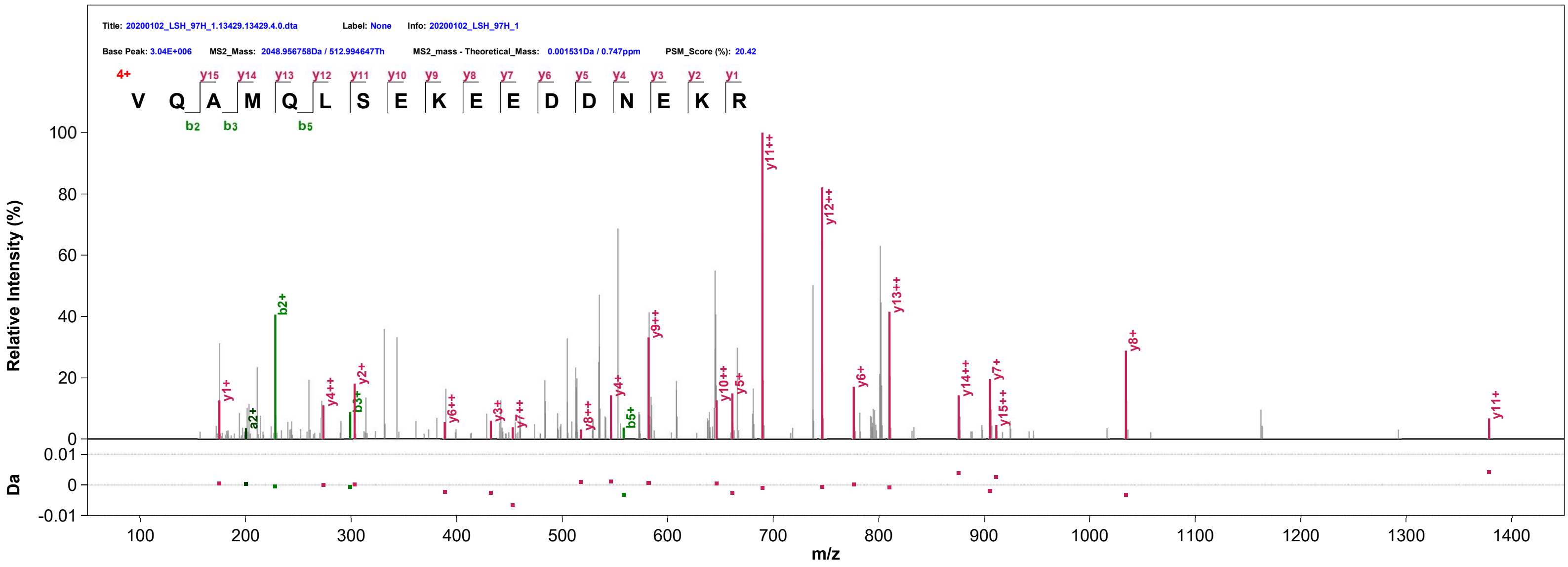

Protein Name: CDV3_ORF_0
Peptide UUID: e6e008d3-0620-40c7-9c6d-58b5f0f6c2cd_CDV3_ORF_0_2

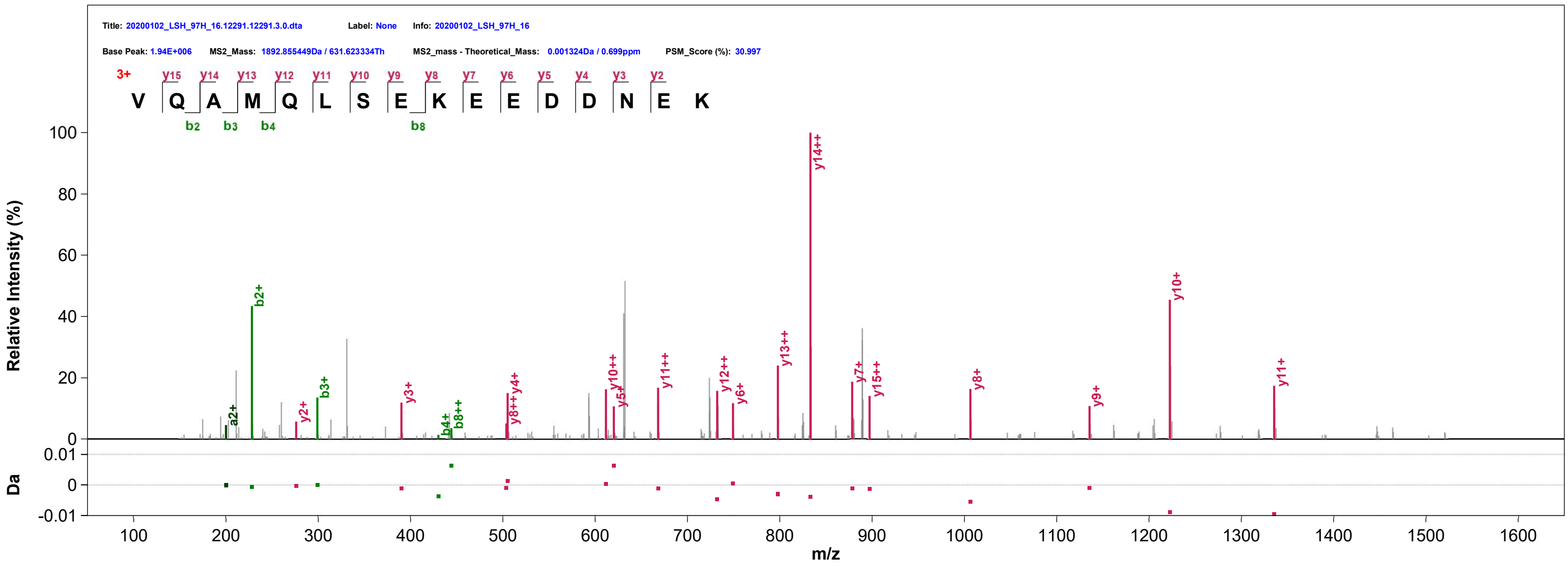

Protein Name: PLEKHA7_ORF_0
Peptide UUID: e6eee168-608d-4ee1-87d0-23599764a4bf_PLEKHA7_ORF_0_1

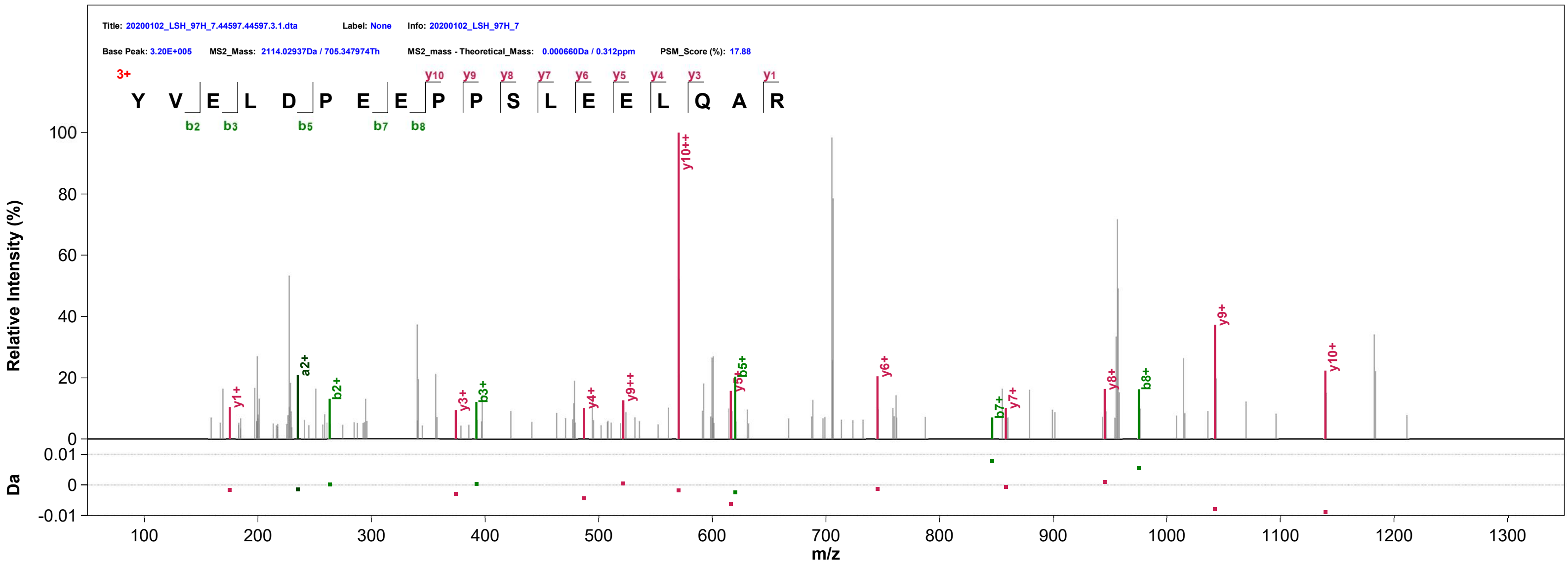

Protein Name: PLEKHA7_ORF_0
Peptide UUID: e6eee168-608d-4ee1-87d0-23599764a4bf_PLEKHA7_ORF_0_2

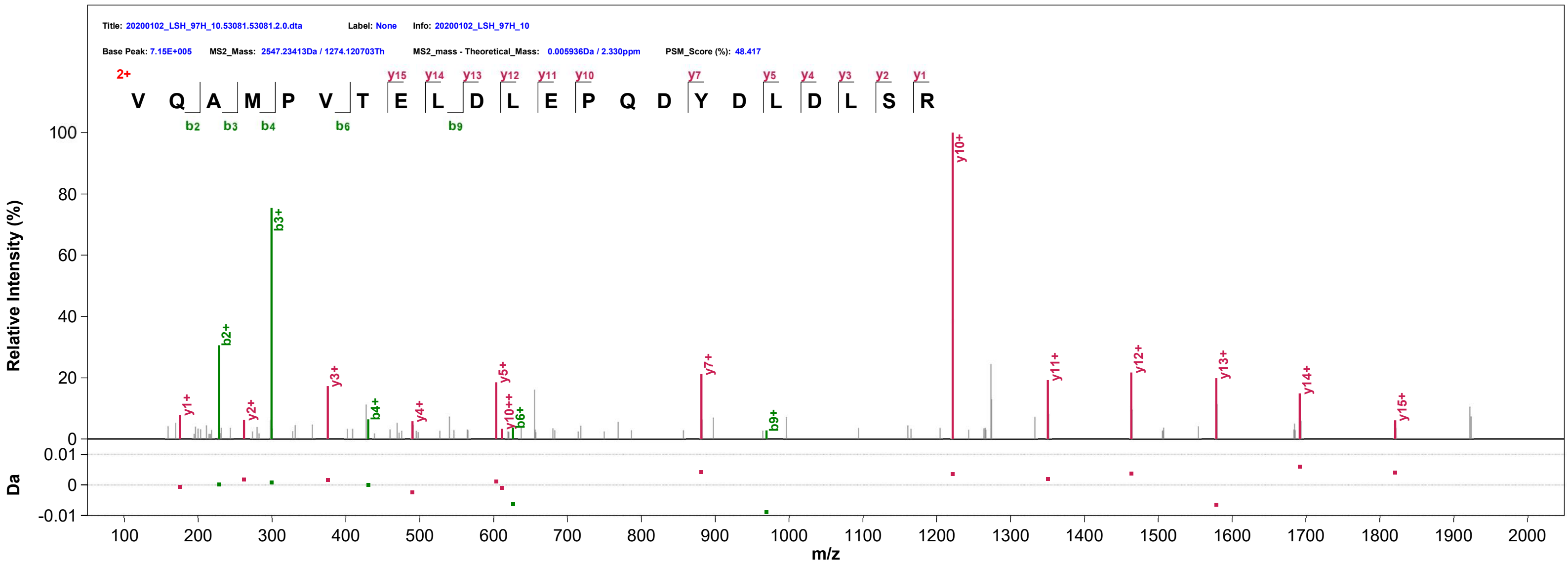

Protein Name: HUWE1_ORF_0
Peptide UUID: ef15e69e-73b7-4eb3-b8ed-c853e03d1389_HUWE1_ORF_0_4

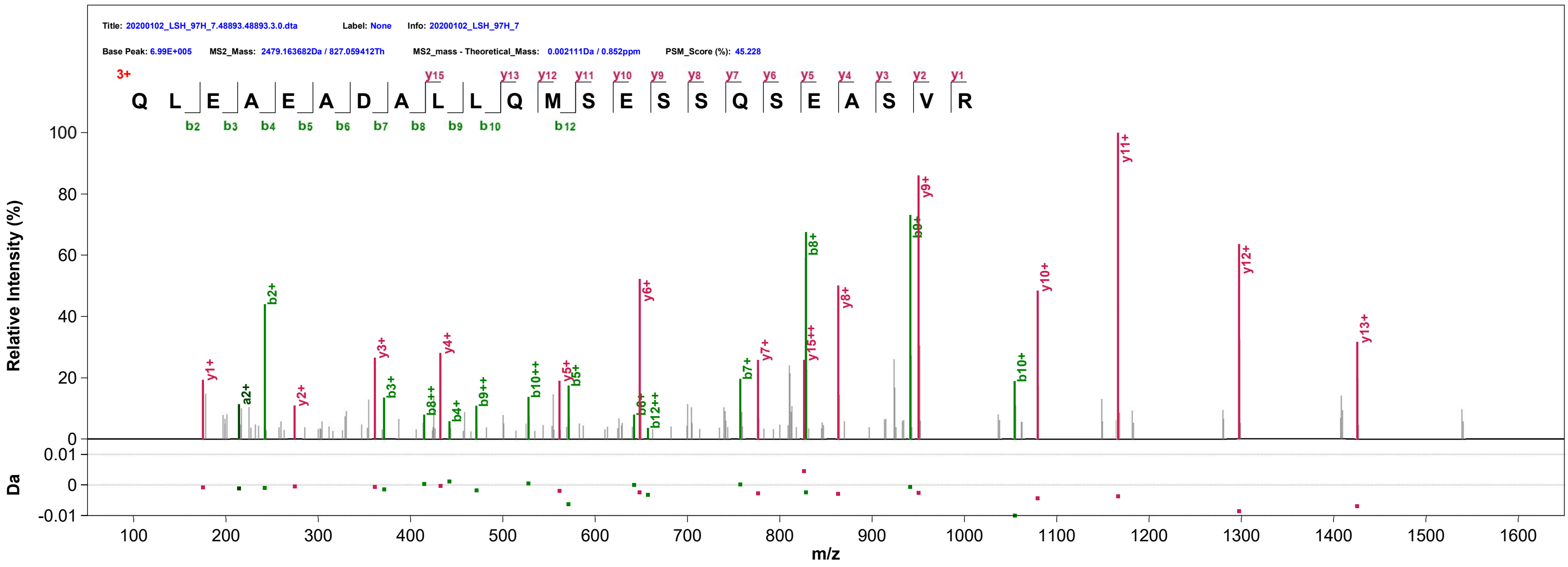

Protein Name: CHD3_ORF_1
Peptide UUID: f13a62d3-52fa-4ae9-80e0-d3a776ce85d9_CHD3_ORF_1_1

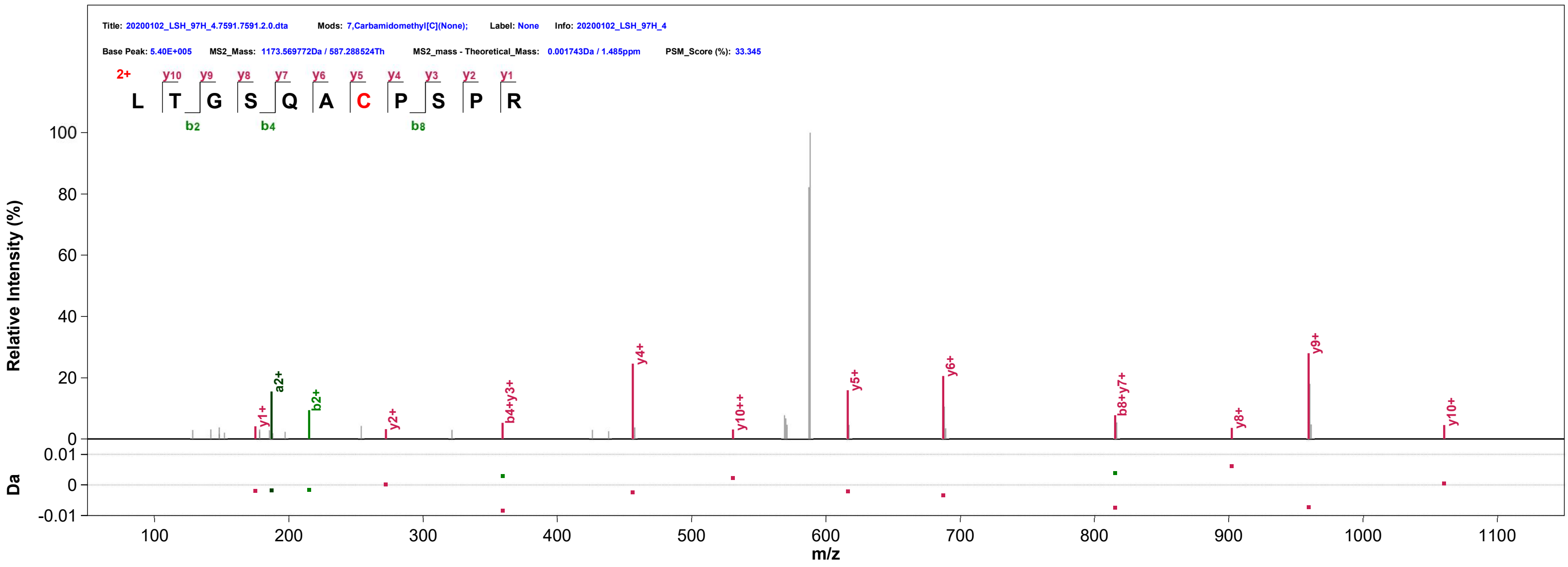

Protein Name: TES_ORF_0
Peptide UUID: f341d830-b131-41f4-84ad-b36831ca0d04_TES_ORF_0

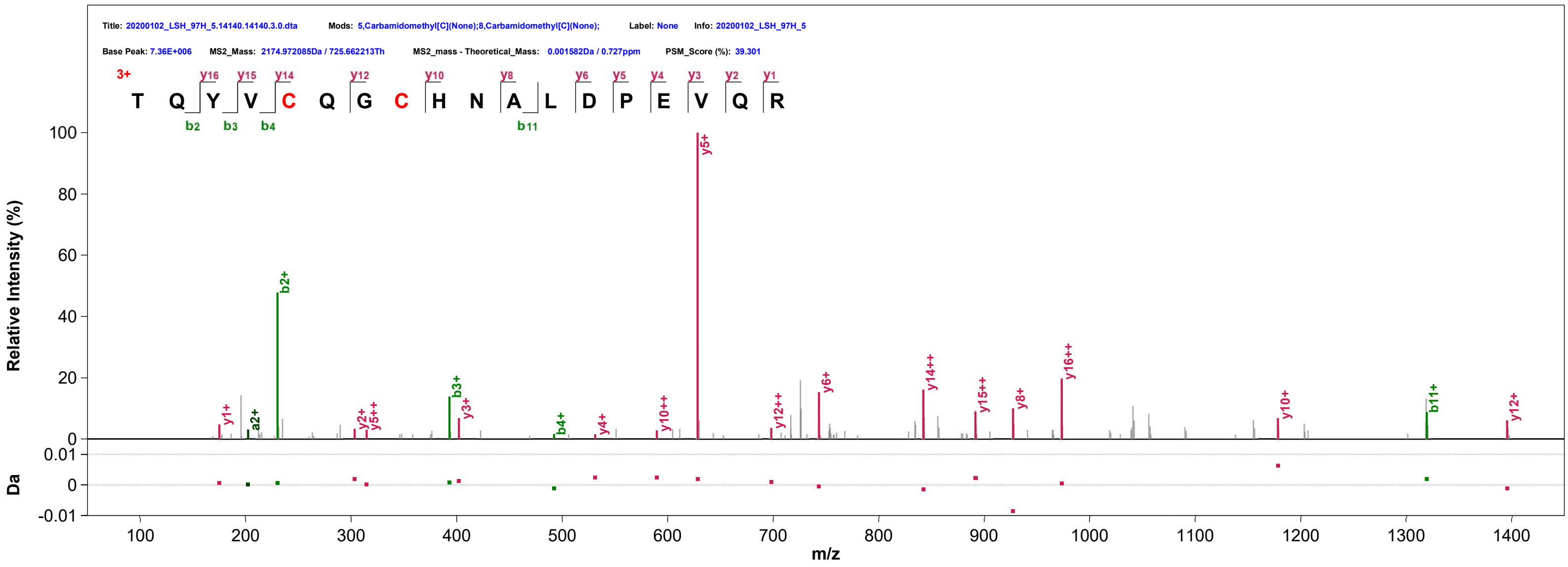

Protein Name: ZNF598_ORF_1
Peptide UUID: f414bf9c-8b48-400a-b2d1-4c729292d432_ZNF598_ORF_1_7

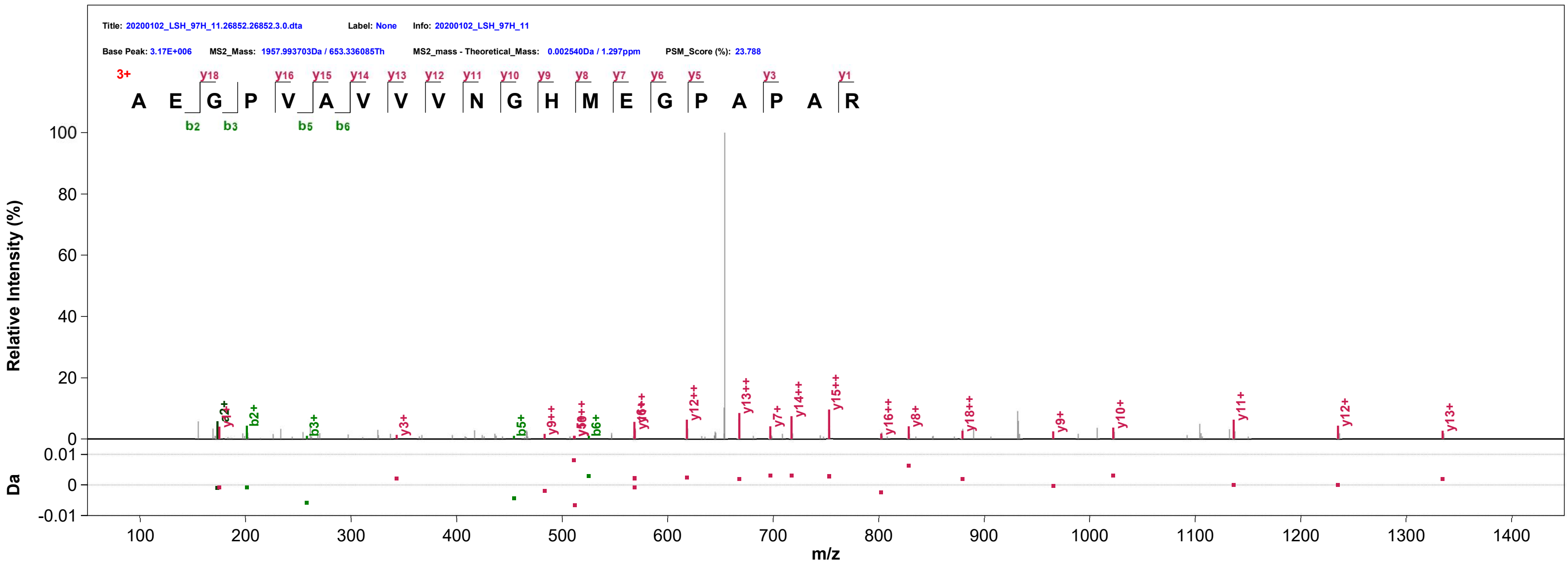

Protein Name: SART3_ORF_1
Peptide UUID: ffff6bd8-9141-4113-bd01-d4b9eee65a2f_SART3_ORF_1_1

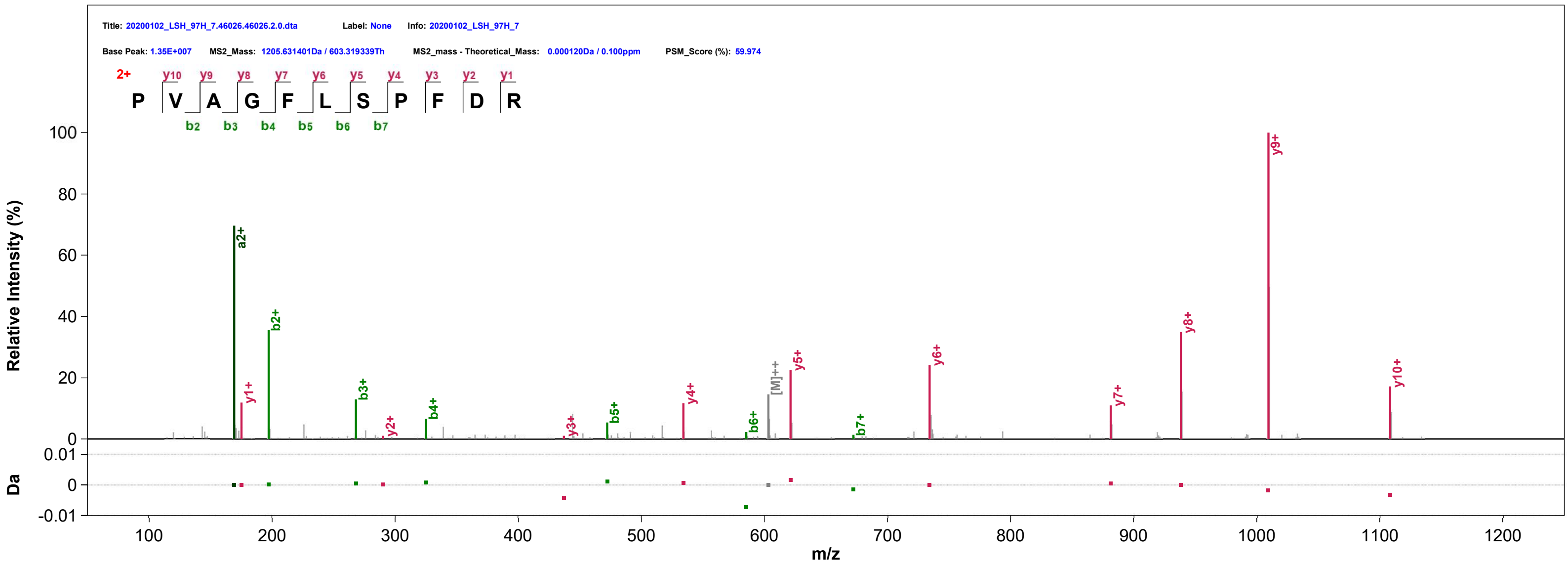

Protein Name: SART3_ORF_1
Peptide UUID: ffff6bd8-9141-4113-bd01-d4b9eee65a2f_SART3_ORF_1_2

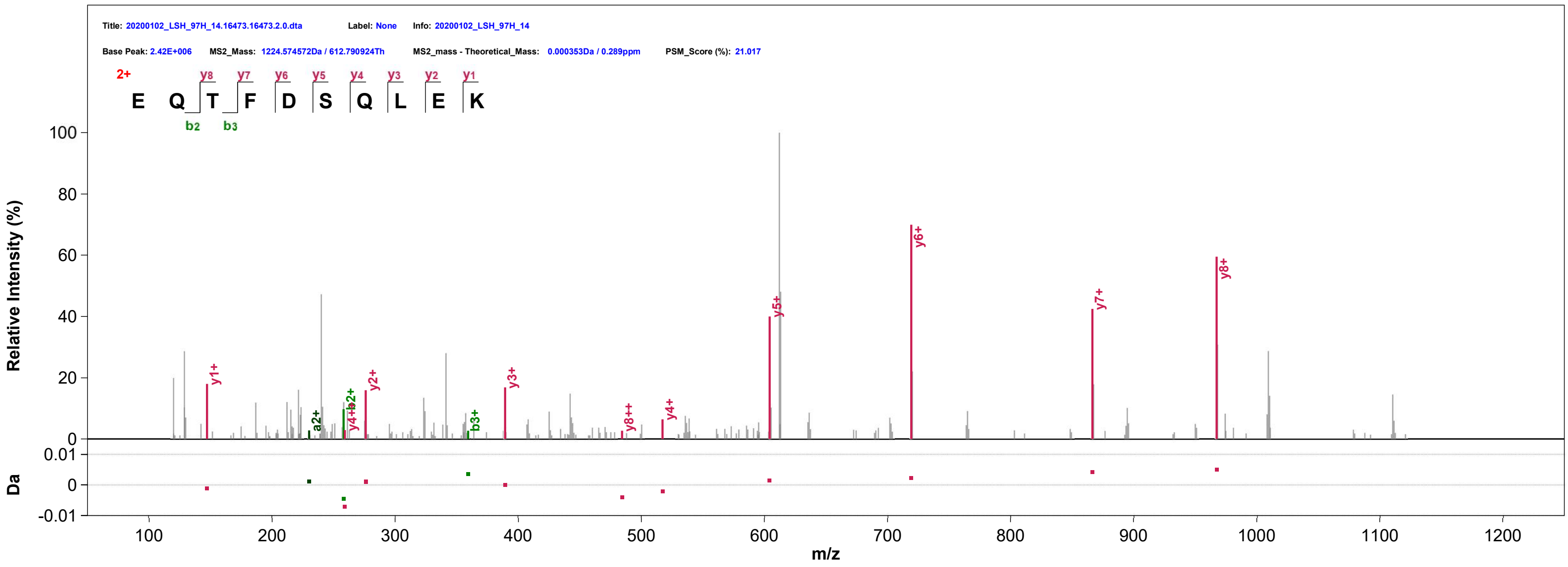

Supplement: Supplementary file 1 [file DataSheet2.pdf]
