# Supplementary figures and images for: Efficient Detection of the Alternative Spliced Human Proteome Using Translatome Sequencing
Source: Front Mol Biosci. 2022 Jun 2;9:895746. doi: 10.3389/fmolb.2022.895746 (PMC9201276; doi:10.3389/fmolb.2022.895746)

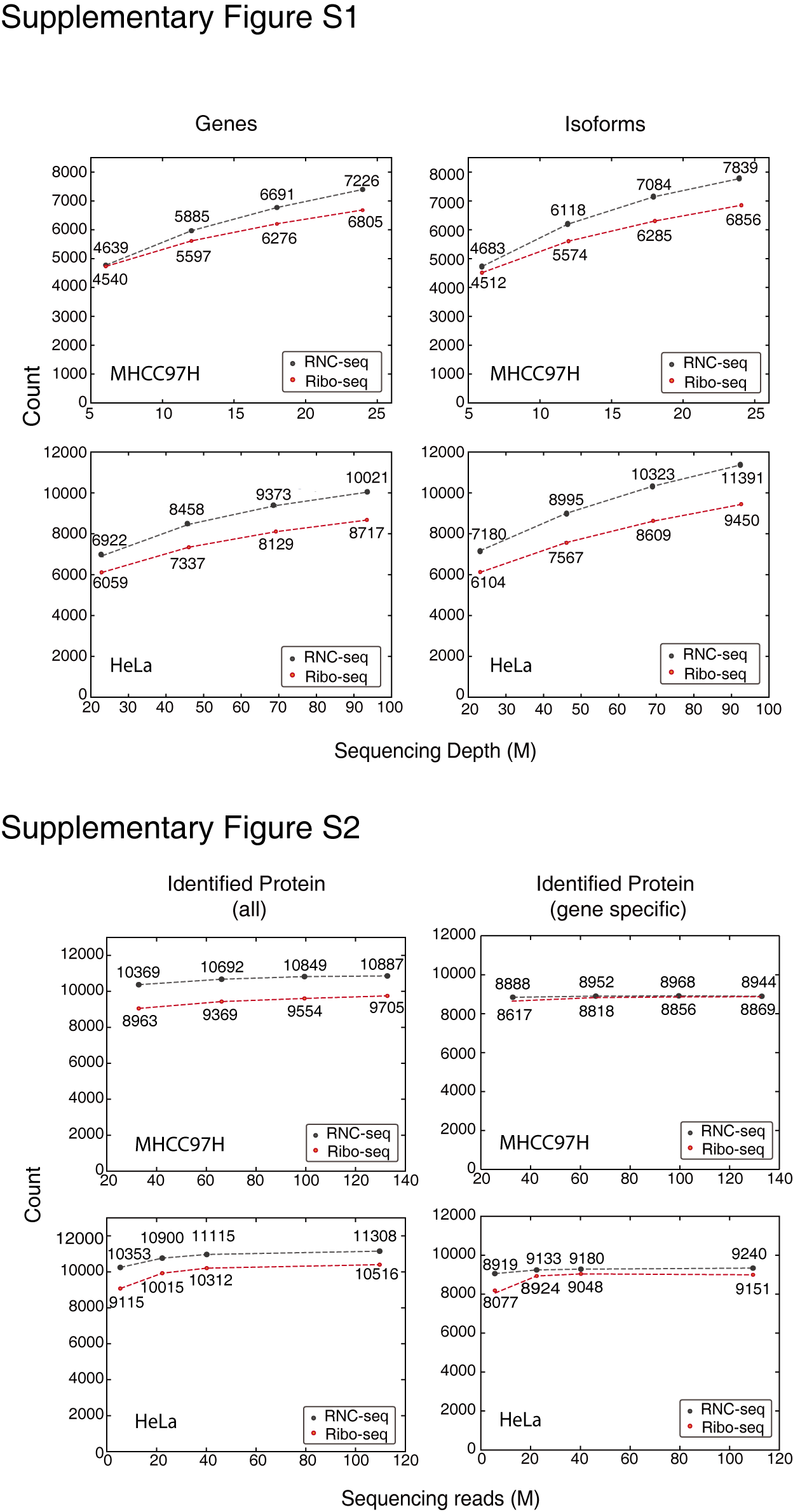

Supplement: Supplementary file 2 [file Image1.tif]
